# Prolonged growth and extended subadult development in the *Tyrannosaurus rex* species complex revealed by expanded histological sampling and statistical modeling

**DOI:** 10.7717/peerj.20469

**Published:** 2026-01-14

**Authors:** Holly N. Woodward, Nathan P. Myhrvold, John R. Horner

**Affiliations:** 1 Department of Anatomy and Cell Biology, Oklahoma State University Center for Health Sciences, Tulsa, Oklahoma, United States; 2 Intellectual Ventures, Bellevue, Washington, United States; 3 Schmid College of Science and Technology, Chapman University, Orange, California, United States

**Keywords:** Osteohistology, Dinosaur, Tyrannosaur, Growth, Life history, Statistics, Species complex

## Abstract

**Background:**

*Tyrannosaurus rex*, one of the most iconic non-avialan dinosaurs, remains a central focus of paleobiological research. Growth modeling suggests *T. rex* exceeded 8,000 kg within two decades and had a lifespan approaching 30 years. However, this understanding of *T. rex* growth dynamics is dependent on single-point histological sampling of multiple skeletal elements and lacks specimens encompassing the earliest growth states.

**Methods:**

We present the most comprehensive histological analysis of *Tyrannosaurus* ontogeny to date, based on transverse diaphyseal sections of femora and tibiae from 17 individuals ranging from small juveniles to large adults. Four alternative statistical models were tested, differing in the treatment of cortical growth marks, including annulus-like birefringent bands visible only in cross-polarized light. Due to high intraspecies morphological variability, the taxonomic status of many *Tyrannosaurus* specimens is debated, prompting use of the term “*Tyrannosaurus rex* species complex” to describe our dataset.

**Results:**

The best-supported model incorporated all visible growth marks, produced the narrowest confidence bands, and indicated lower maximum growth rates and a delayed attainment of asymptotic size (~35–40 years) compared with earlier estimates. We also find that two immature specimens within the *Tyrannosaurus rex* species complex are not statistically compatible with the other growth series. Our approach is the first in dinosaur skeletochronology to simultaneously estimate the position of the earliest preserved growth mark across specimens, while fitting sigmoidal curves with simultaneous confidence bands. We find the inclusion of double growth marks and those visible only with cross polarized light provide better statistical model fits and this may have implications for modeling other taxa. Additionally, we find no strong link from extant vertebrates to support the idea that the growth inflection point is biologically significant and corresponds to sexual maturity. Our results suggest that the *Tyrannosaurus rex* species complex grew more gradually and over a longer lifespan than indicated by prior models, with a protracted period of subadult development.

## Introduction

*Tyrannosaurus rex* (Osborn, 1905) is among the most iconic and scientifically impactful non-avialan dinosaurs known to paleontology. Ongoing research affords an increasingly nuanced understanding of its paleobiology, functional morphology, and ontogeny: cranial adaptations, including enlarged olfactory bulbs, a highly developed inner ear, and intricate trigeminal nerve branching suggest acute sensory capabilities (Witmer & Ridgely, 2010; Bouabdellah, Lessner & Benoit, 2022; Kawabe & Hattori, 2022) while biomechanical analyses of cranial function, bite force, and postcrania support interpretations of adult *Tyrannosaurus rex* as an agile, bone-crushing carnivore (Gignac & Erickson, 2017; Snively et al., 2019; Rowe & Snively, 2022). Additionally, intensive collection efforts since its initial discovery have enabled detailed morphological study, producing a “*Tyrannosaurus rex* species complex”; some researchers advocate a different taxonomic assignment for small-bodied specimens (Bakker, Williams & Currie, 1988; Larson, 2008, 2013; Witmer & Ridgely, 2010; Tsuihiji et al., 2011; Schmerge & Rothschild, 2016; Longrich & Saitta, 2024) and new species based on stratigraphic (Paul, Persons & Raalte, 2022; Paul, 2025) or geographic (Dalman et al., 2024) separation, while others argue that these differences reflect ontogenetic change (Carr & Williamson, 2004; Brusatte et al., 2016; Carr, 2020; Woodward et al., 2020; Voris et al., 2025) and that weak stratigraphic resolution hinders additional species assignment (Carr et al., 2022).

With respect to ontogeny, skeletal microstructure reveals rapid growth rates in *Tyrannosaurus*, with individuals reaching adult body masses of over 8,000 kg within approximately two decades and with an estimated lifespan of nearly 30 years (Erickson et al., 2004; Horner & Padian, 2004; Cullen et al., 2020; Persons, Currie & Erickson, 2020; Woodward et al., 2020). Here, we present a revised growth model for the *Tyrannosaurus rex* species complex, constructed from the most comprehensive ontogenetic sample analyzed to date and enabling a more complete reconstruction of life history.

The first quantitative models of *Tyrannosaurus* growth were based on skeletochronology (Erickson et al., 2004; Horner & Padian, 2004). In particular, the body mass model developed by Erickson et al. (2004) has served as a foundational framework for estimating the age and body mass of more recently discovered specimens (*e.g*., Cullen et al., 2020; Persons, Currie & Erickson, 2020), as well as for studies addressing ontogenetic morphological change (Carr et al., 2022), species abundance (Marshall et al., 2021), growth constraints (Persons, Currie & Erickson, 2020; Mallon & Hone, 2024), and phylogenetic relationships (Paul, Persons & Raalte, 2022; Longrich & Saitta, 2024; Paul, 2025). The (Erickson et al., 2004) model was based on seven individuals of varying ontogenetic stages and the authors derived age estimates from a range of skeletal elements (*e.g*., ribs, gastralia, limb bones), each with differing remodeling rates. Subsequent validation of bone histology shows that intraskeletal variability in growth mark preservation can compromise age estimates based on mixed-element sampling (*e.g*., Cullen et al., 2014; Woodward, Horner & Farlow, 2014; Heck & Woodward, 2021; Chapelle et al., 2022). Moreover, the (Erickson et al., 2004) growth curve included only a single specimen in the exponential (juvenile) phase of the sigmoidal model, with the remaining individuals situated in either the linear or asymptotic phases (Myhrvold, 2013). Consequently, growth dynamics during early and late juvenile stages remain poorly resolved. Additionally, Erickson et al. (2004) utilized the “whole bone” approach where each individual was represented by a single data point. In this method cortical growth marks are only counted to estimate age at death, ignoring size-at-age data that can be gleaned from interior growth marks, thus greatly restricting specimen utility for ontogenetic inferences.

In response to these limitations and owing to additional *Tyrannosaurus* fossil discoveries since (Erickson et al., 2004), we have assembled and histologically sampled the broadest ontogenetic series of *Tyrannosaurus rex* species complex tibiae and femora to date, yielding the largest dataset of transverse diaphyseal sections for this taxon. Our sample spans seventeen individuals, ranging from early juveniles to mature adults, and includes 12 transverse sections suitable for longitudinal growth modeling. By focusing exclusively on weight-bearing elements (femur and tibia) and using transverse sections rather than core samples or fragments, this study provides enhanced resolution and comparability in growth rate estimation across individuals. Importantly, our sample encompasses key transitional stages—early to late juvenile and early subadult—thus allowing for a more continuous and robust reconstruction of the life history and growth trajectory of *Tyrannosaurus*.

We show that during the annual period of active growth, *Tyrannosaurus* bone apposition rates were between 25 and 100 microns per day from early juvenile through late subadult stages, with slower rates (<10 microns per day) occurring only in the outer cortex of the largest individuals. Each specimen exhibited annual growth rate plasticity throughout ontogeny, and we modeled four variants of the dataset that differed in how cortical growth marks were identified and quantified. The model with the highest statistical support considered each growth mark as representing a single year hiatus and included annulus-like birefringent bands observed in cross-polarized light. The corresponding best-fit sigmoid growth curve contrasts with earlier models, showing subadult *Tyrannosaurus* growth was protracted, resulting in a growth asymptote at ~35–40 years of age, 15 years later than previously reported.

## Materials and Methods

### Specimen selection

Femur and tibia thin sections for this study are from seventeen individuals (Table 1), referred to *Tyrannosaurus rex* in museum collections. Slides include those from new specimens added to this study as well as those from specimens previously examined in Horner & Padian (2004). The *Tyrannosaurus rex* specimens in our study are from a geographically narrow area, with all but one having been collected from eastern Montana (BDM 050 was collected from North Dakota). However, because the taxonomic assignment of some *Tyrannosaurus rex* specimens is debated, going forward we refer to our sample as a *Tyrannosaurus rex* “species complex”, loosely adopting this biological term describing a genus in which the high intraspecific variability makes species designations ambiguous or controversial (see Liebers, Knijff & Helbig, 2004; Muñoz et al., 2013; Meik et al., 2015; Finkbeiner et al., 2025 for applications of the term “species complex”). Consistent with this usage, species complex is not intended to be synonymous with the group being monophyletic, but rather indicate that the systematics are not fully resolved.

**Table 1:** Tyrannosaurid specimens included in this study.

Specimen number	Museum-assigned taxonomic designation	Element sampled	Digital restoration?	Average slide thickness (microns) if prepared for this study
BDM 050	*Tyrannosaurus rex*	Femur, Tibia, Fibula	Yes, tibia	Femur, 180; Tibia, 250; Fibula, 90
BMRP 2002.4.1	*Tyrannosaurus rex*	Femur, Tibia	Yes, tibia	Tibia, 100
BMRP 2006.4.4	*Tyrannosaurus rex*	Femur, Tibia	No	Femur, 160
CCM V33.1.15	*Tyrannosaurus rex*	Tibia	No	125
DDM 35	*Tyrannosaurus rex*	Tibia	No	–
DDM 35.68	*Tyrannosaurus rex*	Tibia	No	–
MOR 009	*Tyrannosaurus rex*	Tibia	Yes	–
MOR 1125	*Tyrannosaurus rex*	Femur	Yes	–
MOR 1128	*Tyrannosaurus rex*	Tibia	Yes	–
MOR 1156	*Tyrannosaurus rex*	Tibia	No	–
MOR 1189	*Tyrannosaurus rex*	Tibia	Yes	250
MOR 1198	*Tyrannosaurus rex*	Femur	No	–
MOR 2949	*Tyrannosaurus rex*	Tibia	Yes	275
MOR 2970	*Tyrannosaurus rex*	Tibia	No	–
MOR 9756	*Tyrannosaurus rex*	Tibia	No	200
MOR 9757	*Tyrannosaurus rex*	Femur, Tibia	Yes, tibia	Femur, 240; Tibia, 250
USNM 555000	*Tyrannosaurus rex*	Tibia	Yes	–
TMP 1986.144.0001	*Gorgosaurus libratus*	Femur	No	245
TMP 1994.012.0602	*Gorgosaurus libratus*	Femur, Tibia	Yes, tibia	240

We were unable to enhance our sample size further with other previously published specimens, as thin section slides from the *T. rex* life history analysis of Erickson et al. (2004) have remained under study since that publication. We were also unable to include FMNH PR2081 in our sample, one of the largest specimens of *Tyrannosaurus*, because the core sampling methodology used to obtain cortical growth mark (CGM) circumferences (Cullen et al., 2020) greatly differs from ours (transverse sections) reducing confidence in growth curve comparisons (see Woodward et al., 2020 for discussion). For each individual, the choice of sampling femur or tibia was dependent on the material available for consumptive analyses. We limited our study to the femur and tibia for growth curve modeling because their circumferences can be translated to body mass using published methods such as Campione et al. (2014), and because femur and tibia circumferences of non-avialan theropods scale tightly with one another (D’Emic et al., 2023). While the latter relationship allowed us to combine femur and tibia datasets to increase ontogenetic sample size, for modeling *Tyrannosaurus rex* species complex growth our sample still consisted of 11 tibiae and only one femur. We also examined the femur and tibia bone microstructure of two *Gorgosaurus libratus* to permit qualitative growth comparisons between the *Tyrannosaurus rex* species complex and smaller tyrannosaurid taxa (see Table 1 for full specimen list and Table S1 for individual specimen raw data). Histology sampling permission for this study was granted by Greg Liggett (BLM), Josh Mathews (BMRP), John Scannella and Eric Metz (MOR), Denver Fowler (BDM), Nathan Carroll and Sabre Moore (CCM), Donald Brinkman and Brandon Strilisky (TMP). Daniel Barta and Roger Benson granted access to *Coelophysis* thin sections. Carrie Levitt-Bussian granted access to *Allosaurus* thin sections.

### Thin section processing

Specimens of *Tyrannosaurus* and *Gorgosaurus* were sampled transversely within the diaphysis (de Buffrénil, Quilhac & Castanet, 2021) inferior to the fourth trochanter (femur) and fibular crest (tibia). Specimen documentation included photographs, line drawings, 3D laser surface scans, and photogrammetry. Molds and casts of the sampled diaphyseal pieces were also produced upon institutional request. Sample and thin section processing followed the protocol of Lamm (2013), briefly described here. Depending on specimen size, initial cuts to remove diaphyseal samples were done with a Buehler Isomet 1000 wafering saw, Husqvarna Tilematic tile saw, or a Jet bandsaw, each fitted with a continuous diamond blade. The samples were embedded in Silmar 95BA-41 polyester resin for stabilization. From the cured resin blocks ~3 mm-thick wafers of embedded bone were removed, using the appropriate saw, and glued to frosted plastic slides with Starbond medium viscosity cyanoacrylate glue. In some cases, diaphyseal transverse section wafers were too large for a single 2″ × 3″ or 2.5″ × 3.5″ plastic slide. Those wafers were cut into smaller pieces using the tile saw, and each wafer segment was then glued to a plastic slide. Kerf loss associated with each cut was approximately 2 mm. Slides were hand polished using progressively finer grit silicon carbide papers on a Buehler Ecomet 4 grinder-polisher to a final thickness between 80 and 200 microns (Table 1) dependent upon the thickness at which optical clarity was achieved.

### Thin section imaging

Digital slide images were obtained at the Museum of the Rockies (MOR) or Oklahoma State University Center for Health Sciences (OSU-CHS). At MOR, slides were imaged using a Nikon Optiphot2-POL polarizing microscope, Nikon DS-Fi-2 digital camera, and Prior motorized stage. Photomontages of each slide were assembled using Nikon NIS Elements: Basic Research. Imaging at OSU-CHS was done using a Nikon Eclipse polarizing microscope, Nikon Fi2 digital camera, and ASI motorized stage. Photomontages were assembled in the software package Nikon NIS Elements: Documentation. To aid in growth mark identification, slides were imaged in plane polarized light (PPL) and in circularly polarized light (CPL). For transverse sections comprised of more than one slide, each thin section was imaged as described above and digitally reassembled using Photoshop CC. The measure tool was used to adjust the spacing between individual slides to accommodate kerf loss. Requests for documentation, photogrammetry files of specimens, full resolution digital thin section images, and Photoshop files can be made to the respective institutions where sampled specimens are reposited (Table S1). Digital thin section images are also accessible on Figshare (https://doi.org/10.6084/m9.figshare.29944892.v1).

**Figure 1:**
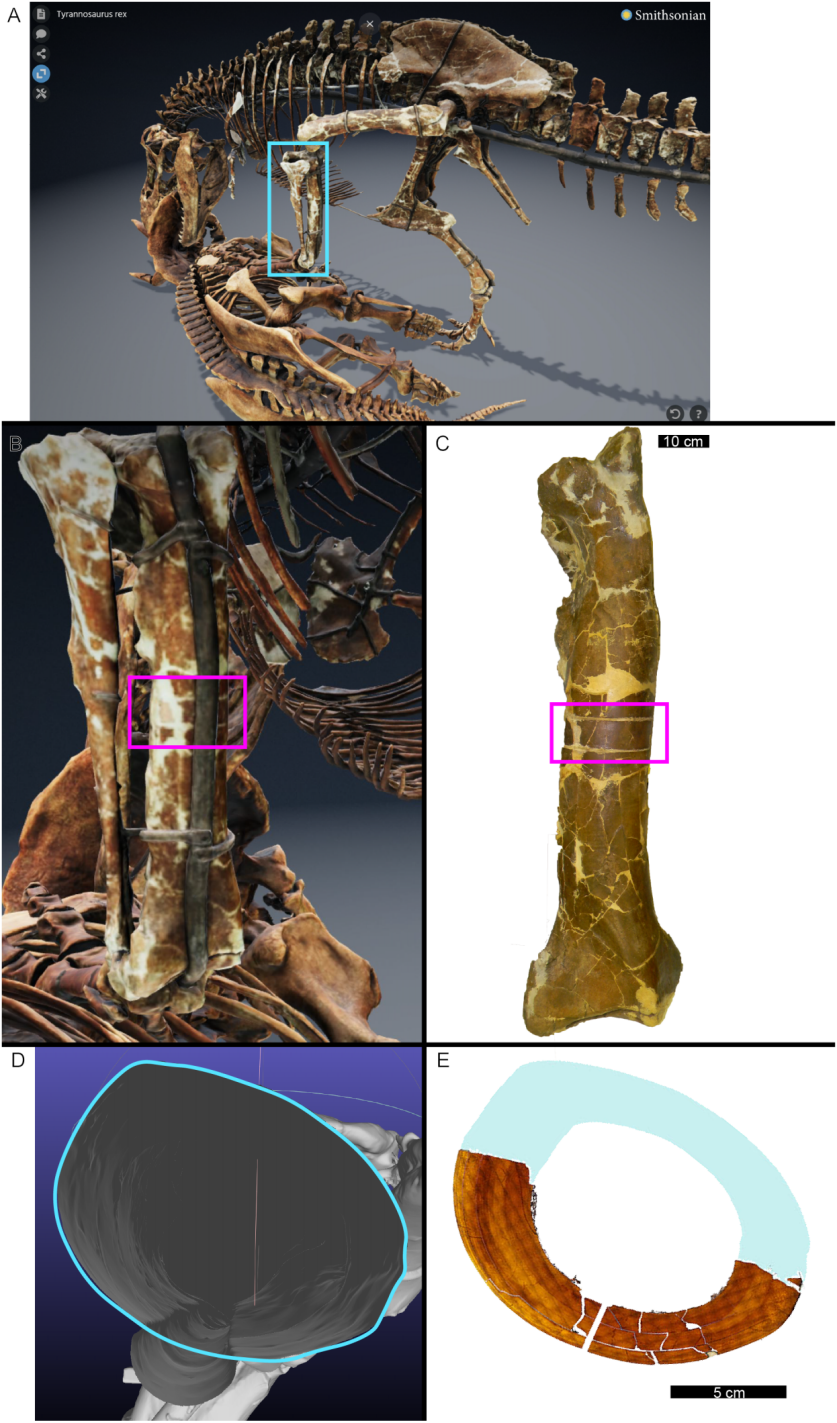
The cortical perimeter of the left tibia from USNM 555000 was reconstructed using the digital skeleton publicly available from Smithsonian 3D Digitization (https://3d.si.edu/). (A) Screen-capture of the *Tyrannosaurus* digital model. Blue box indicates the region of interest in panel B, which includes the left tibia. (B) The digitized left tibia. The purple box indicates the region of sample removal and replica cast restoration. (C) Photograph of left tibia after replica cast restoration. Purple box indicates the same region visible in panel B. (D) A transverse slice through the digital model was obtained at the region of interest indicated in panels B and C using Meshlab software. A screen capture of the slice was imported into Photoshop CC and the perimeter digitally traced using the pencil tool (blue outline). (E) The layer of digital tracing was fitted to the digitized thin section of tibia USNM 555000 to restore the missing cortex, indicated in blue.

In many cases, the diaphysis of a specimen was diagenetically crushed. Transverse sections of such diaphyses were reassembled from fragments in Photoshop CC using layers to incrementally restore the circular shape of the cortex using only rigid motion of the fragments without deformation (Table 1). For MOR 1128 and USNM 555000 (formerly MOR 555) only a partial tibial transverse section had been removed for earlier histology studies and a cast replica of the portion removed had been restored to the respective tibia. The skeleton of USNM 555000 was later digitized and made publicly available (https://3d.si.edu/object/nmnhpaleobiology_10250729, last accessed August 2025). The sampling location is visible on the skinned model, making it possible to obtain an associated digital transverse section using Meshlab (Cignoni et al., 2008) software. The image of the cross-section outline produced in Meshlab was imported as a layer in Photoshop CC and fitted to the partial tibia photomicrograph of USNM 555000 using the scale transformation command, completing the shape of the periosteal surface (Fig. 1). Again, using the scale transformation command, this outline layer was subsequently used to complete the periosteal surface of the partial transverse section of MOR 1128. In both cases, the diaphyseal circumferences (or more properly, perimeters) measured from the digital periosteal surface restorations approached measurement values taken with a flexible tape measure prior to sample removal.

### CGM identification

Since the results of our study are dependent on the identification of cortical growth marks, their morphology is described here. Skeletal growth marks (*sensu* de Buffrénil & Quilhac, 2021) include the cyclical and non-cyclical marks in tooth enamel, tooth dentine, tooth cementum, and bone. In turn, cortical growth marks (CGM) are specific to endochondral and intramembranous bone and include lines of arrested growth (LAG) and annuli. LAG and annuli result from (typically annual) decreases in cortical bone apposition rate (see de Buffrénil & Quilhac, 2021 for a review). Thus, CGM record the diaphyseal cross-sectional shape of the periosteal surface during the annual hiatus (de Buffrénil, Quilhac & Castanet, 2021)). The periodicity of LAG and annuli is well-documented in extant vertebrate taxa (Klevezal, 1996; Köhler et al., 2012; de Buffrénil & Quilhac, 2021, and references therein) and CGM are utilized extensively in extant and extinct vertebrate skeletochronology, while the zones of primary bone tissue between CGM are used to estimate the apposition rates between hiatus events.

In more detail, a LAG forms at the periosteal surface during appositional growth arrest (de Buffrénil & Quilhac, 2021). It is a ~10 micron-thick, hyper-mineralized line within the bone cortex, visible once periosteal apposition resumes. In contrast, an annulus represents a period of decreased apposition relative to the surrounding primary matrix, resulting in a diffuse ring rather than a distinct line (de Buffrénil & Quilhac, 2021; de Buffrénil, Quilhac & Castanet, 2021). The presence of annuli and LAG are not mutually exclusive (de Buffrénil, Quilhac & Castanet, 2021), and one CGM type can also transition into the other at different locations about the cortex (Pereyra et al., 2024).

**Figure 2:**
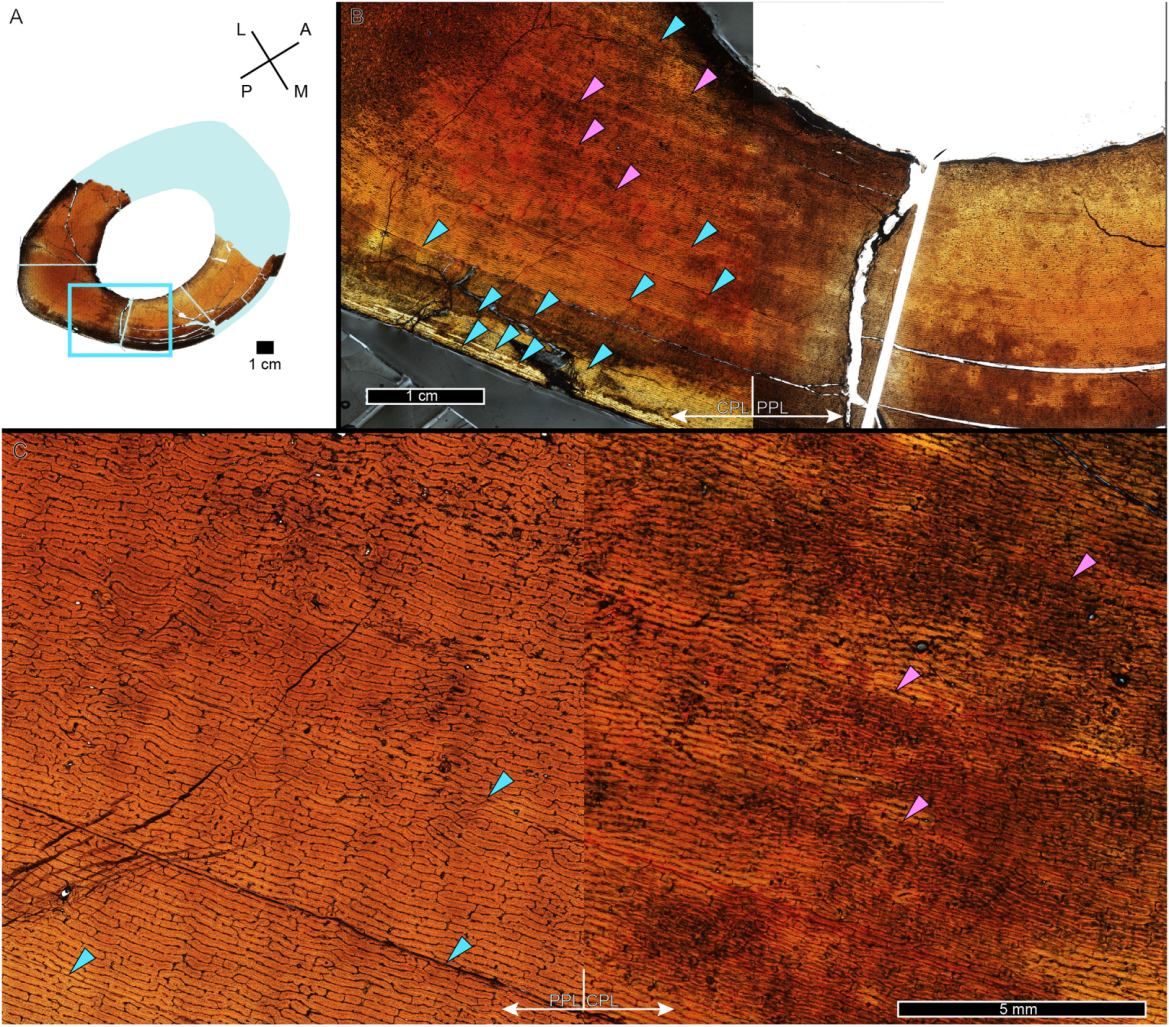
Some CGM within the tibia of MOR 1128 are only visible as birefringent annuli in CPL. (A) Tibia transverse section, imaged in PPL. The light blue shading represents missing cortex. Box indicates region of interest in panel B. (B) Posteromedial cortex, with left half of image in CPL and right half of image in PPL. Purple arrows point to CGM only visible in CPL and blue arrows point to CGM visible in PPL. (C) Enlarged region of panel B. The left half of the image is in PPL, while the right half is imaged in CPL. Purple arrows point to CGM only visible in CPL, while blue arrows point to CGM visible in PPL. CGM, cortical growth mark; PPL, plane polarized light; CPL, circularly polarized light. Cross section orientations: A, anterior; M, medial; P, posterior; L, lateral.

While LAG may be readily observed in bright field or plane polarized light (PPL) as solid thin lines, annuli are more difficult to discern due to their diffuse appearance. In PPL annuli may be recognized as localized bands of differing vascular canal organization, reduced vascular canal and osteocyte lacuna density, and/or flattened osteocyte lacunae. An annulus is commonly formed of parallel-fibered tissue within a woven matrix, or of lamellar tissue if within a parallel-fibered matrix (de Buffrénil & Quilhac, 2021). As a result, in cross-polarized light (XPL) annuli are more readily identified than in PPL because the parallel or lamellar fiber arrangement results in higher birefringence compared to the less organized fiber matrix to either side (Bromage et al., 2003; de Buffrénil & Quilhac, 2021). Indeed, within our sample, some annuli were only visible when viewed in cross-polarized light (XPL). Because these anisotropic fibers remain parallel to the plane of section while forming the circular annulus, at regular 90-degree intervals the fibers do not interfere with the transmission of polarized light and remain dark, or extinct. We therefore used circularly polarized light (CPL) which allowed us to more easily differentiate annuli from surrounding regional variations in tissue matrix birefringence (Fig. 2). Circularly polarized light (CPL) is cross-polarized light with the addition of two quarter-wave plates which eliminate the periodic light extinction, resulting in birefringence of the annulus over its entire 360-degree arc (Bromage et al., 2003).

### Multiplets

**Figure 3:**
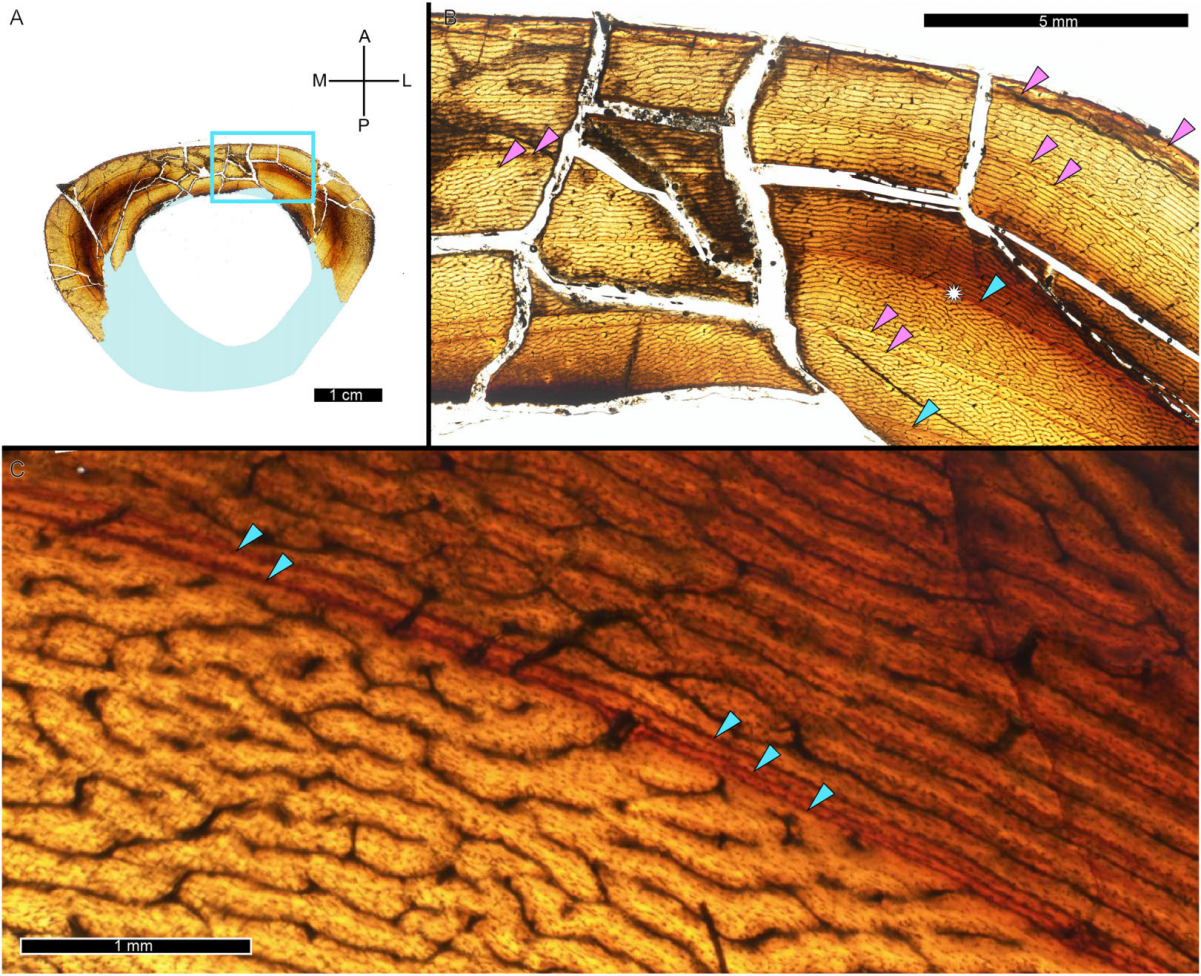
Visual comparison between multiplet CGM and a double-triple CGM in the tibia of MOR 1189. (A) Tibia transverse section. Light blue shading represents missing cortex. Box indicates the region of interest in panel B. (B) Anterolateral region of tibia cortex. Blue arrows point to single CGM and purple arrows point to multiplet CGM, the latter identified by a grouping of two or more CGM in closer proximity to one another than to adjacent CGM. The asterisk indicates the region of interest in panel C. (C) A double-triple CGM, defined by splitting and merging of one CGM. On the right, three lines are visible (three arrows), and towards the left, they merge into two lines (two arrows). All panels in plane polarized light. CGM, cortical growth mark. Cross section orientations: A, anterior; M, medial; P, posterior; L, lateral.

A zone of bone matrix may be thinner (*i.e*., CGM are more closely spaced) than the preceding or following zone. The CGM bounding the zone are either parallel to one another over their circumferences or the zone may result from a single CGM splitting into one or more lines about the cortex (*e.g*., Snover & Hohn, 2004). The literature uses the term ‘double LAG’ to refer to merging CGM as well as closely-spaced parallel CGM, with the latter configuration renamed a ‘doublet’ in recent paleohistology studies (*e.g*., Lee & O’Connor, 2013; Zanno et al., 2019; Funston & Currie, 2021; Qin et al., 2021; Jurašeková et al., 2022; Krumenacker, Zanno & Sues, 2022) or ‘couplet’ (Jevnikar, 2022) and as ‘double/multi-cortical growth marks’ (D’Emic et al., 2023). The terms doublet and couplet imply two lines, but we frequently observe three or even four parallel CGM (*i.e*., more than one zone). For this reason, here we refer to two or more CGM with thinner zones relative to adjacent zones as a multiplet (see Discussion), and use ‘double LAG’ to refer to CGM that split and merge over their circumference (Fig. 3).

### CGM quantification

To model growth of each *Tyrannosaurus* specimen, CGM as well as cortical endosteal and periosteal surfaces were traced from the digitized transverse thin section PPL and CPL images using layers in Photoshop CC and the pencil tool. CGM within multiplets were traced individually, and annuli only visible in XPL were also included in the CGM dataset. In some cases, CGM were incomplete owing to medullary expansion, secondary remodeling, or taphonomic preservation (*e.g*., cracks or missing fragments). If enough of a CGM remained to reasonably estimate the shape, we used the nearest complete CGM as a template and scaled to fit the visible remainder of the partial CGM (de Buffrénil, Quilhac & Castanet, 2021). On the other hand, if a CGM was only visible a short distance such that its general shape was unclear, its presence was counted but restoration was not attempted.

**Figure 4:**
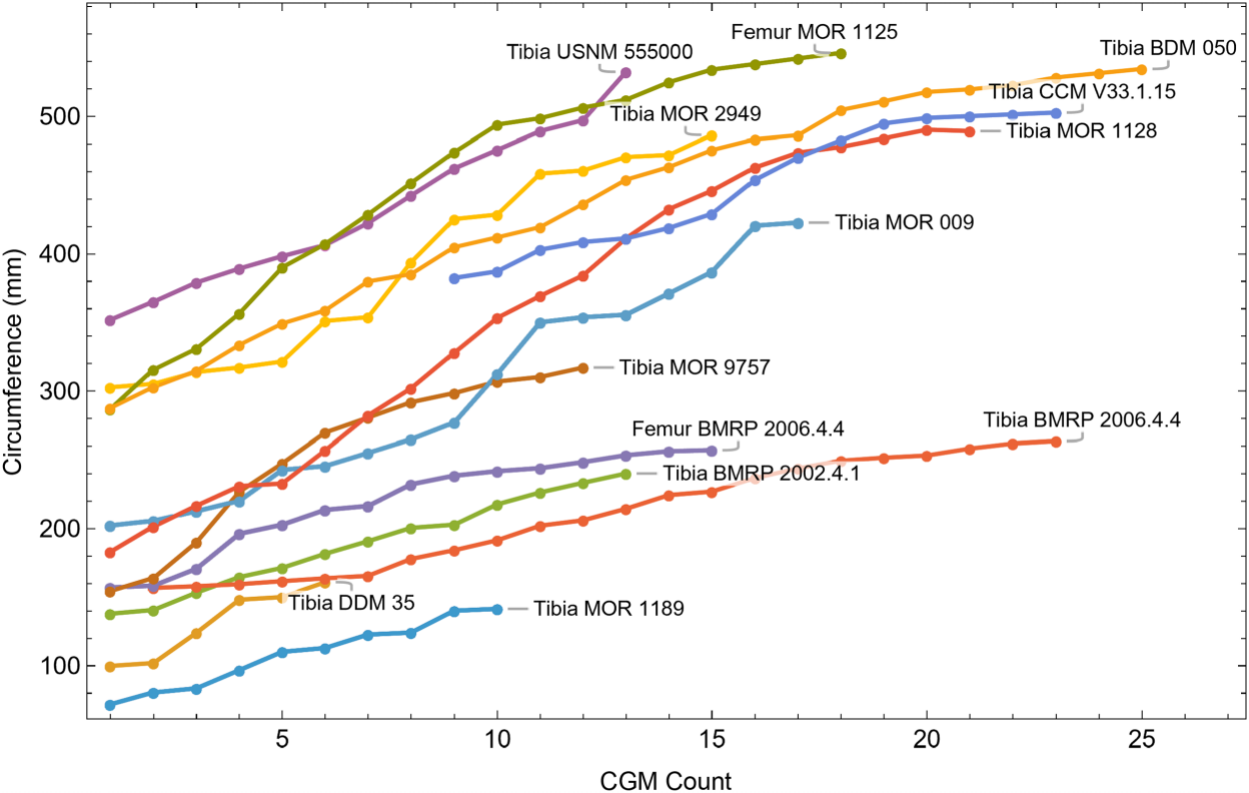
Plot of the growth series for *Tyrannosaurus*, dataset Trex1, variant A. The lines plot raw growth series obtained from measuring CGM on thin sections of femora and tibiae of *Tyrannosaurus*. The CGM growth series for each specimen consist of a set of CGM counts (x-axis) and CGM circumference (y-axis): see Eqs. (1), (2). These data include all CGM found in the specimens (Table S1). Plots for other variants in Figs. S3, S4.

Once CGM were identified and traced, Fiji (Schindelin et al., 2012) and the BoneJ plugin (Doube et al., 2010) was used to quantify the geometric centroid of the transverse section, CGM circumferences and inclusive areas of each CGM, and circumferences of the periosteal and endosteal surfaces (after Woodward et al., 2015). Distances from the geometric centroid to the periosteal surface, endosteal surface, and to each CGM along major and minor axes were quantified in Photoshop CC using the measure tool. From these measurements, an ontogenetic dataset was produced for each *Tyrannosaurus* specimen in the study included in growth modeling (Table S1). The same data were collected for *Gorgosaurus* (Table S1) but since there were only two specimens available for analysis, growth modeling was not performed. The growth series for variant A of *Tyrannosaurus* are plotted in Fig. 4, other variants in Figs. S1–S6, *Gorgosaurus* in Figs. S7, S8.

### Growth modeling

The femur and/or tibia histology of seventeen individuals in the *Tyrannosaurus rex* species complex contributed to qualitative assessments of life history. Unfortunately, the diaphyses of five specimens were too fragmentary (MOR 1156, MOR 1198, DDM 35.68) or taphonomically damaged post-burial (MOR 2970, MOR 9756) to accurately quantify CGM or circumferences from thin sections and were therefore omitted for modeling. However, the qualitative nature of these specimens is consistent with the others. The Wolfram Mathematica software package (Wolfram Research, Inc., 2024) was used to perform growth modeling using the twelve remaining individuals in the dataset which were represented by either a femur or tibia, and by both femur and tibia in the case of BMRP 2006.4.4.

Due to medullary cavity expansion during ontogeny, observed CGM constitute an incomplete record of life history, and the absolute age of a specimen must be estimated. For each dataset, the age of the earliest preserved CGM in each growth series was set relative to that of MOR 1189, the growth series with the smallest circumference CGM. A least squares clustering (Myhrvold, 2013) was applied to determine the optimal overlap between the specimen datasets and to estimate the youngest (starting) age preserved in each growth series. Once age at the first observed CGM was estimated for each specimen, the growth series for that specimen was shifted so that the first CGM lined up with its estimated age. Nonlinear ordinary least squares (OLS) equations are typically used to fit a sigmoid curve to the growth series data. Here, we instead applied a novel approach. Rather than estimating the starting ages as the initial step followed by nonlinear OLS as is conventional for modeling dinosaur growth, we treated starting ages as independent variables and estimated them at the same time as the sigmoid function parameters using least squares optimization.

The best fit curve from the *Tyrannosaurus rex* species complex dataset was dependent upon how CGM within multiplets and CGM identified as XPL-only annuli were treated within each specimen growth series. Therefore, we developed four variants of the dataset, each using a different interpretation and mapped observed histology to CGM data for modeling. The variant “A” considered all of the marks: XPL annuli, conventional CGM (*i.e*., LAG and annuli visible in PPL), and each CGM within a multiplet to represent a unique annual hiatus. Variant “NoM” (for no multiplets) counted XPL-only annuli and conventional CGM as annual but considered a multiplet grouping to represent a single hiatus event. For the “NoX” variant, each CGM in a multiplet and each conventional CGM were counted individually but XPL annuli were excluded. Finally, the “NoXM” variant matches the previous practice in dinosaur growth literature which omits CGM deemed to be multiplets, and which have not reported the existence of XPL-only annuli. Thus, each growth series had a different CGM count depending upon the dataset variant considered. The counts of multiplet and XPL-only annuli for each specimen appear in Table S2 and the series are plotted in Figs. S1, S2. Separate analyses for each variant were maintained to test the characteristics of the underlying histological interpretations, which is particularly important because some interpretations are newly proposed here. Nine different sigmoidal growth curves were produced to increase the likelihood of finding the fit that most closely matched the datasets. For diagnostic purposes, linear and quadratic non-sigmoidal curves were also fitted, and full lists appear in Tables S3, S4. Finally, the corrected Akaike information criterion (AICc) was used for model selection (Burnham & Anderson, 2002). Statistical uncertainty was then determined by estimating the 95% simultaneous confidence band (CB) for each of the best fits using parametric bootstrap methods (Montiel Olea & Plagborg-Møller, 2019). For performing the bootstrap resampling, fractional random weighting (FRW) bootstrap was used (Xu et al., 2020). In addition to arithmetic fitting, logarithmic fitting was performed to account for heteroskedasticity in the data and to more equally balance the distribution of errors. Lastly, earlier studies sometimes converted from CGM circumference to body mass using a correlation (Campione & Evans, 2012) before curve fitting. However, we preferred to perform the growth analyses on circumference data then later convert to mass if needed (Myhrvold, 2013).

## Results

### Bone microstructure

The primary cortical matrix of femora and tibiae from all *Tyrannosaurus* and *Gorgosaurus* specimens studied here was a woven-parallel complex separated into zones by cortical growth marks (see de Buffrénil & Quilhac, 2021 for bone tissue terminology). Multiplets were observed in all but one specimen, and XPL-only annuli were present in most specimens (Figs. S1, S2 and Tables S1, S2). Vascularity was plexiform to laminar, with reticular canals somewhat more common in the smaller individuals. A well-developed external fundamental system (EFS), indicating skeletal maturity (de Buffrénil & Quilhac, 2021), was observed in *Gorgosaurus* TMP 1994.012.0602. *Tyrannosaurus* tibiae CCM V33.1.15 and BDM 050 exhibit possible incipient EFS: in each case the outermost cortex is somewhat less vascularized than the rest of the cortex and contains several closely spaced CGMs within lamellar tissue.

Despite avoiding sampling on bony processes when possible, narrow columns of secondary osteons were present in both femur and tibia cortices and were likely associated with ligament or muscle attachment. Outside of these localized regions, there was virtually no Haversian remodeling in the two *Gorgosaurus* specimens and dense Haversian systems were only present in ontogenetically older *Tyrannosaurus*. In such cases typically only CGM within the innermost cortex were obscured by secondary osteons to such a level that reconstructing CGM shape was difficult or impossible.

For one specimen, *Tyrannosaurus* BDM 050, the ipsilateral femur, tibia, and fibula were made available for histological analyses. This afforded a unique opportunity for intraskeletal osteohistological comparisons of hindlimb elements. As with other specimens in our sample, BDM 050 femur and tibia bone microstructure were of a zonal plexiform to laminar woven-parallel complex from inner to outer cortex. Secondary osteon frequency within the inner cortex of the femur was 10–15% higher than that of the tibia. In contrast, the entire cortex of the fibula was remodeled by numerous secondary osteon generations. Slow-forming lamellar tissue was infrequently visible interstitially at the periosteal surface of the fibula. No EFS was observed in the femur. While a possible EFS was visible in the tibia, not enough primary tissue was visible in the fibula to determine whether the lamellar tissue at the periosteal surface was an EFS or was simply a product of slow diaphyseal apposition in late ontogeny.

### Dataset curve fitting

Biological growth studies are based on a framework that assumes growth of the organism follows a smooth mathematical curve, typically a sigmoid, on which random variations are superimposed. These variations include any factor that could affect growth such as individual genetic differences, environmental or resource factors, illness, injury or pathology, or sexual size dimorphism. A growth curve for an individual specimen will express properties unique to it, while a growth curve for a population will average out individual differences to yield typical population growth behavior. Statistical methods are then used to produce the curve and associated uncertainty, which removes the random variations to the best extent possible. This paradigm has been widely successful across many biological domains, including plant, animal, bacterial colony, and even tumors (Benzekry et al., 2014; Kawano, Wallbridge & Plummer, 2020), providing strong motivation for its application to modeling dinosaur growth.

For each dataset variant, a *Tyrannosaurus* CGM growth series *gs* is a list of *N* pairs $\left( {cgm{c_i},siz{e_i}} \right)$ where $cgm{c_i}$ is the CGM count for the *i*^th^ CGM, which has a size parameter $siz{e_i}$.

(1) $$gs = \left( {\left( {cgm{c_1},siz{e_1}} \right),\left( {cgm{c_2},siz{e_2}} \right), \ldots \left( {cgm{c_N},siz{e_N}} \right)} \right).$$

Here, the size parameter for primary data is the CGM circumference, so $siz{e_i}$ will be referred to as $cir{c_i}$.

Typically, the CGM count increases by 1 between each pair in series, *i.e.*, $cgm{c_{i + 1}} - cgm{c_i} = 1$, consistent with the assumption that a CGM records an appositional decrease or pause after one year of growth (see section on CGM identification above). However, in some specimens we found that a CGM circumference could not be accurately estimated individually but the existence of these CGM could be counted. This occurred, for example, in the innermost cortex where CGM were almost wholly destroyed due to medullary expansion, in outermost cortex of larger specimens where CGM spacing was compressed, or when CGM were only partially visible due to taphonomic periosteal surface erosion. In such situations the growth series records only the CGM with measurable circumferences, skipping those that could not be measured but including them in the CGM count. In that case, $cgm{c_{i + 1}} - cgm{c_i} > 1$, will occur for some values of *i*.

Conversely, there were cases where remodeling of the medullary cavity left countable evidence of earlier CGM but not enough remained intact to estimate a circumference. In such a case $cgm{c_1} > 1$. When there are M specimens of the same taxon, Eq. (1) is generalized as follows.

(2) $$\eqalign{ {gs_j} & = \left( {\left( {cgm{c_{1,j}},siz{e_{1,j}}} \right),\left( {cgm{c_{2,j}},siz{e_{2,j}}} \right), \ldots \left( {cgm{c_{N,j}},siz{e_{N,j}}} \right)} \right),\quad j = 1, \ldots M \\ starts & = \left( {star{t_1},star{t_2},star{t_3}, \ldots star{t_M}} \right),\quad star{t_1} = 0 \\ gs{s_j} & = \left( {\left( {cgm{c_{1,j}} + star{t_j},siz{e_{1,j}}} \right), \ldots \left( {cgm{c_{N,j}} + star{t_j},siz{e_{N,j}}} \right)} \right) \\ agesizedata & = \bigcup\limits_{j = 1}^M g s{s_j}.}$$

For each growth series $g{s_j}$ a starting age $star{t_j}$ must be estimated. Since absolute age of a specimen cannot be determined due to medullary cavity remodeling, we adopt the convention of setting $star{t_1} = 1$, so the *starts* are the starting ages relative to a first growth series, MOR 1189, as it has the smallest circumference CGM. The starting age $star{t_j}$ is applied to a growth series by adding the start to each of the $cgmc$, forming a growth series with starts $gs{s_j}$. The union of these sets is the age and size data that is used to fit a growth curve.

Earlier dinosaur growth studies estimated starting ages using several *ad hoc* methods including examining the curves by eye, or by various ‘retrocalculation’ methods involving nesting the thin section slides by size to see where CGM between specimens might overlap (*e.g*., Chinsamy, 1993; Horner & Padian, 2004; Bybee, Lee & Lamm, 2006). Using growth records from multiple specimens in this way resulted in a composite growth series that better modeled the population as a whole and contained a longer span of data than that provided by any individual specimen. Unfortunately, these methods were subjective and prone to error. For example, matching thin section contours visually requires making comparisons between curves that are not the same shape. Here we similarly seek to produce a composite growth series but do so by applying improved and objective algorithms.

**Figure 5:**
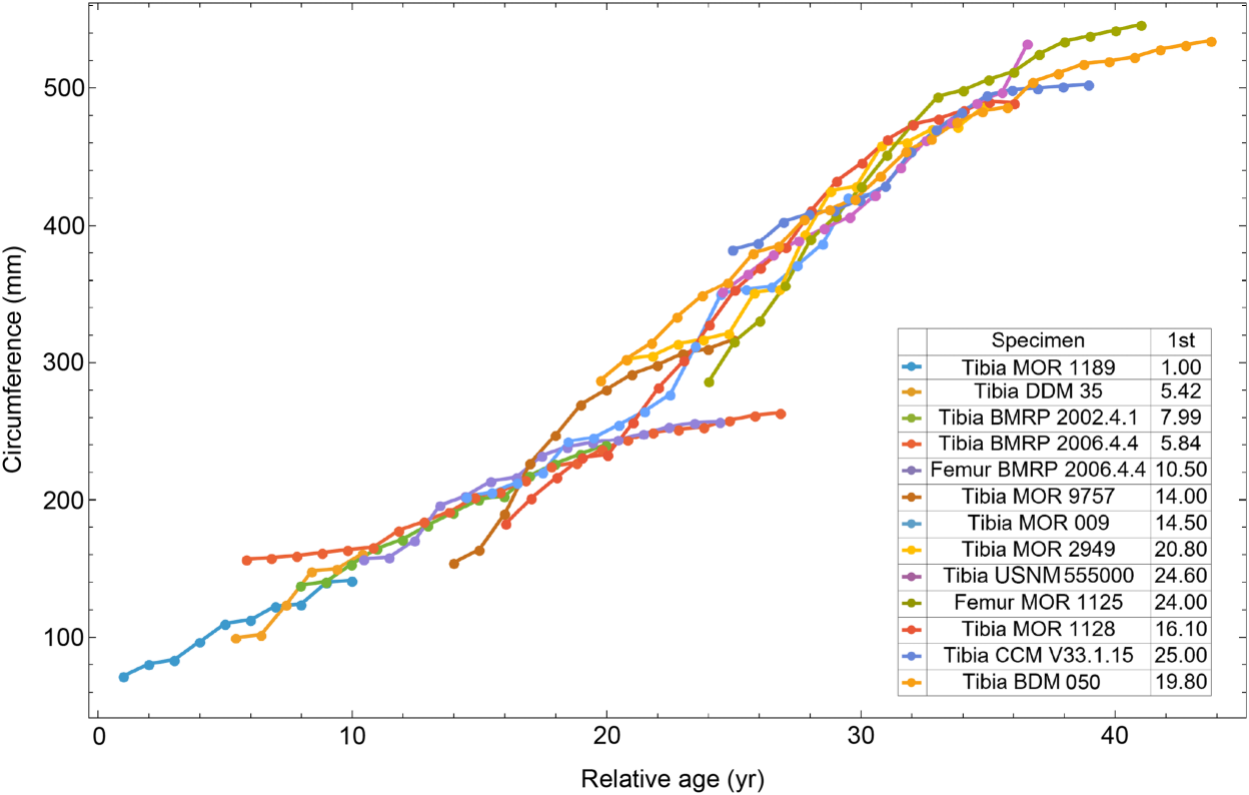
Starting ages of the growth series in the Trex1 A dataset are assigned to minimize series-to-series overlap distance. For each specimen, the starting CGM age is estimated using least squares minimization, and the numerical values are found in the inset table in the column labeled “1st”. All ages are in years relative to the starting age of the smallest circumference specimen, Tibia MOR 1189.

Myhrvold (2013) introduced the first algorithmic method, which uses a least squares clustering to determine the optimal overlap between the specimens (Fig. S9). An application of this method to the growth series in Fig. 1 is shown in Fig. 5 (see also Figs. S10, S11). This least squares minimization method has several weaknesses. First, it only works if each growth series is overlapped by one or more other growth series, and these mutually overlapping series must include the entire dataset. There is no guarantee that these requirements are met, although it happens that the *Tyrannosaurus* series have sufficient CGM count and overlap to have this property. A second and more detailed objection is that the least squares minimization prioritizes overlaps between individual growth series proportional to the amount of overlap present. Despite the potential disadvantages, an objective algorithm is preferred to a subjective placement by eye.

After starting ages are applied, the next step is nonlinear ordinary least squares (OLS) curve fitting. Sigmoidal growth curves $f\left( {a,b,c,t} \right)$ are used to fit the age, circumference data points ( $agesizedata$ from Eq. (2)). The variables $a,b,c$ are parameters, and t is the age. In this example we show three parameters $\left\{ {a,b,c} \right\}$, but the curves used here have number of parameters $p$ with $p = 2,3$.

The OLS equations for the conventional approach to modeling dinosaur growth used in previous studies (*e.g*., Cooper et al., 2008; Lee & Werning, 2008; Woodward et al., 2015) are shown in Eq. (3). The ages ${t_{i,j}}$ are assumed to be resolved into numerical values from the estimation of start ages $star{t_j}$ as an initial step. The OLS function $ols\left( {a,b,c} \right)$ is then minimized numerically using an algorithm such as Levenberg-Marquardt or Newton’s method (Press, 2007; Aster, Borchers & Thurber, 2018). These and similar local optimization algorithms function best when given a reasonable starting set of parameters $\left\{ {{a_0},{b_0},{c_0}} \right\}$, which are usually determined through approximate curve fitting approaches (Bates & Watts, 1988; Seber & Wild, 2003; Nocedal & Wright, 2006). With ${N_j}\;$as the number of age, circumference pairs in growth series $j$, the fitting procedure is:

(3) $$\eqalign{ ols\left( {a,b,c} \right) & = \mathop \sum \limits_{j = 1}^M \mathop \sum \limits_{i = 1}^{{N_j}} \left( {f(a,b,c,{t_{i,j}}} \right) - \; cir{c_{i,j}}{)^2},\quad {t_{i,j}} = cgm{c_{i,j}} + firs{t_j} \\\left\{ {a,b,c} \right\} & = \mathop {{\rm argmin}}\limits_{a,b,c} ols\left( {a,b,c} \right).}$$

In this study we use a novel modification of this approach, shown in Eq. (4), in which $starts$ are treated as independent variables and estimated simultaneously with curve parameters.

(4) $$\matrix{ {ols\left( {a,b,c,first} \right) = \mathop \sum \limits_{j = 1}^M \mathop \sum \limits_{i = 1}^{{N_j}} \left( {f(a,b,c,cgm{c_{i,j}} + firs{t_j}} \right) - \; cir{c_{i,j}}{)^2}\; } \cr {\left\{ {a,b,c,first} \right\} = \mathop {{\rm argmin}}\limits_{a,b,c}\; ols\left( {a,b,c,first} \right)} \cr }.$$

The total number of variables in the OLS function in Eq. (4) is thus $p + M - 1$, where $p$ is the number of parameters in the sigmoid. In the case of the *Tyrannosaurus rex* species complex growth series shown in Figs. 4 and 5, we have $M = 13$ series, and thus the sigmoid models fitted with Eq. (4) will have between 15 and 17 variables. However, the 13 series have ${N_j}$ with between 6 and 25 CGM, for a total of 198 data points. Since the number of data points greatly exceeds the number of variables, this is well within the capabilities of optimization software to find a solution. This condition remains true so long as the lowest value of ${N_j}$ is 2 CGM. The growth modeling approach presented here minimizes the sum of least squares between the modeled circumference, and the actual circumference as in Eqs. (3), (4).

In the terminology of statistics, parameters which must be estimated but do not appear in the primary result are known as “nuisance parameters” (Linnik, 2008; Casella & Berger, 2024). In constructing the *Tyrannosaurus rex* species complex population growth curve, the relative ages of the specimens are considered nuisance parameters, and our treatment of them here matches normal methods used in statistics. For a study focused primarily on relative ages, the curve parameters would instead be nuisance, and the relative ages would be central.

Fitting the curve using the extra parameters has slightly more uncertainty than without. The impact can be roughly gauged using the corrected Akaike information criterion, AICc.

$AICc\; = \; 2k\; - \; 2ln\left( {\hat L} \right)\; + \; \displaystyle{{2{k^2} + 2k} \over {n - k - 1}}$

where $n$ is the number of data points, $\; k$ is the number of parameters and $\; \hat L$ is the likelihood. If this is evaluated for $n = 152,\; \; k\; = \; 3$ *vs*. $k\; = \; 3 + 9\; = \; 12$, the difference in $AICc$ due to adding the nine relative age parameters is 20.1. For comparison, the term $- \; 2ln\left( {\; \hat L} \right)$ in the Trex2 cases here is typically ~1,300, so it is a small effect, about 1.5% of the AICc. Regardless of the cost in uncertainty, we have no alternative—all methods of estimating the relative ages (for example, through a separate “retrocalculation” estimate) would add to the parameter count $k$ in the same manner. The increase in AICc due to the extra relative age parameters thus plays no role in model selection as it applies equally to each model.

The argmin function in Eq. (3) is performed by an ordinary least squares nonlinear numerical optimization algorithm which begins with initial values for each parameter and then iteratively improves them. To begin the iteration, initial values (which are often called “guesses”) (Bates & Watts, 1988; Seber & Wild, 2003; Nocedal & Wright, 2006; Press, 2007) or each of the curve parameters and for the $first$ variables are obtained by using the least squares clustering algorithm, and a curve fit to the clustered result (Figs. 5, S10–S13). This gives an objective initial guess, and subsequent iterations of the optimization algorithm overcome any possible shortcomings in the initial least squares clustering.

Both theoretical and empirical studies show that some biological growth curves better model the growth of some organisms. There are multiple sigmoidal curves which can be used, each of which have different shapes, corresponding to differences in biologically interesting, derived parameters such as maximum growth rate, when during growth the point of maximum inflection and other factors occur (Tjørve & Tjørve, 2010; Goshu & Koya, 2013; Vrána et al., 2019). Some, like logistic, Gompertz, Richards and von Bertalanffy have long been used in growth rate studies for extant animals (Zullinger et al., 1984; Hernandez-Llamas & Ratkowsky, 2004; Tjørve & Tjørve, 2017a, 2017b), and for dinosaurs, starting with (Chinsamy, 1993).

Different curve families, such as logistic and Gompertz, have various parameterizations with differing numbers of parameters. Our study uses nine different sigmoidal growth curves (Table S3) for each variant to make it more likely that we use a fit which has a shape matching the growth. For example, the shape of the curve at the inflection point, where maximum growth is achieved, can be made symmetric (as with logistic) or not (Myhrvold, 2013). Another important factor is that some curves interact poorly with numerical fitting algorithms, because they can have mathematical singularities or become complex valued for certain parameter values. This is true, for example, for the Richards growth model, which contains many of the other models (logistic, Gompertz and von Bertalanffy) so it would seem ideal, and some authors make that argument (Tjørve & Tjørve, 2017b). But in actual practice numerical regression routines have great trouble with the Richards model so it seldom is chosen as the best fit.

Quadratic and linear non-sigmoidal curves were also applied because they can be useful diagnostics of the fitting process. Functions to be fit take the place of $f\left( {} \right)$ in Eq. (4), and the fitting procedure is repeated for each function as shown in Eq. (5). Each function, along with parameters from Eq. (4) and residuals of the fitting process, are termed the output.

For each variant, we then use the corrected Akaike information criterion AICc for model selection (Burnham & Anderson, 2002), by finding which of the $fi{t_k}$ has the minimum value of AICc.

(5) $$\eqalign{ fi{t_k} & = \left\{ {{f_k},{a_k},{b_k},{c_k},start{s_k}} \right\} \\ bestfit & = \mathop {{\rm argmin}}\limits_k \;AICc\left( {fi{t_k}} \right).}$$

The minimum value of the corrected AICc provides the best fitting model for use in analysis. However, it is also important to determine the statistical uncertainty for each of the best fit curves by estimating 95% simultaneous confidence bands (CB). In other words, the best fit growth curve should not be used for inference or drawing biological conclusions unless the uncertainty in the growth curve (expressed in the confidence band) is considered. While confidence intervals are about point estimates (*i.e*., scalar parameter value estimates) a simultaneous confidence band is the region in which (under the assumptions inherent in the regression) there is a stated confidence level (in our case, 95%) of finding the true curve which lies entirely within the band. Note that while confidence intervals are derived from the data, they are only interpreted with respect to the model fit. There is no importance attached to data points falling inside or outside the CB.

In addition to arithmetic fitting, logarithmic fitting was also performed by first transforming the circumference data, taking its natural logarithm, *i.e.*, $cir{c_i} \to {\rm ln}\left( {cir{c_i}} \right)$ for two reasons. The first was in case the datasets, like many growth data, are heteroskedastic, so that statistical errors in the model vary as a percentage of the circumference rather than as an absolute error. The second reason was to produce a more balanced distribution of errors; since least squares fitting minimizes the square of the difference between the model and the data, the large size (older) end of the growth curve typically dominates the fitting accuracy. After the logarithmic transformation the data can be fit as normally with similarly log-transformed sigmoidal models (Table S4).

### Evaluating compatibility of datasets

All 13 growth series for the *Tyrannosaurus rex* species complex (represented by either a tibia or femur for each individual and both femur and tibia for BMRP 2006.4.4) are included in dataset Trex1 A, plotted in Fig. 4. Visually, it is apparent that three of the CGM circumference growth series (Tibia BMRP 2002.4.1, Femur BMRP 2006.4.4 and Tibia BMRP 2006.4.4) are much longer than the adjacent series below or above them. The growth series in the Trex1 A dataset were next clustered as shown in Fig. 5 (other variants Figs. S12, S13), to provide a starting point for the growth model. The same anomalous specimens protrude from the bundle of series to the right, and one of them (Tibia BMRP 2006.4.4) also protrudes to the left.

**Figure 6:**
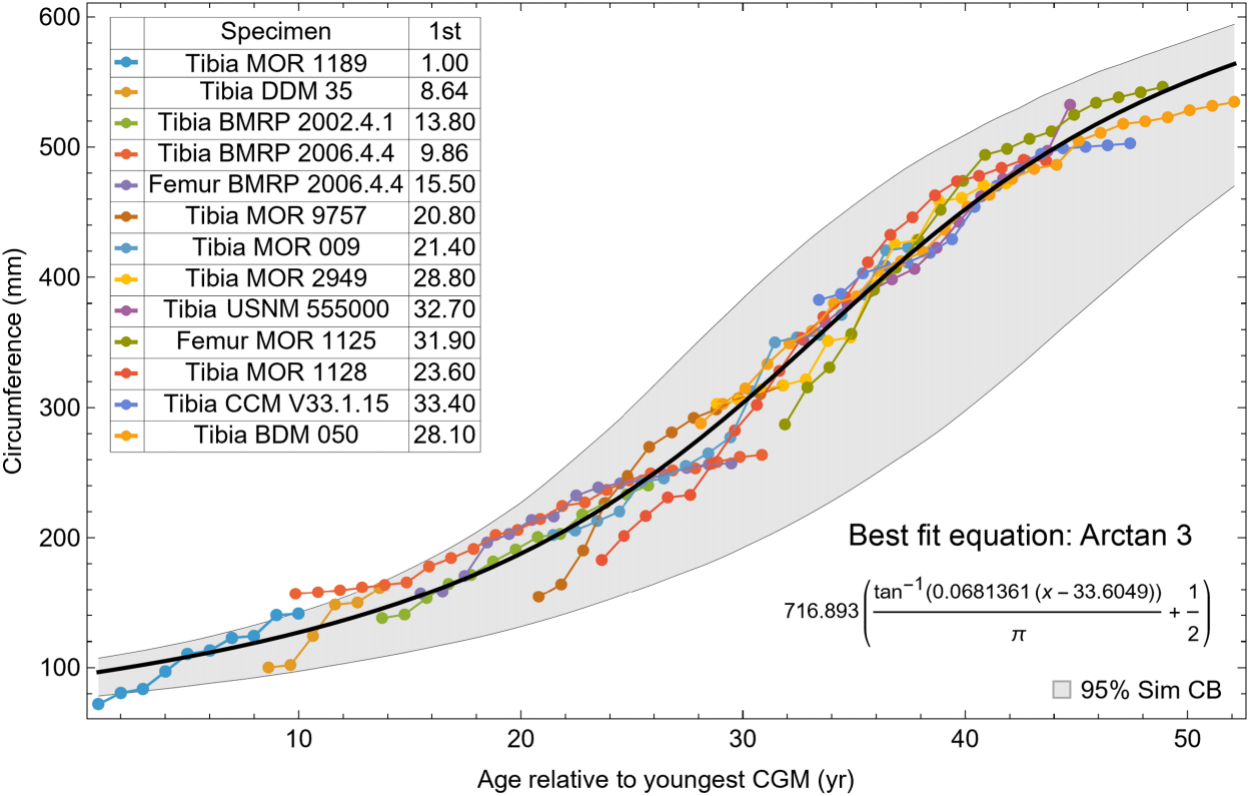
Growth series of dataset Trex1 (includes BMRP specimens), variant A, which incorporates all CGM. The best fit equation is shown, in this case the arctan 3 function. The age of 1 st CGM for each growth series is estimated with the method of Eq. (4). Problems with the fit are discussed in the text. The plots for variants NoX, NoM, and NoXM are qualitatively similar (see Figs. S14, S15).

The resulting growth model from the clustered growth series Trex1 A is shown in Fig. 6 with the other variations shown in Figs. S14, S15. 95% confidence bands (CB) were then assigned to each model variant. Since different fits have different lengths for their CB (because of variation in the starting ages for the CGM growth series), the best metric for evaluating the CB size is CB area divided by the age extent of the growth series. Each of the growth variants on the Trex1 dataset had wide 95% simultaneous confidence bands, possibly due to the anomalous three BMRP growth series.

To investigate, we proceeded with leave-one-out resampling (Arlot & Celisse, 2010; Qin et al., 2021). With 13 growth series, we systematically eliminated one series at a time from each Trex1 variant and then performed the model fit and confidence band calculation on the growth series for the remaining specimens. The result was that removing the femur and tibia of BMRP 2006.4.4 greatly reduced the CB area per length (Table 2).

**Table 2:** Confidence band (CB) areas per series length for the first round of leave-one-out tests using dataset Trex1. The Trex1 dataset variant A had one growth series removed (*i.e*., Trex 1A – 1 for removing series 1) then growth modeling done, sequentially for all growth series. The CB area per length is tallied for each dataset variant, and for the total across all datasets. The lowest CB areas occur by removing BMRP 2006.4.4 (in bold).

Growth series removed	Variant A	Variant NoM	Variant NoX	Variant NoXM	Total
Trex1–MOR 1189	120.1	124.8	125.8	125.7	496.4
Trex1–DDM 35	131.2	97.3	121.0	153.6	503.1
Trex1–BMRP 2002.4.1	135.8	95.9	88.9	157.6	478.2
**Trex1–BMRP 2006.4.4**	**45.8**	**72.0**	**98.7**	**149.7**	**366.2**
Trex1–MOR 9757	132.9	96.0	87.8	164.5	481.2
Trex1–MOR 009	140.9	103.2	95.5	117.6	457.2
Trex1–MOR 2949	135.6	98.1	134.7	110.0	478.2
Trex1–UNNM 555000	136.4	93.5	89.1	111.7	430.7
Trex1–MOR 1125	127.9	95.2	88.8	113.5	425.4
Trex1–MOR 1128	87.5	103.8	87.3	112.1	390.8
Trex1–CCM V33.1.15	142.9	97.8	86.4	103.8	430.9
Trex1–BDM 050	147.7	95.6	96.2	114.0	453.5

**Figure 7:**
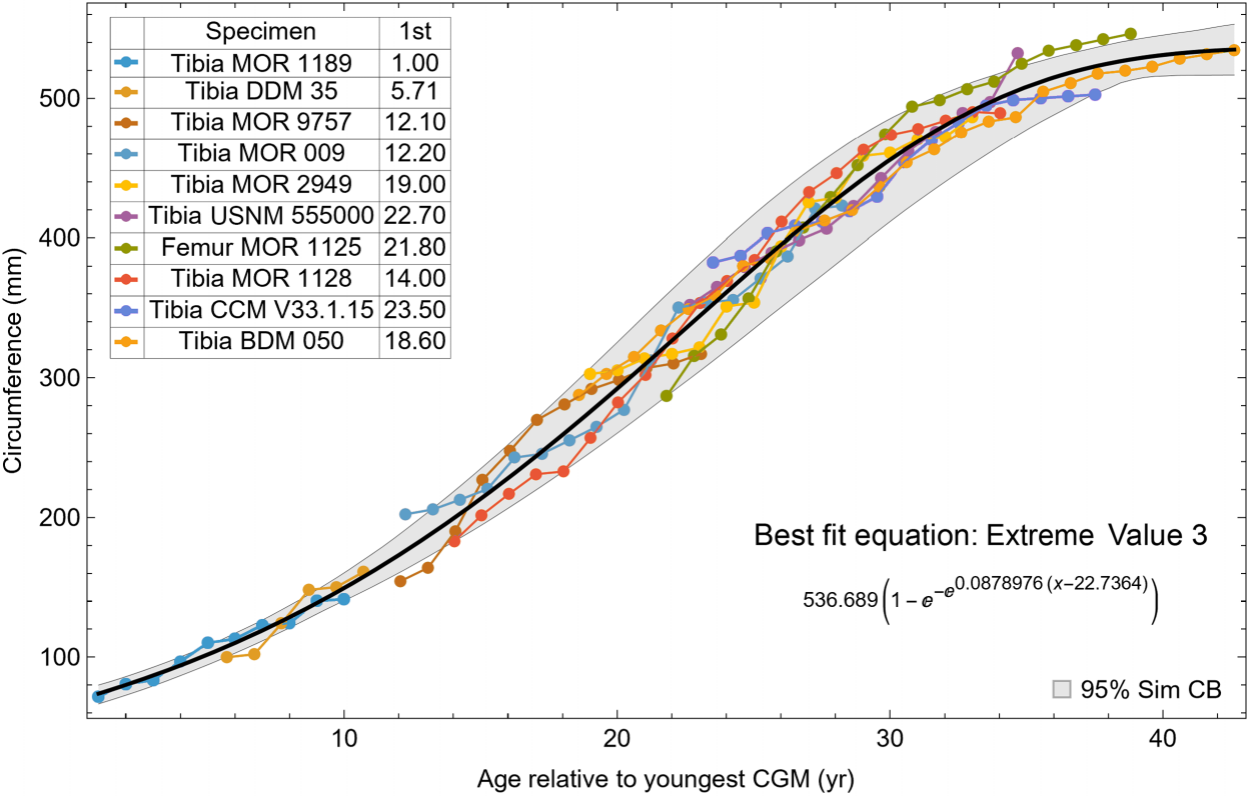
Best fit curve to the Trex2 dataset (BMRP specimens removed), A variant. The methods of Eqs. (4), (5) were used to find the best fitting curve (plotted in black). Each series is plotted in color with the starting ages determined at the same time as the sigmoid function parameters. The extreme value 3 sigmoid function is responsible for the best fit, and the equation for the curve is shown below the graph title. The shaded gray area is the 95% simultaneous CB for the fit determined *via* nonparametric bootstrap with 2,048 samples.

A second round of leave-one-out resampling showed that removing tibia BMRP 2002.4.1 further improved the model fits (Table 3). These results objectively validate the heuristic observation that these specimens seem out of place in Figs. 4, 5 and S10, S11. Four Trex2 dataset variants result from removal of the BMRP specimens, each comprised of 10 *Tyrannosaurus* growth series. The growth model for the Trex2 A dataset appears in Fig. 7 and other Trex2 dataset variants in Figs. S16, S17. With the removal of the BMRP specimens, the decrease in the CB area per length is substantial; 127.8 for Trex1, variant A, *vs*. 45.1 for Trex2, variant A. The total length of the CB is also shorter, 53 years for Trex1 A *vs*. 43 years for Trex2 A. While each curve represents a best fit to their respective datasets, the utility of the growth model for inference is greatly limited by the larger CB for Trex1.

**Table 3:** Confidence band (CB) areas per series length for the second round of leave-one-out tests using dataset Trex1. The second round of leave-one-out sequentially removed each of the remaining specimens and the lowest CB area occurred when BMRP 2002.4.1 was removed (in bold).

Growth series removed	Variant A	Variant NoM	Variant NoX	Variant NoXM	Total
Trex1–BMRP 2006.4.4, MOR 1189	55.3	115.5	98.0	114.2	383.1
Trex1–BMRP 2006.4.4, DDM 35	44.6	65.3	95.7	153.4	359.0
**Trex1–BMRP 2006.4.4, BMRP 2002.4.1**	**45.6**	**67.7**	**64.5**	**76.3**	**254.1**
Trex1–BMRP 2006.4.4, MOR 9757	47.5	145.0	99.8	142.8	435.0
Trex1–BMRP 2006.4.4, MOR 009	53.2	66.4	118.2	156.4	394.2
Trex1–BMRP 2006.4.4, MOR 2949	46.0	71.0	92.2	145.1	354.4
Trex1–BMRP 2006.4.4, UNNM 555000	46.9	64.5	95.5	145.9	352.9
Trex1–BMRP 2006.4.4, MOR 1125	40.8	60.0	104.1	160.6	365.5
Trex1–BMRP 2006.4.4, MOR 1128	52.7	84.6	106.3	167.1	410.7
Trex1–BMRP 2006.4.4, CCM V33.1.15	42.6	69.0	105.1	160.5	377.2
Trex1–BMRP 2006.4.4, BDM 050	68.8	91.3	106.2	142.9	409.2

The model fits in the Trex2 dataset are very good. The 95% quantiles of the fit residuals lie within −12.6% to +8.6% for variant A, and similar ranges for other variants (Fig. S18). In addition, when arithmetic and logarithmic plots are compared, the best fits lie within the 95% confidence band of each other (Figs. S19, S20). To further demonstrate the utility of confidence bands, Table 4 compares the difference in CB values for the two datasets and between each of their A and NoXM variants. For Trex1 variant A (see Fig. 6), the CB reaches its maximum high to low extent at the relative age 30.6 and with a high/low ratio of 2.02, at which point the best fit circumference is 312.5 mm. This circumference corresponds to a 6.97 CB ratio in mass. The Trex1 NoXM variant (Fig. S15) has a wider CB extent and thus larger high/low ratios; 2.37 for circumference and 10.72 for mass. This shows the problems inherent in using the Trex1 dataset: its variant A is only good to within a factor of 2 in circumference and 7 in mass in the worst case, while the NoXM variant is even more uncertain.

**Table 4:** Selected confidence band examples for Trex1 and Trex2 datasets. The first row reports the relative age at which confidence band extent (from upper boundary to lower boundary) is maximized on the best fit line in the A and NoXM variants for each dataset. Subsequent rows show the values of limb bone circumference and body mass (from conversion of circumference by Campione and Evans formula) corresponding to the properties of the CB at that point on the best fit line. Including multiplets and XPL-only annuli (variant A) produces smaller CB ratios than excluding these features (NoXM variant) for both Trex1 and Trex2 datasets, but the Trex1 dataset has larger CB ratios overall.

Quantity	Attribute	Trex1		Trex2	
		A	NoXM	A	NoXM
CB maximum (yr)	Relative age	30.6	18	22.3	13.9
Circumference (mm)	Best model fit	312.5	291.7	332	321.5
	CB low	197.3	166.1	292.5	261.8
	CB high	399.4	393.1	369.7	395.4
	High/low circ ratio	2.02	2.37	1.26	1.51
Mass (kg)	Best model fit	1,541.4	1,274.9	1,821.1	1,666.7
	CB low	434.5	270.4	1,284.5	946.7
	CB high	3,029.5	2,898.4	2,447.6	2,945.5
	High/low mass ratio	6.97	10.72	1.91	3.11

The Trex2 dataset has much smaller CB extents and thus smaller ratios. The A variant has a ratio in circumference of 1.26, which converts to a ratio of 1.91 in mass. There is still considerable uncertainty, but that is not surprising for a population. The NoXM variant again is much more uncertain, with a ratio of 1.51 in circumference and 3.11 in mass. These examples show why the uncertainty analysis is important, and why the Trex2 dataset and A variant provide a better basis for inference. The effects of specimen compatibility and of differences between variants are not small for these data.

**Figure 8:**
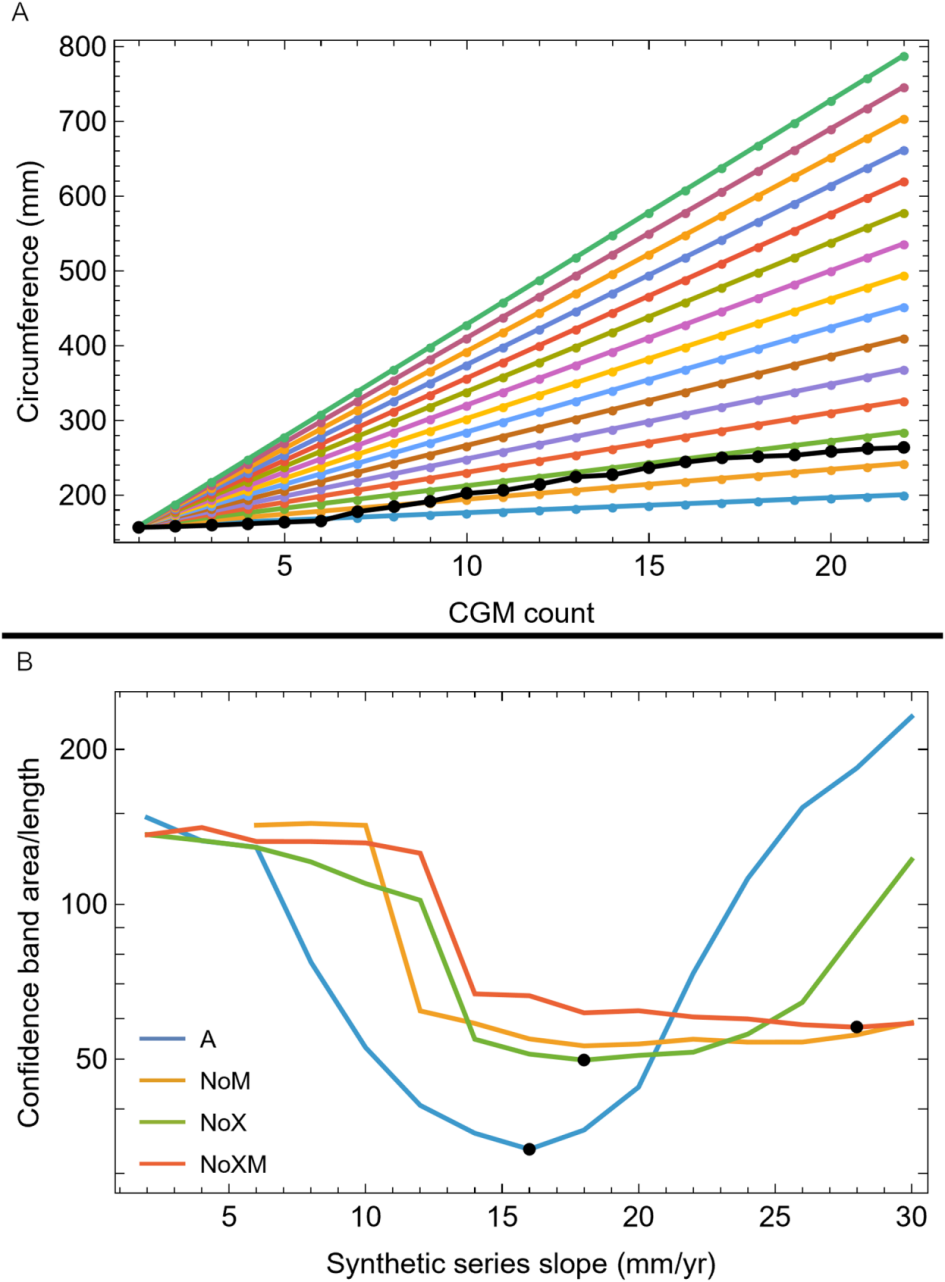
Synthetic growth series and test results for tibia BMRP 2006.4.4. (A) The actual, non-synthetic A variant (all CGM) growth series for tibia BMRP 2006.4.4 has 22 CGM in total (black series), and starts with a circumference of 158.5 mm. The colored series plot idealized linear test growth series that begin at the same circumference as the actual, non-synthetic BMRP 2006.4.4 tibia but then increase linearly with slopes between 2 mm/year for the slowest growth case, to 30 mm/year for the fastest growth case. Each synthetic series also has 22 CGM. Similar synthetic series were created for other variants—see Fig. S21. (B) The results of confidence band CB area/length (y-axis) for growth analysis on the synthetic series for each variant (colored curves) as a function the synthetic series growth rate (x-axis) using the Trex2 dataset. In each case the CB area/length reaches a minimum value denoted by a black dot. The minima occur at different values of the growth rate for each variant.

We then investigated which attributes of the removed growth series led to the greatly increased CB using a set of synthetic growth series that modeled growth as a straight line, as approximated by the BMRP 2006.4.4 tibia series. We kept the length the same as the BMRP 2006.4.4 tibia series for each variant and altered the slope from 2 mm/year increase in circumference to 30 mm/year circumference increase. Figure 8A shows these series for variant A, others in Fig. S18. The lower end of the growth rate series is a lower slope (on average) than the BMRP 2006.4.4 tibia series, while the higher end of the slope range is growing much faster than the tibia series.

Each of these synthetic growth series were added, one at a time, to the Trex2 dataset, and then the growth model was fitted and the 95% simultaneous CB estimated. The results in terms of CB area/length are plotted in Fig. 8B, and in terms of area in Fig. S19. The results of the synthetic growth series analysis show that the CB area and area/length reach a minimum (black points) for each variant. Those are the synthetic growth series and associated slopes that are most compatible with the Trex2 dataset in the sense that adding them would not dramatically increase the CB area or area/length.

**Figure 9:**
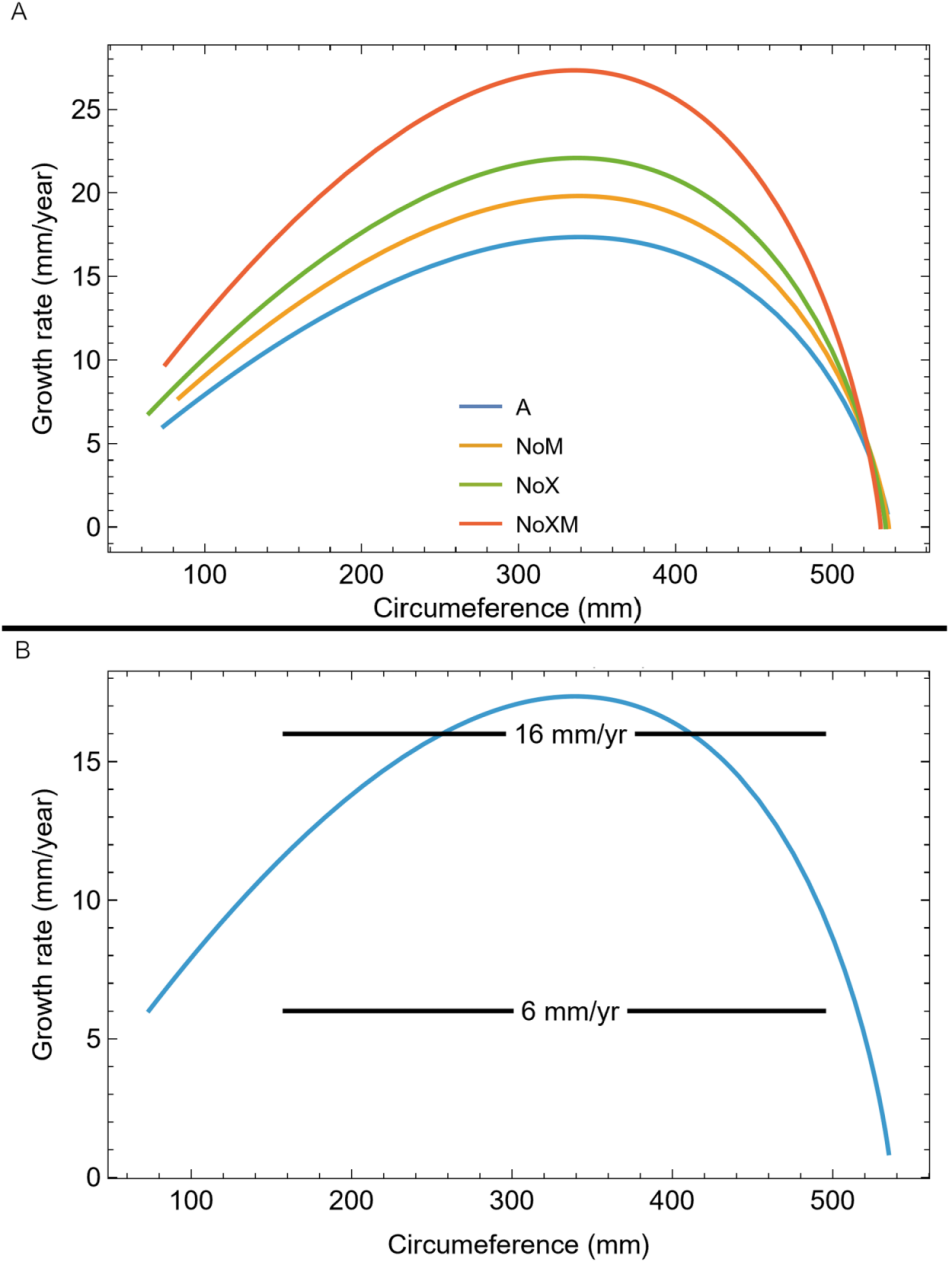
Growth rate as function of cortical growth mark (CGM) circumference for the Trex2 dataset best fit growth model variants. (A) Growth curves plot size (CGM circumference) *vs* time, but here size is plotted *vs* the growth rate (first derivative of size) for all four variants (colored lines). (B) The A variant of the best fit line to Trex2 (blue) and three synthetic growth series (black) are plotted for CGM circumference growth rates of 6, 14 and 16 mm/yr. The synthetic growth series have constant growth rates (slopes) which is the y-coordinate, over a range of circumference values (*i.e.*, a range in the x-coordinate).

We also plotted the growth rate (derivative of standard sigmoidal growth curve) *vs*. size (circumference) for all variants of Trex2 (Fig. 9A). In this format the synthetic growth series, having constant slopes, plot as horizontal lines (Fig. 9B). The synthetic series for 14 mm/year (minimum CB area/length) and 16 mm/year (minimum CB area) roughly approximate the curve. The synthetic series for 6 mm/year—which is approximately the average slope of tibia BMRP 2006.4.4—is far from the curve. It is also clear that synthetic series with growth rates in excess of 16 mm/year will also not lie near the curve.

Myhrvold (2013) introduced the concept of an anaptorythmic taxon, distinguished by having a different growth rate. We demonstrate that in the context of the growth series assigned to the *Tyrannosaurus rex* species complex, the omitted specimens BMRP 2006.4.4 and BMRP 2002.4.1 qualify as differing in growth rate from the other specimens in the Trex2 dataset but cannot be uniquely identified as comprising a separate taxon. This will be discussed further below.

### Evaluating model fit across variants

**Figure 10:**
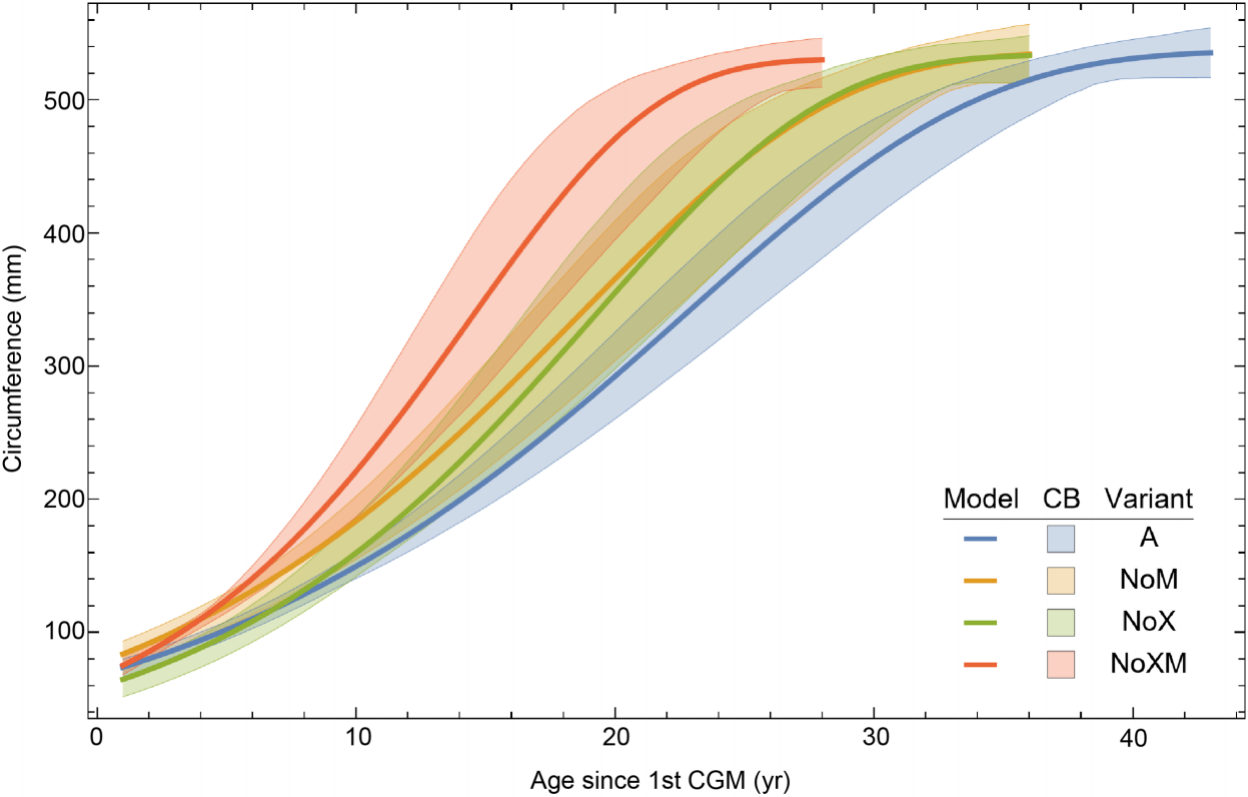
Growth models for each variant of the Trex2 dataset (excludes BMRP specimens) plotted together with their 95% simultaneous confidence bands CB. The best fitting models for each variant are shown as solid lines, while the 95% CB is shown as a shaded area.

Thus far in our study all four variations on *Tyrannosaurus rex* species complex growth (A, NoM, NoX, NoXM) have been modeled in parallel; we now turn to comparing them to see what advantages or disadvantages might accrue to each. The best fitting model for each Trex2 variant and their 95% simultaneous CB are plotted in Fig. 10. There is a hierarchy of growth rates because the variants cover approximately the same range in circumference but over different numbers of CGM. The NoXM variant (red) has the fewest points and thus the steepest curve (highest peak growth rate), and the most uncertainty (largest 95% simultaneous CB). The two middle variants, NoX and NoM are similar, and the CB almost completely overlap. This demonstrates the utility of the CB—since the NoX and NoM curves each lie within the CB of the other model, we can infer that the curves are not statistically distinguishable at the 95% level. However, the A and NoXM variants are distinct from each other, and from the NoX/NoM curves. Finally, the A variant has the least steep growth rate and smallest CB.

Assessing which variant best models the growth of the Trex2 dataset is complicated. Goodness of fit metrics, such as AICc (Burnham & Anderson, 2002) are generally only recommended for different models (*e.g*., logistic, Gompertz, von Bertalanffy) applied to the same dataset. In contrast, here the same model is applied to four different variations of the Trex2 dataset. For example, the Trex2 A variant has 152 data points in total while the NoXM variant has only 105. More generally, let the set $A$ represent the A variant data points for a given dataset, and $NoM$, $NoX$, $NoXM$ represent the respective sets for their variants. In that case the sets obey the following equations.

(6) $$\matrix{ {A = NoM\; \cup NoX = X \cup M\; \cup NoXM}\hfill \cr {NoM = X\; \cup NoXM}\hfill \cr {NoX = M\; \cup NoXM}\hfill \cr }.$$

Thus, the growth series in the $NoXM$ set also appears in all the other variants. The A set is the superset, the union of all the other variant sets. As stated above, in the case of Trex2 dataset, $A$ has 152 CGM points and NoXM has 105 CGM points. Excluded from NoXM are the set $X\;$ of XPL CGM with 22 CGM data points and the set M, due to multiplet CGM, with 27 data points.

Comparing AICc values for growth model fits to each of these sets, using arithmetic (*i.e*., not log-transformed) data, we find the values shown in line 1 of Table 5. Each value represents the best fitting (lowest AICc) model across all the models attempted. The resulting ΔAICc values cannot find the best model, however, because each has a different number of data points, giving the variant with the lowest data point count an unfair advantage with respect to sum-of-squared residuals.

**Table 5:** Comparison metrics between Trex dataset growth models from different variants. Each line in the table represents a different statistical comparison explained in the text. For each comparison metric, bold indicates the cell having the smallest value between the four variants.

Line number	Metric	Data	Point count	Fit	Variant A	Variant NoM	Variant NoX	Variant NoXM
1	Overall AICc	Trex2	152-105	Arith	1,258.40	1,086.53	1,173.61	**966.48**
2	Overall ΔAICc	Trex2	152-105	Arith	291.92	120.05	207.13	**0.00**
3	Overall AICc	Trex2	152-105	Log	**−482.38**	−378.47	−314.34	−242.49
4	Overall ΔAICc	Trex2	152-105	Log	**0.00**	103.91	168.04	239.89
5	Same pts AICc	NoXM	105	Arith	**889.19**	907.18	954.93	966.48
6	Same pts ΔAICc	NoXM	105	Arith	**0.00**	18.00	65.75	77.29
7	Same pts AICc	NoXM	105	Log	**−319.95**	−307.71	−248.08	−242.49
8	Same pts ΔAICc	NoXM	105	Log	**0.00**	12.24	71.87	77.46
9	Same pts AICc	NoM	127	Arith	**1,060.12**	1,086.53		
10	Same pts ΔAICc	NoM	127	Arith	**0.00**	26.40		
11	Same pts AICc	NoM	127	Log	**−401.04**	−378.47		
12	Same pts ΔAICc	NoM	127	Log	**0.00**	22.57		
13	Same pts AICc	NoX	130	Arith	**1,087.43**		1,173.61	
14	Same pts ΔAICc	NoX	130	Arith	**0.00**		86.18	
15	Same pts AICc	NoX	130	Log	**−401.72**		−314.34	
16	Same pts ΔAICc	NoX	130	Log	**0.00**		87.38	
17	CB area	Trex2	152-105	Arith	**1,913.64**	2,329.80	2,325.08	1,992.75
18	CB area	Trex2	152-105	Log	**5.75**	5.70	7.78	6.51
19	CB area/length	Trex2	152-105	Arith	**45.56**	66.57	66.43	73.81
20	CB area/length	Trex2	152-105	Log	**0.14**	0.17	0.22	0.24

To illustrate this, line 2 of Table 5 computes ΔAICc, with the expected result that the lowest AICc occurs for the variant with the smallest number of datapoints, which is NoXM. However, when using growth models fit to log-transformed data (line 3, 4 of Table 5), the smallest AICc now belongs to the A variant despite the disadvantage of more datapoints.

Since AICc is not normally applied to datasets with different numbers of points, we now compute only AICc values for the data points shared across all variants (“same-points AICc”). In Table 5, line 5 computes AICc for the NoXM subset of 105 CGM data points which appear in each of the variants and line 6 finds the ΔAICc values, which show that the A variant is the best model. This is a remarkable finding—even though the growth model fit to the A variant has roughly 50% more data points, it fits the NoXM points better than a model fit to those points alone. Lines 7, 8 in Table 5 show that the A variant is also the best model fit to log-transformed points.

In Table 5 lines 9–12 repeat the same-points AICc procedure but now consider the 127 points from the NoM variant with the 152 points in the A variant. Again, variant A has the lowest same-points AICc score. Lines 13–16 in Table 5 do same-points AICc based on the 130 points in NoX which are shared with variant A, with the same result. Thus, from an AICc model selection standpoint, variant A is always to be preferred for the Trex2 dataset, because it obeys the following conditions.

(7) $$\matrix{ {\Delta {\rm AICc}\left( {A,NoXM} \right) = 0,\; 0 < \; \Delta {\rm AICc}\left( {NoM,NoXM} \right) < \Delta {\rm AICc}\left( {NoX,NoXM} \right) < \Delta {\rm AICc}\left( {NoXM} \right)}\hfill \cr\;\; {\Delta {\rm AICc}\left( {A,NoM} \right) = 0,\; \; 0 < \Delta {\rm AICc}\left( {A,NoM} \right)}\hfill \cr\;\;\; {\Delta {\rm AICc}\left( {A,NoX} \right) = 0,\; \; 0 < \Delta {\rm AICc}\left( {A,NoX} \right).} \hfill\cr }$$

In Eq. (7), the notation $\Delta {\rm AICc}\left( {A,NoXM} \right)$ is the same-points AICc, *i.e*., the delta AICc value for the best fit model for CGM series variant $A$ computed using only the points common to $A$ and $NoXM$. Line 17 and 18 in Table 5 show a different metric, the total area of the 95% simultaneous confidence band, and lines 19, 20 the area divided by the length of the CB which shows that variant A has the smallest confidence band.

If XPL or multiplet CGM were truly extraneous or “supernumerary”, then one would not expect variant A to be the best fit to the dataset and to have the smallest confidence band; extra, fallacious, data points should hinder, not improve, the model fits when scoped to the same points. The NoXM variant has errors minimized only on the basis of its points. Meanwhile, the A variant has ~50% additional points, each of which contributes to the sum of squared residuals, which is the basis for OLS regression and also the basis for estimating the log-likelihood which is the main component of AICc. The extra data points apparently influence both the starting ages and parameter values to achieve a fit which has lower AICc despite having more contributions to the sum of squared residuals.

We further tested the null hypothesis, that the XPL or multiplet CGM are extraneous or illusory points. This was done with Monte Carlo simulations creating genuinely extraneous points which randomly fall within the range of the growth series. Random sets $X$ and $M$ of fictitious CGM representing XPL or multiplet CGM respectively were generated under the assumption that they occur with uniform random placement within the range of the NoXM points. Then a full dataset was constructed with them, plus the NoXM CGM following Eq. (6). These were then fit with a growth model as with Eq. (5), and the same-points AICc calculated for the cases covered in Table 5. Out of 10,000 Monte Carlo trials, only 68 matched the conditions of Eq. (7). The null hypothesis that the XPL or multiplet CGM are random “extra” lines not relevant to the growth series is thus rejected with $P = 0.0068$.

These results, and those in Table 5 provide strong statistical evidence that both XPL and multiplet CGM contribute to growth modeling the Trex2 dataset. Even if one argues that the only valid CGM are those in the NoXM set, the best fit to that set (in terms of least squares or AICc) occurs when models based on the A variant (which includes XPL and multiplet CGM) are used for the regression.

## Discussion

### Qualitative osteohistology

#### Primary and secondary tissue organization

**Figure 11:**
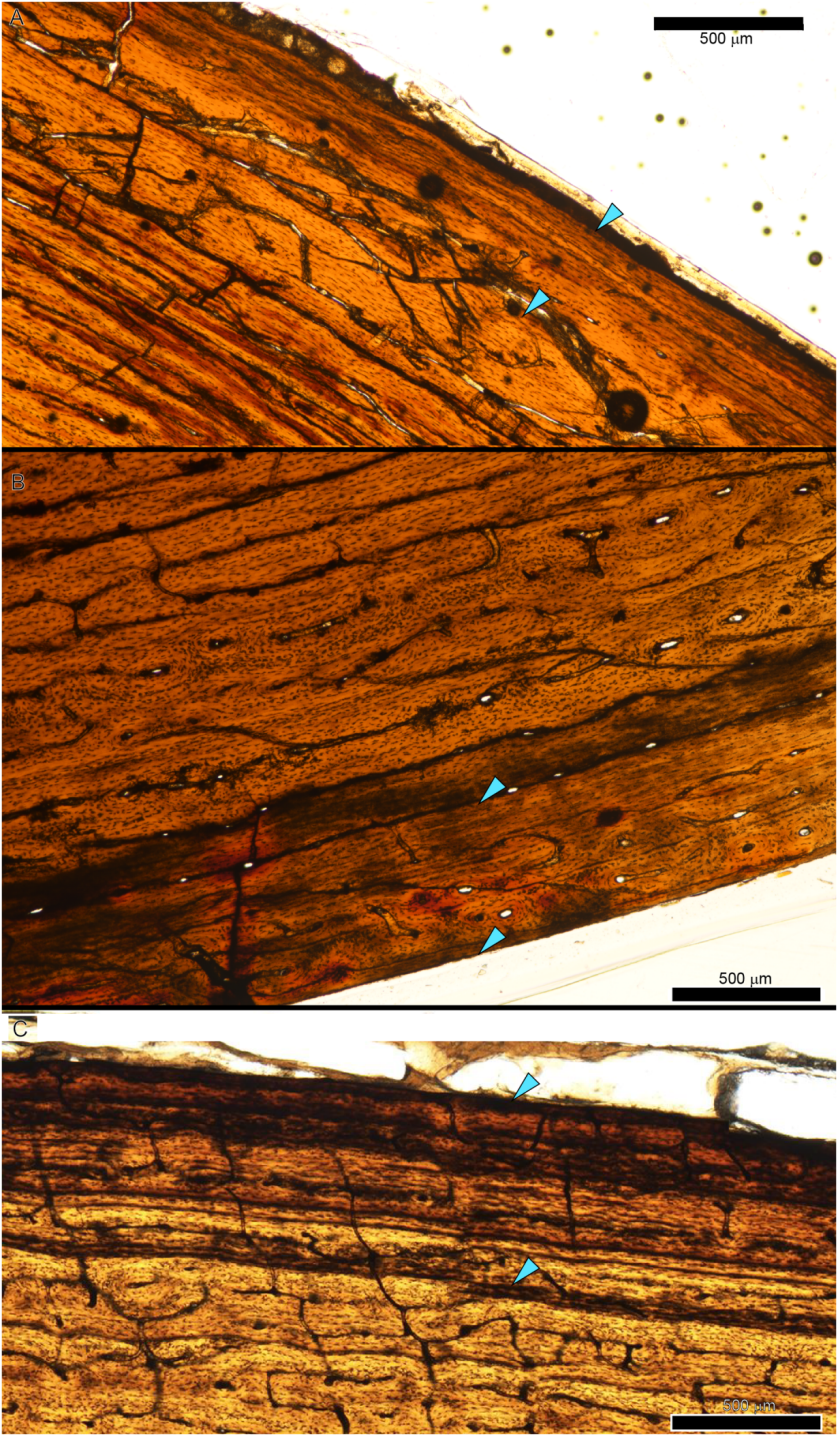
The external fundamental system (EFS), signaling skeletal maturity, was found in three tyrannosaur specimens from this study. For each panel, the region of the EFS is bounded by arrows. (A) *Tyrannosaurus* tibia CCM V33.1.15. (B) *Tyrannosaurus* tibia BDM 050. (C) *Gorgosaurus libratus* tibia TMP 1994.012.0602.

In extant vertebrates, relative annual growth rate is generally highest when young and decreases approaching asymptotic size (de Buffrénil, Quilhac & Castanet, 2021). This growth pattern is reflected qualitatively by successively thinner zones of increasingly organized collagen fibers and vascularity from inner to outer cortex (Amprino, 1947; Castanet et al., 1996, 2000; de Margerie, Cubo & Castanet, 2002; de Margerie et al., 2004). In contrast, our datasets show that from early juvenile to late sub adult, the femur and tibia cortex of *Tyrannosaurus* and *Gorgosaurus* was generally a plexiform to laminar woven-parallel complex, corresponding to active appositional growth between 25 and 100 microns/day (de Margerie, Cubo & Castanet, 2002; de Buffrénil, Quilhac & Cubo, 2021). More slowly formed parallel-fibered and avascular tissue was only consistently observed in the outermost cortex prior to the lamellar EFS in two of the largest *Tyrannosaurus* specimens, CCM V33.1.15 and BDM 050, and the larger of the two *Gorgosaurus*, TMP 1994.012.0602 (Fig. 11).

**Figure 12:**
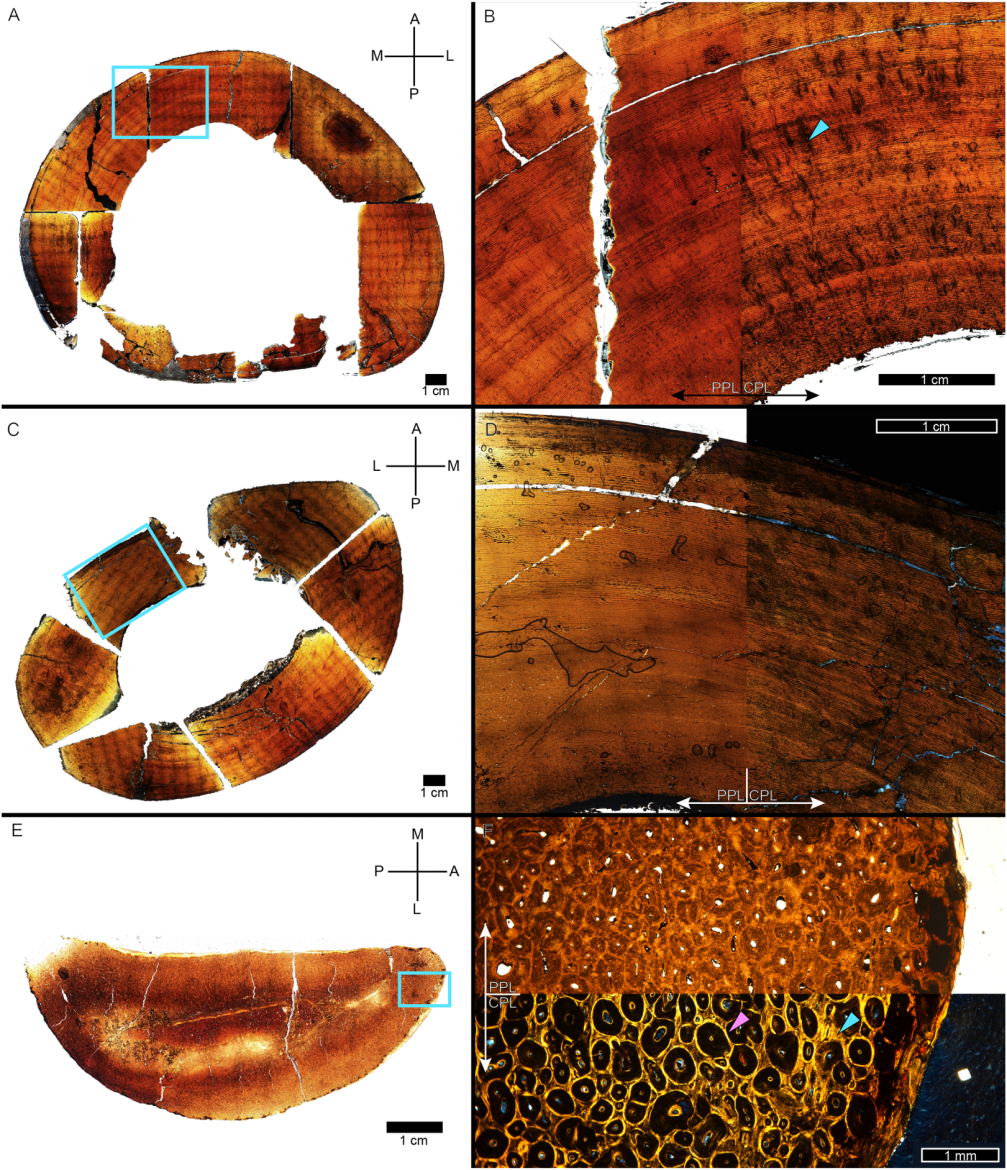
BDM 050 left femur, tibia, and fibula osteohistology. (A) Femur, imaged in PPL. Box indicates the region of interest in panel B. (B) Anteromedial region of femur cortex. The left half of the image is in PPL and the right half in CPL. Cortex is a plexiform to laminar woven parallel complex throughout, with frequent radial anastomoses (arrow). Dense Haversian remodeling is only present within the innermost cortex near the medullary cavity. (C) Tibia, imaged in PPL. Box indicates the region of interest in panel D. (D) Anterolateral region of tibia cortex, with the left half of the image in PPL and the right half in CPL. Cortex is a plexiform to laminar woven parallel complex throughout but lacks the frequent radial anastomoses observed in the femur. Dense Haversian remodeling is only present within the innermost cortex near the medullary cavity. (E) Fibula, imaged in PPL. The fibula is formed of compact bone from medullary region to outermost cortex. Box indicates the region of interest in panel F. (F) Anterior region of fibula, with the top half of the image in PPL and the bottom half in CPL. Cortex is almost completely comprised of dense Haversian tissue, made particularly evident by birefringent cement lines and isotropic lamellae in CPL (example, purple arrow). Interstitial primary cortex, when present, is avascular and lamellar (example, arrow). CGM, cortical growth mark; PPL, plane polarized light; CPL, circularly polarized light. Cross section orientations: A, anterior; M, medial; P, posterior; L, lateral.

Most specimens in this study were represented by a tibia and one of them by a femur (MOR 1125). We were able to qualitatively examine the bone tissue of both femur and tibia in the cases of BMRP 2002.4.1, BMRP 2006.4.4, and MOR 9757, and that of the ipsilateral femur, tibia, and fibula for *Tyrannosaurus* BDM 050. As tyrannosaur fibulae have been used to make life history inferences in previous studies (*e.g*., Erickson et al., 2004, 2006, 2010; Cullen et al., 2020; Persons, Currie & Erickson, 2020), the intraskeletal sample from BDM 050 provided an opportunity to assess the utility of fibula histology, albeit in a skeletally mature individual (Fig. 12). The fibula of BDM 050 does not display the primary woven-parallel complex observed in its weight-bearing femur and tibia (Figs. 12A–12D). Instead, the fibula cortex (Figs. 12E, 12F) was almost entirely formed of numerous secondary osteon generations, and the avascular lamellar periosteal surface was rarely visible interstitially. Thus, compared to its femur and tibia, the fibula of BDM 050 was uninformative for skeletochronology or assessments of annual growth rate due to its much higher rate of remodeling.

**Figure 13:**
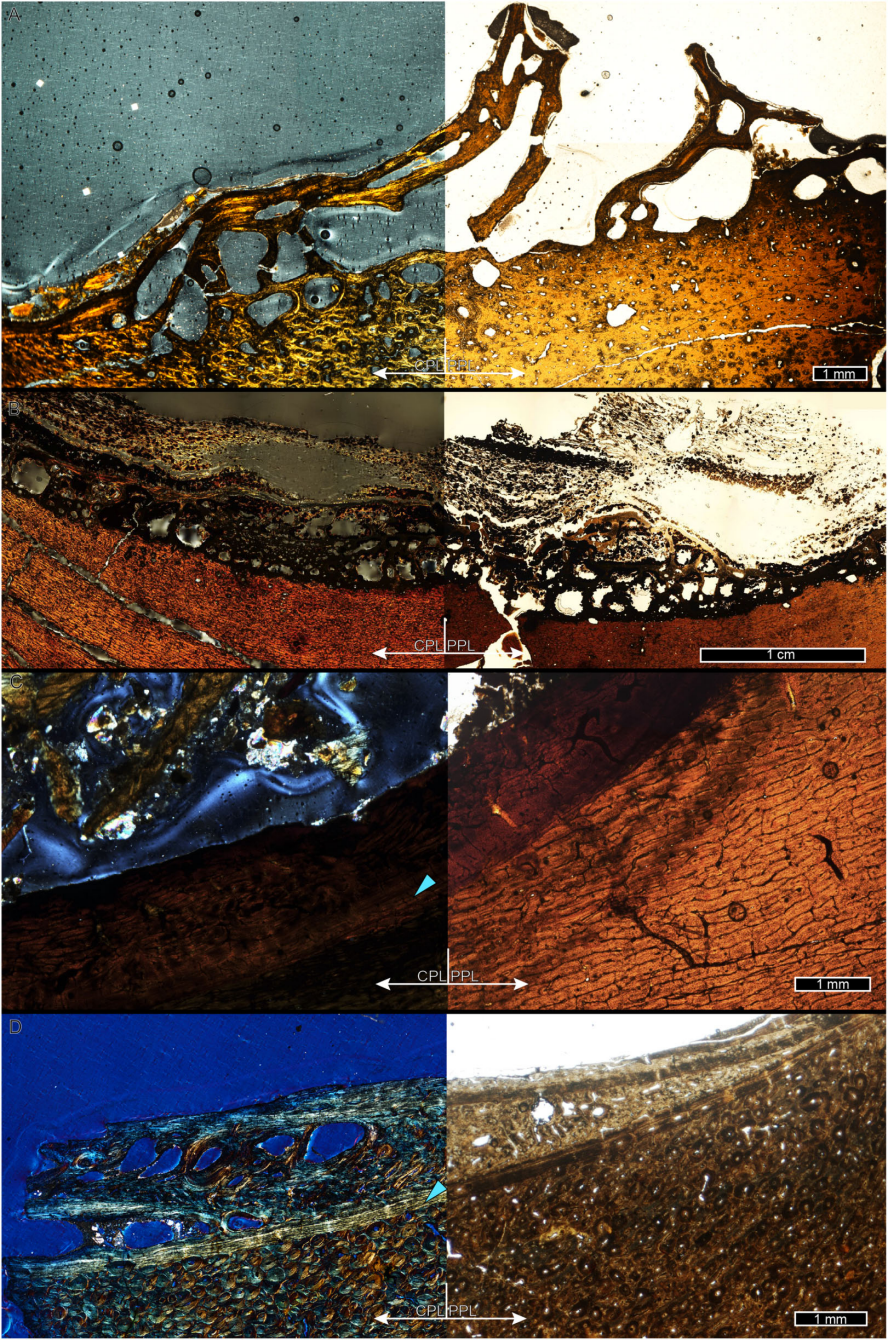
Localized, atypical cortical bone morphology bounds the medullary cavity in some *Tyrannosaurus* tibiae. Panels are imaged in CPL on the left and PPL on the right. (A) A region of the innermost cortex and lamellar endosteal layer of CCM V33.1.15 and (B) BDM 050 was remodeled to coarse cancellous bone prior to death. (C) A region of well-vascularized woven-parallel primary tissue formed on the surface of the lamellar endosteal layer (arrow) in MOR 2949 and (D) MOR 1198. Atypical cortical tissues were also observed in MOR 1125 and BMRP 2006.4.4 but are not figured as they are currently under study. PPL, plane polarized light; CPL, circularly polarized light.

Lastly, six individuals (BMRP 2006.4.4, MOR 1125, MOR 2949, MOR 1198, CCM V33.1.15, and BDM 050) possessed unusual compact to cancellous remodeling adjacent to the medullary cavity or endosteally derived bone growing into the medullary cavity. The endosteal tissue of BMRP 2006.4.4 and MOR 1125 are currently under study, but images of the endosteal tissues in the remainder of the specimens are shown in Fig. 13. Exploration of the mechanisms behind the formation of these endosteal tissues is beyond the scope of this project, but we note their presence should there be scientific interest in exploring its occurrence in future studies.

#### Double LAG and multiplets

Our ontogenetic dataset confirms previous observations that zonal spacing in *Tyrannosaurus* femora and tibiae varies from inner to outer cortex (*e.g*., Cullen et al., 2020; Woodward et al., 2020), rather than predictively decreasing in thickness approaching the growth asymptote. Here we extend this observation to include *Gorgosaurus*. We identified zones of irregular thickness in early to late-ontogeny as groupings of one or more consecutive zones of woven-parallel complex bounded by relatively thicker zones to either side of the group. In extreme cases, the relatively narrower zones were as thin as one lamina. This variable zonal spacing can result from double LAG or multiplets.

Double LAG are described in the literature as two or more consecutive CGM that merge into fewer lines one or more times over their circumferences and are often considered together as representing a single hiatus event (*e.g*., Castanet & Baez, 1988; García-Martínez et al., 2011; Nacarino-Meneses, Jordana & Köhler, 2016; Pereyra, 2023). While we did identify instances of double LAG in several individuals (*e.g*., Fig. 3, Table S1), inconsistency in zonal spacing within our dataset was primarily due to multiplets. Note that in this study double LAG are not considered multiplets but are treated as a single CGM. Although Cullen et al. (2020, 2021) explicitly link double LAG and multiplets, the latter do in fact differ from double LAG in that the CGM remain parallel to one another over their entire circumferences. In extant lissamphibians such as salamanders and toads, annually repeated multiplets, or ‘supernumerary lines’, consist of a relatively thick zone of apposition followed by two closely spaced CGM (Caetano & Castanet, 1993; Castanet et al., 1993, and references therein). Specifically, in the case of alpine salamanders, supernumerary CGM result from two annually occurring stressful events- estivation followed later the same year by hibernation (Caetano & Castanet, 1993). Multiplets are also known to occur at irregular intervals and represent individual annual events. A study validating the annual periodicity of LAG in known-age juvenile loggerhead and Kemp’s ridley sea turtles found that counting each CGM within a multiplet as a single year was the “correct interpretation” of their data, and the variable LAG spacing resulted from a year of “extreme decreased growth” (Snover & Hohn, 2004). Similarly, a study on the water frog *Pelophylax* interpreted close LAG spacing as resulting from poor growth due to environmental stresses (Socha & Ogielska, 2010).

Multiplets have been noted in earlier studies of *Tyrannosaurus* and in other non-avialan theropods (*e.g*., Lee & O’Connor, 2013; Cullen et al., 2014; Zanno et al., 2019; Woodward et al., 2020; Funston & Currie, 2021; Jevnikar, 2022; Jurašeková et al., 2022; Krumenacker, Zanno & Sues, 2022; D’Emic et al., 2023) as well as in some ornithischians (Cullen et al., 2014). Extant studies are often used to support a supernumerary interpretation for multiplets so that they may be considered as annual groupings for the purposes of dinosaur skeletochronology and growth curve modeling. This interpretation (either explicitly or implicitly depending on the study) holds that the multiplets occur over the course of a single “bad year” in which growth stops and starts multiple times, which is the justification for ignoring all but one of the parallel CGM in the sequence.

However, this common treatment of multiplets in dinosaur skeletochronology is problematic for several reasons. First, multiplet assignment is implicitly based on the subjective assessment that the zone width is abnormally thin for its position in the cortical growth series. For example, closely spaced LAG or annuli at the periosteal surface are generally not considered a multiplet and instead categorized as an EFS, while closely spaced LAG or annuli that are bordered by zones which are noticeably wider are likely to be classified as a multiplet.

In effect this is a subjective way of judging which zones appear to be out of keeping with sigmoidal growth. On the other hand, Cullen et al. (2020), p. 11 Supplemental Information) do provide clear criteria for multiplet identification:

“a ‘multiple growth mark’ can frequently be distinguished from multiple distinct growth marks by tracing the marks around their circumference on a full transverse thin-section (17). In most cases, double/multiple growth marks will merge and diverge multiple times over the circumference, whereas a LAG will remain distinct over that circumference.”

However, these criteria are previously used in the literature to define the terms ‘split’ or ‘double LAG’. What is more, most of the LAG and annuli observed in our study do not split or merge but instead are distinct CGM—it is only the adjacent zonal spacing that causes them to be classified as multiplets. Cullen et al. (2020, p. 12 Supplemental Information) apply their criteria to one specimen (BMRP 2006.4.4) which is featured in the present study:

“we have reinterpreted multiple individual LAGs (as described in (20)) to instead be double or multiple growth marks, largely based on examinations of the full sections of the femur and tibia, and tracing the occurrence and splitting/merging of some of these marks across their distribution (Fig. 2C).”

**Figure 14:**
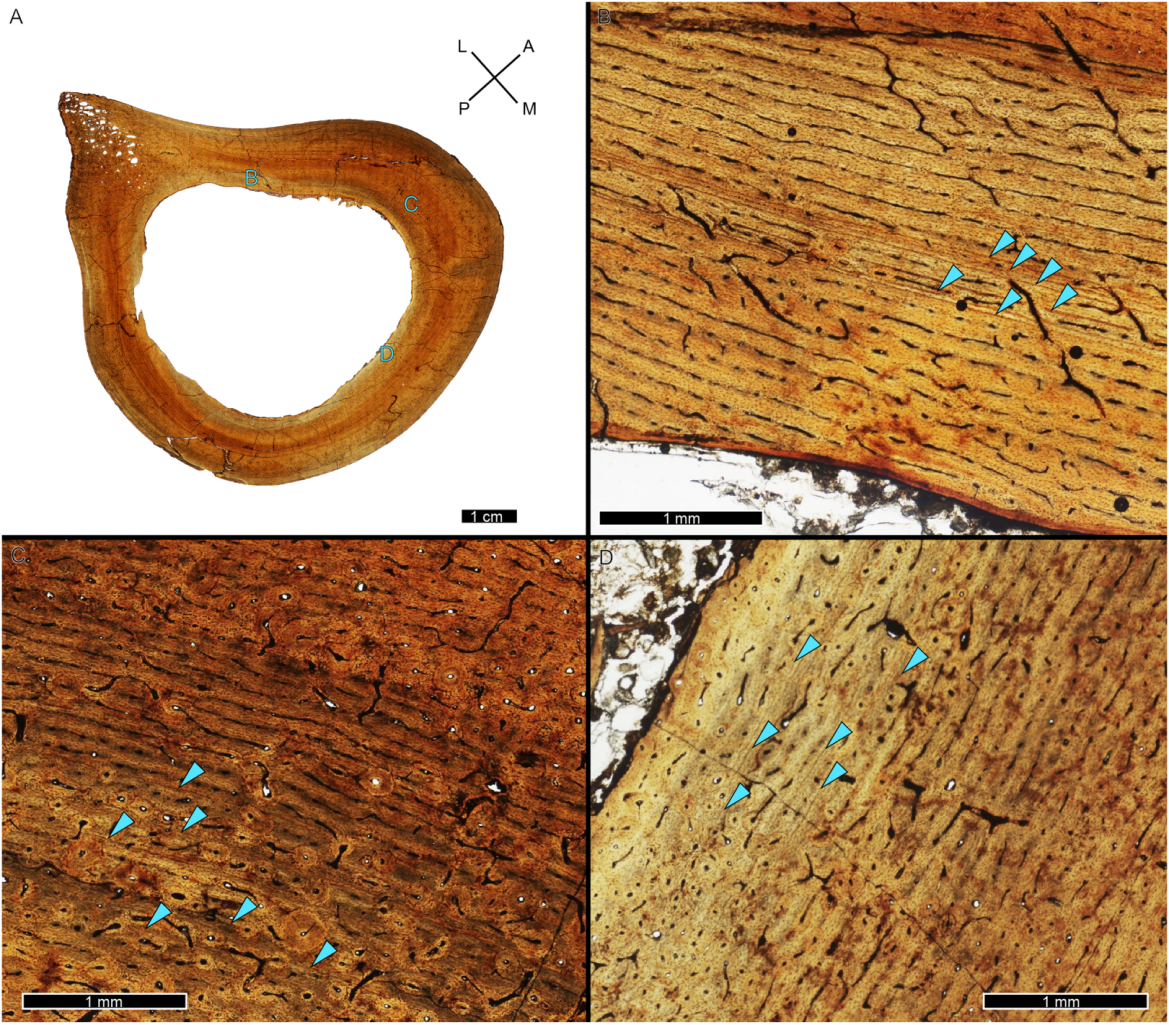
Six CGM within the inner cortex comprise a multiplet in the tibia of BMRP 2006.4.4, distinguished from a “double LAG” because the lines do not merge and split but instead remain parallel to one another about the cortex. (A) Tibia transverse section. Blue letters indicate the regions of interest in the associated panels. (B) Anterolateral region of tibia cortex. Six CGM (arrows) span the inner- to mid-cortex. Cortical apposition was relatively lower anterolaterally, causing CGM to be closely grouped compared to other regions. (C) Anterior tibial cortex. Here the same six CGM (arrows) observed in panel B are spaced further apart due to increased cortical drift. (D) Medullary drift results in the six CGM (arrows) being located within the inner cortex on the medial side. Medullary enlargement destroyed the record of these CGM on the posterior and lateral sides of the cortex. All panels in plane polarized light. CGM, cortical growth mark. Cross section orientations: A, anterior; M, medial; P, posterior; L, lateral.

This is an important distinction; Cullen et al. (2020) omitted the CGM they considered double LAG, thereby reducing the minimum age estimate for BMRP 2006.4.4 by half of that reported in Woodward et al. (2020). Reducing the number of CGM had the additional effect of increasing the slope (annual apposition rate) of the reported growth trajectory (Cullen et al., 2020). Yet our examination of the thin sections at high resolution reveal none of the splitting or merging reported in the quoted passage by Cullen et al. (2020), suggesting that while splitting/merging may seem like an objective criterion, in practice different investigators can draw opposing conclusions (Fig. 14). Furthermore, in BMRP 2006.4.4 this is not a small effect, changing the CGM count by a factor of two. Despite the subjectivity of multiplet identification up to now, we argue that a standard definition of multiplet should not be proposed until the cause(s) of sporadically occurring multiplets is better understood with empirical data and further investigations using extant vertebrates.

There are also several other logical difficulties with the treatment of multiplets. The supernumerary lines observed in the frequently cited extant examples (Senning, 1940; Castanet et al., 1993) are from tiny lissamphibians that live in very specialized environments and annually have two dormant periods (estivation and hibernation) which produce two LAG per year. Thus, the concomitant regular periodicity of their environment is reflected in CGM. Multiplets observed in dinosaurs differ from those observed in extant lissamphibians in that the features appear without any periodic pattern in the former, contra to the alpine salamanders and other taxa which regularly experience several growth seasons within a year. Despite the difference in presentation, this led to the assumption that multiplets are due to consecutive growth interruption events which could include adverse climatic conditions, resource scarcity, or pathology (disease, injury). While it seems likely that these factors could negatively influence bone growth and thus could be recorded in CGM, it is far from clear that this must occur within a single year. Scaling of metabolism and energetic reserves allow large body size organisms more ability to survive periods of resource interruption. It is unclear that growth would stop and resume as easily or as quickly in large dinosaurs as it would in tiny body size lissamphibians in which this phenomenon has been documented (Schmidt-Nielsen, 1997).

Instead of recording multiple periods of growth and recovery over a year, multiplet CGM could just as easily record multiple consecutive bad years and associated CGM, with less than normal growth (and thus thinner zones between CGM). That is the interpretation of multiplets adopted here in the A and NoX variants. Except in the context of BMRP 2006.4.4 (Woodward et al., 2020) this hypothesis has not, to our knowledge, been considered (or even mentioned) in previous dinosaur growth studies as an alternative explanation to the practice of assuming multiplets must occur within a single year (and thus discarding some multiplet lines).

Many previous studies attempt to fit growth curves to a CGM record derived from a single specimen and thus model the growth of one individual. In that context, including multiplet CGM seems contra to a growth model which predicts gradual changes in zone spacing with age. But in the context of pooled growth from numerous specimens the multiple CGM are revealed to have statistical value to the overall growth curve. Because we use many individuals to construct a growth curve for the *Tyrannosaurus rex* species complex, the statistical evidence shows that including multiplet CGM as annual records produces better growth models than treating multiplets as occurring over a single year. Conversely, adding random synthetic multiplet lines does not improve models. These are strong but indirect demonstrations that the multiplet lines have statistical value in these growth series. However, since the statistical evidence is based on the datasets studied here, the value of multiplets to modeling may not generalize to other species or taxa.

This evidence must be reconciled with a better histological understanding of multiplets. Until then, the more objective approach is to consider that individual dinosaur growth rates are plastic, as has been previously proposed (*e.g*., Sander & Klein, 2005; Woodward et al., 2015; Barta, Griffin & Norell, 2022) and as directly observed in the bone records of extant archosaurs—crocodilians and birds (*e.g*., Hutton, 1986; Jacobsen & Kushlan, 1989; Woodward, Horner & Farlow, 2014; Canoville, Chinsamy & Angst, 2022; Ong et al., 2022). In which case, each CGM in a growth series represents an annual hiatus (here, the A and NoX variant curves), irrespective of whether its spacing seems subjectively out of keeping with surrounding CGM. Because tissue organization remained constant over *Tyrannosaurus* and *Gorgosaurus* ontogeny, variable zonal thicknesses may instead partly reflect plasticity in annual hiatus duration (Lance, 1989; Dalrymple, 1996). In other words, narrow zones could result from an apposition rate nearer the 25 micron/day lower limit for the plexiform and laminar woven-parallel complexes (de Margerie, Cubo & Castanet, 2002; de Buffrénil, Quilhac & Cubo, 2021), coupled with a long growth hiatus.

#### XPL annuli

de Buffrénil & Quilhac (2021 p. 161) define an annulus as reflecting “a temporary decrease in growth rate, and many structural details in osseous matrices (as well as cells and vascular canals) can express this phenomenon. For example, when the type of matrix deposited during active growth phases is woven fibered, the annuli are then of either the parallel-fibered or the lamellar types. Similarly, if the “background matrix” is parallel fibered, the annuli will then consist of lamellar bone”. Thus, annuli are more slowly formed than the surrounding matrix and have relatively higher birefringence when viewed in cross polarized light (XPL). We used circularly polarized light, a modification of XPL (Bromage et al., 2003; see Materials & Methods) to confirm their presence and path about the cortex. We frequently observed diffuse birefringent bands in XPL but could not identify corresponding areas of reduced vascular density or flattened osteocyte lacunae in plane polarized light (PPL) (Fig. 2). Using the above definition of annuli from de Buffrénil & Quilhac (2021), we hypothesize such bands might result from a slight decrease in the apposition rate, forming somewhat more organized (birefringent) bone fibers relative to surrounding tissue but without the change in vascularity or osteocyte lacuna shape that would be visible in PPL. The annulus-like birefringent rings varied in thickness from a single lamina to more than 2 mm. Based on these observations, the XPL-only rings satisfy the description of an annulus in that they consist of fibers more organized (birefringent) than the surrounding matrix, associated with a brief decrease in apposition rate (de Buffrénil & Quilhac, 2021). They are biologically plausible since the inferred mechanism is essentially the same as conventional annuli, simply presenting with a different degree of organization which requires circularly polarized light to be visible. Therefore, as with CGM within a multiplet, we consider each XPL-only annulus as representing an annual hiatus.

Inferring an annual periodicity for atypical CGM such as XPL-only annuli is not without precedent. For instance, circumferential ‘polish lines’ (Sander, 2000) have been used in the absence of LAG and annuli to infer sauropod life histories and to model annual growth rates (*e.g*., Sander & Tückmantel, 2003; Klein & Sander, 2007). Although sometimes more than 60 microns in thickness, polish lines are not visible in polarized transmitted light, but readily visible in the same thin section by using reflected light. Sander (2000) inferred that they result from a less pronounced hiatus compared to that causing a LAG or annulus. He also observed that some of the polish lines in the outermost cortices of thin sections corresponded to visible LAG in thin section, supporting an interpretation of their annual periodicity. Polish lines have not, to our knowledge, been observed in any extant species but are nonetheless widely used as valid information on sauropod growth and development.

From our research, XPL-only annuli have not been previously described in detail in paleohistology reports, although a structure resembling an XPL-only annulus was briefly mentioned and figured in Curry Rogers et al. (2024, fig. 14E). “Supernumerary” lines were observed using XPL in a study on extant marine iguana osteohistology and do resemble XPL-only annuli, but the authors do not explicitly discuss how these marks should be interpreted in terms of growth cyclicity (Hugi & Sánchez-Villagra, 2012). Perhaps other reports of XPL-only annuli exist, but with terminology or phrasing that went undiscovered in our searches. Additionally, XPL-only annuli may have been included alongside typical annuli in previous publications without comment on their unusual characteristics, or they may have gone unnoticed in studies that only used PPL to assess growth marks. Interestingly, we found that XPL-only annuli were not present in a sample of the early theropod *Coelophysis* (from the Barta, Griffin & Norell, 2022 dataset) but we have identified them in the Jurassic theropod *Allosaurus* (from the Bybee, Lee & Lamm, 2006 dataset) and in Cretaceous ornithischians (H. Woodward, 2025, personal observations). As with polish lines and multiplets, identification and study of XPL-only annuli within extant vertebrates is necessary to understand exogenous and endogenous cues associated with their formation and to validate their periodicity. Therefore, to ensure our *Tyrannosaurus rex* species complex growth models can be compared with previous studies, we present variants where XPL-only annuli are excluded in NoX, and along with multiplets in NoXM.

Note that the presence of XPL-only annuli solves a serious problem for tyrannosaur skeletochronology; the sporadic occurrence of huge growth zones between adjacent CGM. For instance, the NoXM variants of tibiae BMRP 2004.4.4, MOR 009, MOR 1128 and BDM 050 growth series show very steep increases between some of their CGM (Fig. S2). Such large distances between consecutive CGM are problematic because they break the sigmoidal model of smooth continuous variation. These high annual growth increases are also responsible for considerable uncertainty (and width of the confidence band). 1–3 XPL-only annuli were typically found between these otherwise anomalously thick growth zones, resolving these steep increases in growth (Fig. S2, variant A).

The statistical evidence presented here supports interpretating XPL-only annuli as a new type of CGM for the *Tyrannosaurus rex* species complex. As demonstrated in the Results, inclusion of XPL-only annuli improves model fit and confidence band width, as truly extraneous data points typically reduce goodness of fit and increase uncertainty in the fit (including confidence band width). However, as stated earlier, because the evidence is tied to the tyrannosaur specimens in our study, further work must be done to validate XPL-only annuli in other contexts.

### *Tyrannosaurus rex* species complex growth and longevity

The smallest individual in our ontogenetic sample of the *Tyrannosaurus rex* species complex (MOR 1189) was at least ten years of age when it died based on tibia CGM count. The innermost (first) CGM is 71.8 cm in circumference, corresponding to a body mass of 26.9 kg (Campione & Evans, 2012). Since this body mass is achieved by farm-raised ostriches 60–90 days after hatching (Cilliers et al., 1995; World Ostrich Association, 2013; Ghavi Hossein-Zadeh, 2024) it is possible that the innermost CGM in MOR 1189 was formed at the end of its first year of life and that no CGM were lost to medullary expansion. However, it is also possible that medullary expansion erased one or more additional CGM. If this is the case, any additional missing CGM would add to the starting ages of all the specimens. At the other extreme of our sample, individual BDM 050 took between 27 and 42 years to achieve skeletal maturity, depending on which growth model variant is used.

Our technique is so far the only objective algorithmic method for determining specimen relative age and for combining multiple individuals to produce a taxon-level growth curve. Here, relative ages do vary from those previously reported for the same *Tyrannosaurus* specimens, but our results are better justified, being free of the extrapolations associated with using single specimens or an incomplete ontogenetic series. Using our technique we produced four growth curve variants from our *Tyrannosaurus rex* species complex dataset based on the treatment of XPL-only annuli and multiplets. Variant NoXM included the least number of data points and therefore had the steepest slope, highest peak growth rate (557 kg/year) and highest maximum percent annual growth rate (36.1%). It also had the largest 95% confidence band (*i.e*., most uncertainty) of the four variants. We discuss it here because it excludes XPL-only annuli and considers multiplets as CGM groupings occurring in the same year; thus, it allows us to directly compare our results to curves produced in previous *Tyrannosaurus rex* growth studies, which typically omit some multiplet CGM and do not report XPL-only annuli.

Variant A included CGM, XPL-only annuli, and considered each CGM in a multiplet as indicating one annual hiatus. Of the four, the curve of variant A modeled the lowest peak growth rate (360.7 kg/year) and lowest maximum percent annual growth rate (22.6%) for the *Tyrannosaurus rex* species complex, reflected by a protracted subadult stage and a growth asymptote between 35 and 40 years of age, or ~15 years later than predicted by the *Tyrannosaurus rex* curve of Erickson et al. (2004). Variant A had the most statistical support with the smallest confidence band, despite incorporating the most data points. We therefore argue that variant A most accurately models ontogenetic growth of the *Tyrannosaurus rex* species complex, despite its differences from previously published *Tyrannosaurus rex* growth models. As discussed above, primary tissue organization remained relatively constant throughout the ontogenetic series, so we hypothesize that the lower growth rates are a result of annual variation in hiatus duration (*i.e*., a shorter growing season resulting in less bone apposition).

#### Comparison to Erickson et al. (2004)

**Figure 15:**
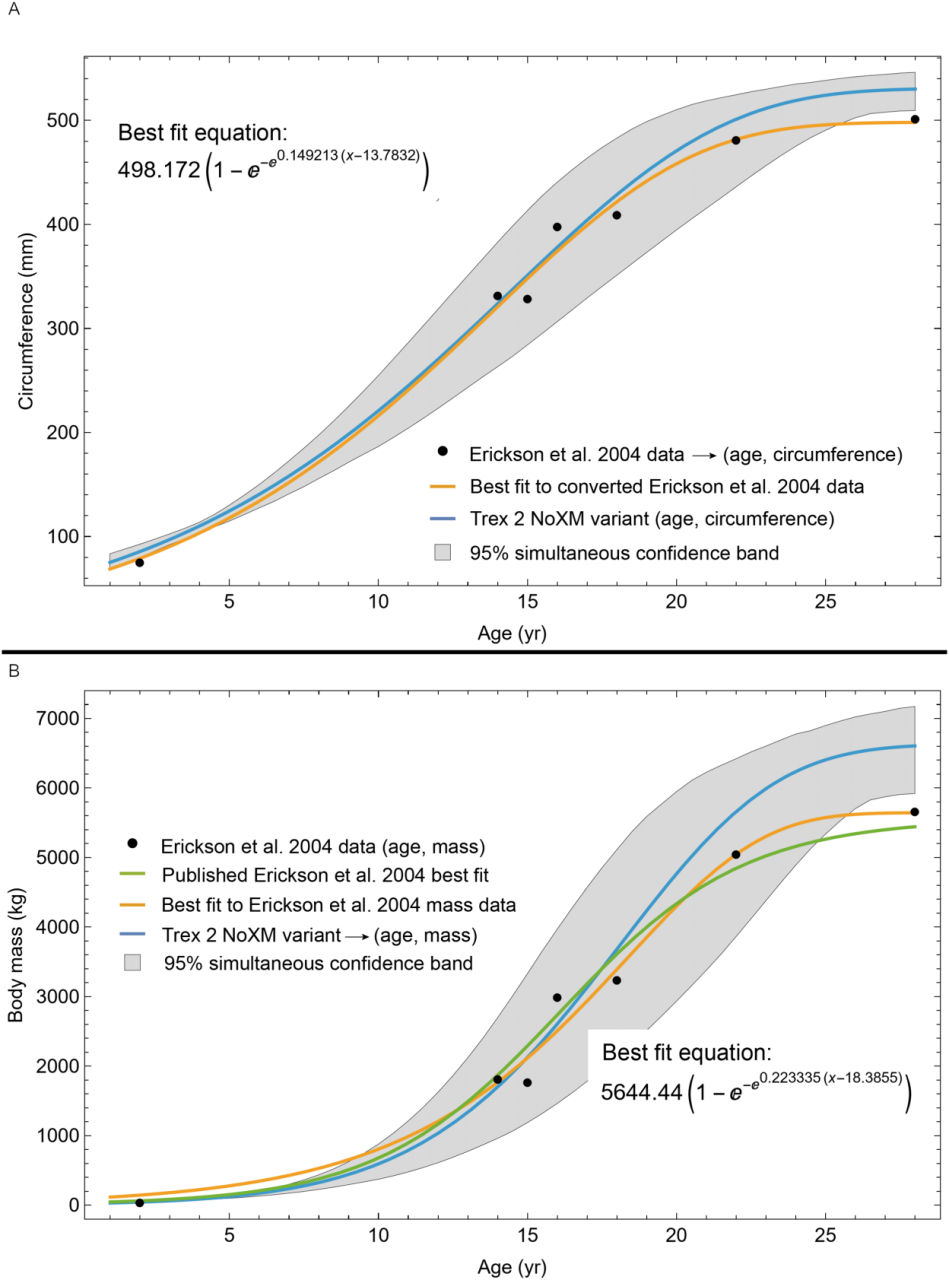
Comparison of present results to the *Tyrannosaurus* growth series of Erickson et al. (2004). (A) *T. rex* age data from Erickson et al. (2004) transformed from age, mass data to circumference (circ) data, plotted with Trex2 NoXM and its simultaneous confidence band. (B) The Erickson et al. (2004) data plotted with Trex2 NoXM transformed age, mass data. Further explanations in text.

There are notable methodological differences between our analysis and the initial *T. rex* growth study by Erickson et al. (2004). Their study used the whole-bone method, incorporating CGM data solely for age at death estimation. Starting ages for the seven specimens were subjectively estimated, and body mass was calculated using the Anderson formula (Anderson, Hall-Martin & Russell, 1985) based on femoral circumference; however, the actual femur circumference values were not published, only age and mass. Additionally, some inconsistencies are present—for example, the published growth curve does not align with the stated equation, and that equation does not match the best fit for the data when using the same four-parameter logistic function (Myhrvold, 2013; Erickson et al., 2016). Thus, to compare results, the Erickson et al. (2004) data must be transformed to circumference, or we must transform the growth curves from the current study to mass. Figure 15 shows both approaches. In Fig. 15A the Erickson et al. (2004) data is transformed to circumferences using the inverse of the Campione & Evans (2012) formula. This may introduce some error since it differs from the circumference to mass formula used in Erickson et al. (2004). The best fitting sigmoid to the transformed Erickson et al. (2004) data is the Erfc function shown in the plot. In Fig. 15B the Erickson et al. (2004) data is left in its original form while the regression curve and confidence band for Trex2 NoXM are transformed into mass using the Campione & Evans (2012) formula.

Despite the strong differences in methodology the correspondence is remarkable. The Erickson et al. (2004) data, and the best fitting sigmoid to that data, almost entirely lie within the 95% CB of the Trex2 NoXM case, for both transformations of the data (Fig. 15). The NoXM variant is the closest comparison to the results of Erickson et al. (2004) in that it uses the common practice of omitting some of the multiplet CGM and not using XPL CGM. Other variants would have a lower growth rate and result in a longer lifespan as shown in Fig. 10.

#### Comparison to Cullen et al. (2020)

The analysis of Erickson et al. (2004) included “Sue” (FMNH PR 2081), one of the world’s largest *Tyrannosaurus* (Mallon & Hone, 2024), as a whole-bone datapoint in their growth curve. In contrast, a longitudinal ontogenetic curve for Sue was published in Cullen et al. (2020) using 23 CGM data points. There are also distinct differences between the methodology of Cullen et al. (2020) when compared with our study. Their *Tyrannosaurus* CGM series is from a core sample at femur diaphysis, rather than a transverse section. As the entire CGM cannot be traced about the cortex, it is impossible to determine whether CGM observed within the core sample are double LAG, and neither multiplet lines nor XPL only lines are reported.

Another difference from our study is that Cullen et al. (2020) assume the periosteal circumference and all cortical CGM circumferences can be treated as perfect circles and can thus be scaled by distance from the periosteal surface. This causes unaddressed, and possibly quite substantial errors in their reported data as the nature of the scaling is such that the CGM closet to the periosteal surface will be known most accurately, and the uncertainty will increase with depth. This is not a theoretical objection to their methodology, as none of the 17 *Tyrannosaurus rex* species complex specimens examined in our study have perfectly circular cross sections.

Lastly, Cullen et al. (2020) estimate the youngest (starting) age for the CGM record of FMNH PR 2081 by extrapolating growth curves until they reach a hypothetical neonatal mass. Depending on the growth curve chosen, the FMNH PR 2081 CGM starting age is between 4 and 10 years. This method is cited as following Grady et al. (2014), which was previously criticized on several grounds (Myhrvold, 2016).

First, there is the problem of extrapolating a growth curve far from available data. The smallest CGM for FM PR 2081 has an estimated circumference of 338.7 mm which corresponds to a mass of 1,922 kg (using the Campione & Evans (2012) formula). While Cullen et al. (2020) do not provide an estimated neonatal mass, their methodology references Grady et al. (2014), who in turn cite Dolnik (2006). The latter presents an empirical formula for the egg size of extant birds as a function of adult mass (Dolnik, 2006). Using that formula, a hypothetical 7,000 kg adult *Tyrannosaurus* would have an egg weight of 234 g. This is about 10% to 20% the mass of the extant ostrich (*Struthio camelus*) egg which can range from 1.2 to 2.3 kg (Deeming, 2001), which is also very low compared to estimates from direct measurements of fossilized egg masses of other theropod species such as oviraptors (197–6,594 g) (Tanaka et al., 2018).

However, the primary concern is that extrapolating the growth curve from a starting age body mass of 1,900 kg down to 0.2 kg, or even 2.3 kg, is simply too far to have any statistical value (Myhrvold, 2013) as demonstrated by Fig. 3 from Cullen et al. (2020): although the four best-fit models are very competitive in fitting the actual data (by AICc), they differ in extrapolated starting age by a factor of 2.5. In other words, choosing a model by how well it fits large body size data (1,900 to 8,000 kg) reveals little about how well the model fits at a body mass of 0.2 g to 2 kg. If more models were added to their analysis, the starting age span would only grow.

There are also biological reasons to doubt the use of a single model across this body mass range, even if there was no extrapolation. Studies of extant altricial bird neonates show tremendously accelerated growth due to parental care (Ricklefs, 1968, 1973, 1979; Starck & Ricklefs, 1998). This pattern is so extreme that they generally achieve adult body size within the first year. The growth records of *Tyrannosaurus* show that it grew to adult body size over an extended period and thus did not fully follow the extant avian model. However, parental care is supported in some dinosaur species (Horner, 2000; Reisz et al., 2024; Bert et al., 2025), and it is unknown (but entirely possible) whether some degree of parental care could occur during in the earliest stages of life for *Tyrannosaurus*. If so, that period could be on an accelerated growth curve and would not be expected to follow the same growth model as later in life. This is the pattern observed in mammals that receive varying degrees of parental care, such as nursing (Riek, 2008; Adamczak et al., 2023). Thus, forcing an extension of the later stage growth model curve to age 0 involves an unwarranted assumption that early growth follows the same curve as later in life. For these reasons, we do not attempt to calculate neonate mass from which to estimate absolute age of the smallest CGM in our dataset.

**Figure 16:**
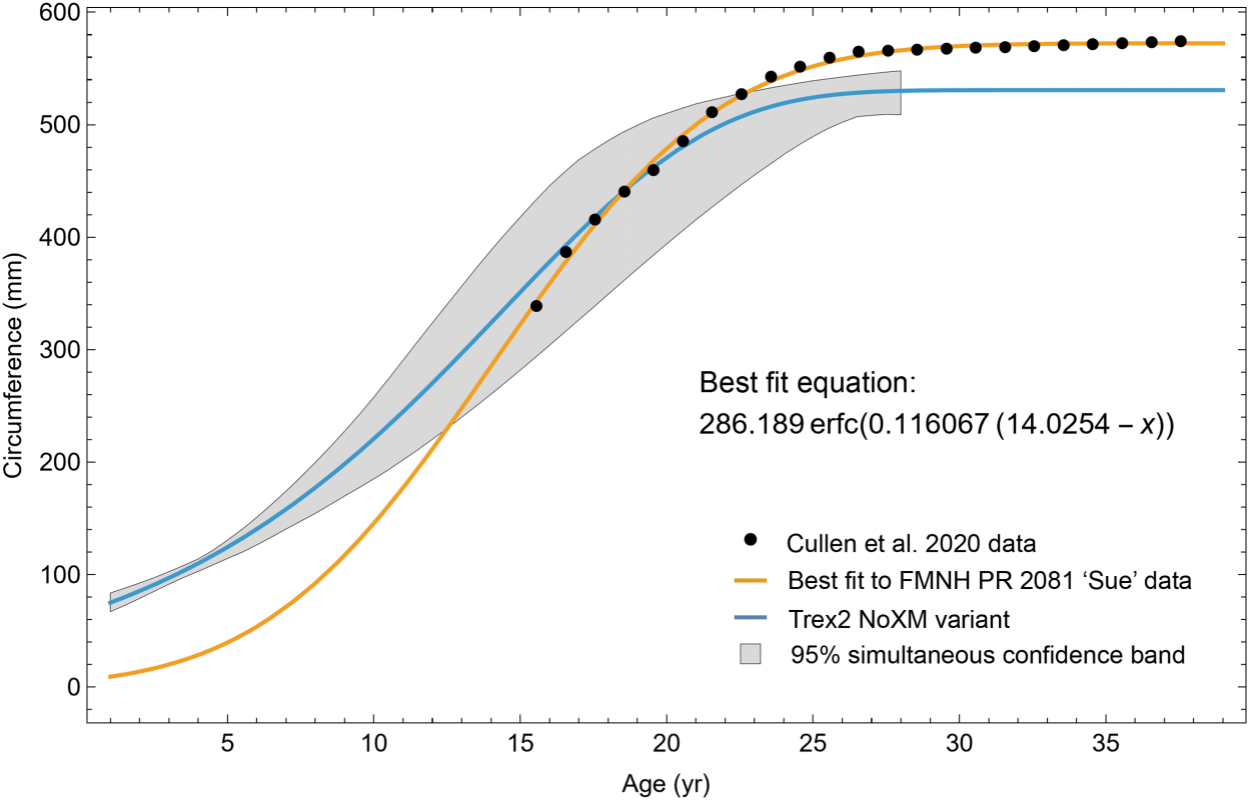
Comparison of present results to the *Tyrannosaurus* femur FMNH PR 2081 (“Sue”) growth series of Cullen et al. (2020). CGM data for FMNH PR 2081 using Cullen et al. (2020) is plotted (dots) and the earliest CGM is assigned a starting age of 14.6 years. A best fit sigmoidal curve using the erfc() function is plotted in gold. The NoXM variant using the Trex2 dataset is plotted in blue, and its simultaneous confidence band CB is the shaded area. Further explanations in text.

With those provisos we nevertheless compare the Cullen et al. (2020) data for FMNH PR 2081 “Sue” in Fig. 16. Using an approximation to the method used for the specimens in our study, we increase the CGM starting age for Sue to 14.6 years which minimizes the least squares difference between the Cullen et al. (2020) data and our Trex2 NoXM model.

Figure 16 also shows that FMNH PR 2081 is a very large specimen with a maximum femoral circumference (at the periosteal surface) of 574 mm. Using the Campione & Evans (2012) formula, this circumference corresponds to a body mass of 8,222 kg. In Erickson et al. (2004), FMNH PR 2081 was estimated to have a mass of 5,654 kg, or slightly over two-thirds of the more recent mass estimate. The reason that our results are highly compatible with Erickson et al. (2004) and less so with Cullen et al. (2020), both of which use FMNH PR 2081 as their largest specimen, is due to the impact of the different mass estimating formulas used in 2004 and 2016. In contrast, the largest specimen in Trex2 (MOR 1125) has a femur circumference of 546 mm, corresponding to 7,175 kg using Campione & Evans (2012), or about 1,000 kg lighter than FMNH PR 2081.

In addition to the body size difference between Sue and the specimens in our study, there is also a difference in how long individuals lived at or near maximum size. From our sample, *Tyrannosaurus rex* species complex specimens CCM V33.1.15 and BDM 050 died soon after reaching their full adult size based on the few CGM comprising their relatively thin incipient EFS. Not so with FMNH PR 2081. Of the 23 CGM reported by Cullen et al. (2020), about half of them (11 CGM) are within the EFS, reflecting a period negligible growth in femoral circumference. Moreover, 10 of these CGM surpass the largest CGM circumference size in our data. Assuming CGM are formed annually within the EFS, FMNH PR 2081 spent at least 11 years at its (very large) adult size.

Considering the differences in methodology between Cullen et al. (2020) and the present study, we regard the growth curves shown in Fig. 16 as being in relatively good agreement. FMNH PR 2081 is clearly much larger and lived longer in its final (EFS) stage than any of our specimens. But any growth study must be revised upward when larger specimens become available. For instance, recent publications report a *Tyrannosaurus* (RSM P2523.8) with a 590 mm femur circumference and an estimated mass of 8,870 kg (Persons, Currie & Erickson, 2020; Mallon & Hone, 2024) or 648 kg more than FMNH PR 2081, and 1,695 kg larger than MOR 1125. Moreover, a recent theoretical study used a statistical model to estimate the upper limit of *Tyrannosaurus* body mass to be 1.7 times larger than RSM P2523.8, with a modeled circumference of 717 mm and estimated mass of 15,000 kg (Mallon & Hone, 2024).

#### Comparison to Longrich & Saitta (2024)

In their arguments to support the validity of *Nanotyrannus*, Longrich & Saitta (2024) present a *Tyrannosaurus rex* growth curve using FMNH PR 2081 data from Cullen et al. (2020). They compare that curve with curves of BMRP 2006.4.4 and BMRP 2002.4.1 made from the data in Cullen et al. (2020), which was modified (multiplets removed) from Woodward et al. (2020). Longrich & Saitta (2024) present no new data for these specimens.

#### Comparison to Mallon & Hone (2024)

The *Tyrannosaurus rex* growth curve presented by Mallon & Hone (2024) is unusual in that it returns to the whole-bone method of Erickson et al. (2004), with each specimen represented by a single age and body mass. Age at death estimates as well as limb circumference measurements and estimates were apparently obtained from the literature. Rather than constrain the neonate size as in Cullen et al. (2020), Mallon & Hone (2024) add a neonate data point with body mass of 2 kg to the dataset. The result is a growth curve that, as the authors admit, predicts “negligible growth in the first decade of life (but see Section 3), followed by explosive growth during the teenage years”. Lastly, their published growth model, (their Eq. (2)), does not match their Fig. 1 plot and is likely compromised by a typographical error (Mallon & Hone, 2024).

#### Maximum growth rate estimation

Starting with a series of articles by Erickson & Tumanova (2000), Erickson, Rogers & Yerby (2001), Erickson et al. (2004, 2009), Grady et al. (2014), Werner & Griebeler (2014), maximum growth rate has been one of the key statistics quoted in previous dinosaur growth rate studies. Before presenting our results for the *Tyrannosaurus rex* species complex, it is helpful to explain the history of the topic. The original motivation for determining the maximum growth rate statistic was the assertion that by following two classic articles by Case (1978a, 1978b) one could classify dinosaur metabolism as similar to either that of extant reptiles, birds, or fish. Initially this classification was simply applied and reported in publications, but later articles by Grady et al. (2014) and Werner & Griebeler (2014) sought to put the hypothesized Case correlation on firmer ground with broad extant vertebrate datasets. Unfortunately, the two studies reached mutually incompatible conclusions, and both suffered statistical and phylogenetic flaws (Myhrvold, 2016). Myhrvold (2016) resolved this by showing there is no basis for a correlation between metabolism and maximum growth rate, (as also disclaimed by Case in the original articles).

A second motivation for examining maximum growth rate is the idea that one could distinguish whether dinosaurs attained their large body size through the mechanism of “acceleration” (more properly, higher growth rates) or “prolongation” (*i.e*., growing at the same rate for a longer period of time). However, D’Emic et al. (2023) showed this is a false dichotomy if interpreted as two discrete and mutually exclusive strategies. Instead, they find multiple strategies, or combinations thereof, within dinosaur clades. In other words, maximum growth rate alone is uninformative; a “growth strategy” is encapsulated by the growth curve, which is a precise mathematical description of both growth rate and its duration.

**Figure 17:**
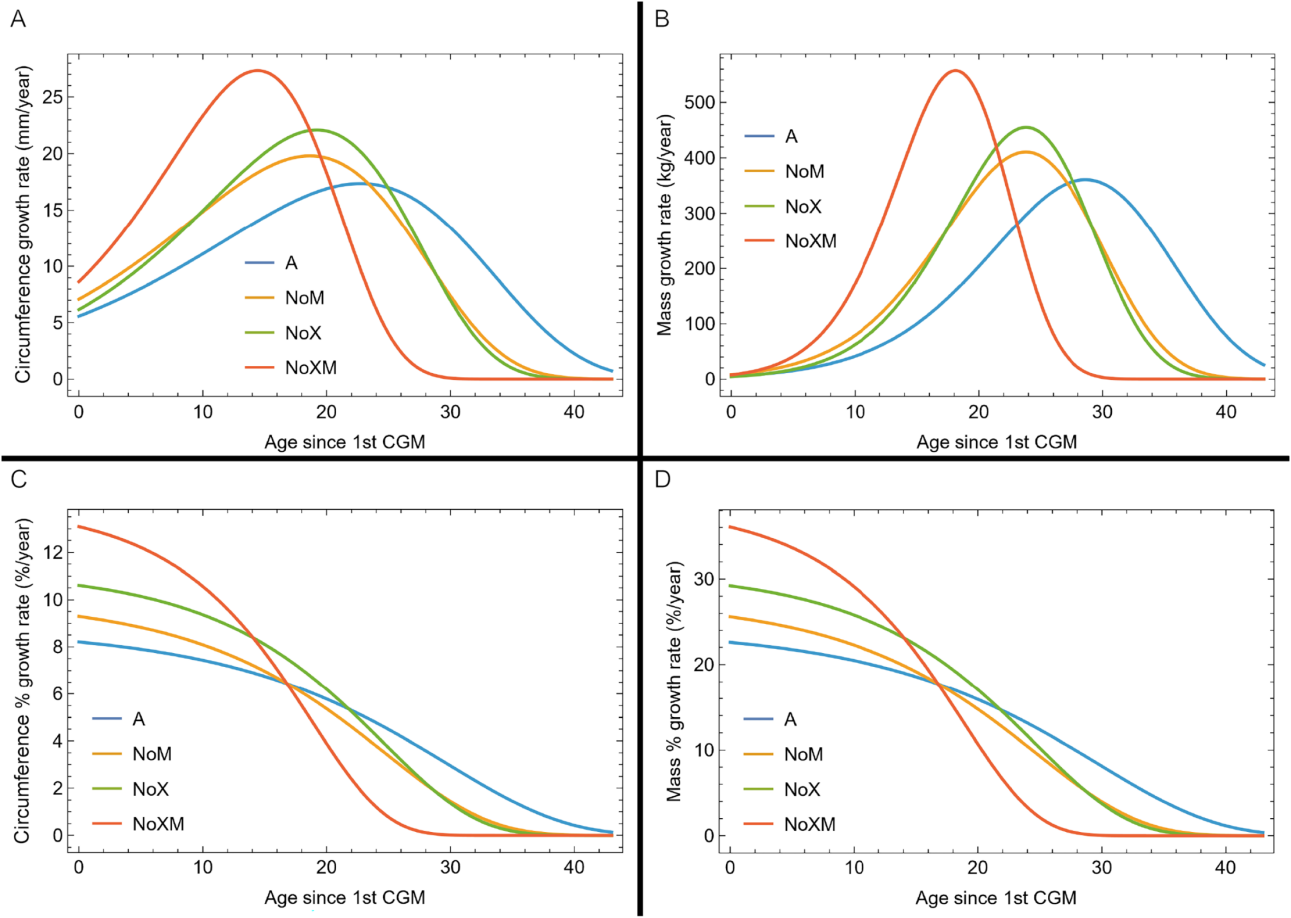
Different ways to view growth rate for the Trex2 dataset variants. (A) Growth rate can be defined as a linear measure of bone growth, *i.e.*, CGM circumference increase per year, (B) by absolute mass increase per year, (C) by the relative (percentage) circumference, or (D) the relative (percentage) mass increase per year. Each panel shows the curves derived from the best fitting models plotted in Fig. 10. The inflection point is the age at which the growth rate is maximum and this value is not the same across CGM variants. It occurs at the 1 st CGM for percentage growth for all variants, but its location differs for circumference *vs*. mass.

With the history of maximum growth rate estimation in mind, Fig. 17 shows four different growth rate plots for the Trex2 dataset, across the four variants. The values for maximum growth rate and corresponding age are given in Table 6. Figure 17 shows the most basic measure of growth rate, which tracks the growth of the limb bone, as a linear increase in CGM circumference per year. Here the Campione & Evans (2012) bipedal formula is arguably better than the Anderson, Hall-Martin & Russell (1985) formula, but it may not be as accurate as the increasingly popular, but labor intensive technique of using detailed 3D modeling to reconstruct tissue masses, for example (Sereno et al., 2022).

**Table 6:** Determination of maximum growth rate by various definitions, for each variant of dataset Trex2.

Definition	Units	Variant A	Variant NoM	Variant NoX	Variant NoXM
CGM circumference maximum growth rate	mm/yr	17.4	19.8	22.1	27.3
Inflection point	yr	22.7	18.6	19.2	14.4
Body mass maximum growth rate	kg/yr	360.7	410.7	455.0	557.2
Inflection point	yr	28.6	23.8	23.8	18.1
Circumference maximum % growth rate	%/yr	8.2%	9.3%	10.6%	13.1%
Inflection point	yr	0	0	0	0
Body mass maximum % growth rate	%/yr	22.6%	25.6%	29.2%	36.1%
Inflection point	yr	0	0	0	0

It is well known that different sigmoid functions reach their maximum growth rate (and thus their inflection point) at different ages (Ratkowsky, 1983; Bates & Watts, 1988; Seber & Wild, 2003). Although counterintuitive, converting a circumference growth curve to mass is a nonlinear transformation—and thus it will change the location of the maximum growth rate (*i.e*., the inflection point). The age at which maximum growth rate occurs also differs depending on the sigmoid model.

There is also a difference between growth rate on an absolute basis (mm/year or kg/year) *vs*. relative growth rate (percentage increase per year). For instance, a relatively modest 36.1% maximum mass increase occurs during the year of CGM 1 for variant NoXM. By comparison, the absolute maximum growth rate (557.2 kg/year) at CGM 19.1 is only a 14.9% body mass increase from the previous year. Whichever metric of growth is chosen will influence the location of the inflection point and what it might mean physiologically. If the percentage mass increase (% mass increase per year) is regarded as the proper metric, then the inflection point occurs at age 0 (relative to CGM 1) for all of the variants. Having the inflection point at age 0 is a well-known feature of the monomolecular sigmoid function and has been used previously in dinosaur growth modeling (*e.g*., Myhrvold, 2013; Woodward et al., 2015). Using absolute mass growth (kg body mass per year) as the metric moves the inflection point to between 18 and 28 years depending on the variant. In our view the rationale behind treating the inflection point as having biological significance is undermined because its value changes completely depending upon the units by which growth is measured.

### Problematic specimens

An important and rather surprising result is that the growth trajectories of BMRP 2006.4.4 and BMRP 2002.4.1 are statistically incompatible with those of other *Tyrannosaurus* in the Trex2 dataset. This observation is supported in a quantitative manner by the comparatively lower mean growth rate values of the BMRP individuals (Table 7). The synthetic data series experiment (Figs. 8, 9, S21, S22) also demonstrate that the slopes of the BMRP growth series are much lower than the synthetic slope which minimizes the CB area/length. At the same time, the BMRP 2006.4.4 and BMRP 2002.4.1 growth series are visually similar, being roughly parallel to one another for all variants (Figs. S23, S24). However, it should be noted that per-variant plots (Figs. S5, S6, S16, S17) show that the impact of the omission of BMRP 2006.4.4 and BMRP 2002.4.1 does not affect each variant equally. The removal of the BMRP 2006.4.4 data series in the 1^st^ round only affects the A, NoM, and NoXM variants. After removing BMRP 2006.4.4, in the second round removing BMRP 2002.4.1 only affects the NoM, Nox and NoXM variants, and has little impact on the CB area/length of the A variant.

**Table 7:** Mean growth rates for BMRP 2002.4.1 and BMRP 2006.4.4, and for synthetic series trials. The BMRP 2006.4.4 and BMRP 2002.4.1 mean CGM circumference growth rate (slope of best fit line) is shown for each variant in units of mm/year. Different rates are expected for each variant because they differ in number of points over approximately the same range of circumference. The bottom row shows the growth rate which minimizes confidence band (CB) area/length in the synthetic series (see Fig. 8).

Specimen	Variant A	Variant NoM	Variant NoX	Variant NoXM
Tibia BMRP 2002.4.1 (mm/year)	8.85	10.15	8.85	10.15
CGM count	13	11	13	11
Tibia BMRP 2006.4.4 (mm/year)	5.96	8.62	7.65	13.67
CGM count	22	13	18	9
Femur BMRP 2006.4.4 (mm/year)	7.55	8.69	8.08	9.33
CGM count	15	12	14	11
Synthetic slope at minimum CB area/length (mm/year)	14	24	20	30

Thus, a growth study that focused only on the A variant (the variant with the most statistical support in the study) would omit BMRP 2006.4.4, but not BMRP 2002.4.1. A study that only focused on the NoXM variant (consistent with the way previous studies treat XPL annuli and multiplets) would omit BMRP 2006.4.4, and BMRP 2002.4.1 would be eliminated in a second round. A possible reason for the different impact of including these taxa for each variant is the length of the data series. The shorter the growth series, the more likely that it can be accommodated. Table 7 shows that the lengths of the two BMRP series are roughly the same in the NoXM variant (from 9 to 11 CGM), but quite disparate in the A variant (13 to 22) CGM. This is further explored in Fig. S24 which annotates the A variant to show which datapoints are added by the treatment of multiplet or XPL-only annuli.

Despite the large differences in the number of CGM between the variants, the range of growth rates across the three specimens (tibia of BMRP 2002.4.1, femur and tibia of BMRP 2006.4.4) is smaller for the A than NoXM variants. The absolute range in growth rates for A is 2.88 mm/year between lowest and highest rates, *vs*. 4.34 for NoXM. On a relative basis the difference in rates is 38% for A and 39% for NoXM.

One possible explanation for the negative impact of BMRP 2006.4.4 and 2002.4.1 on the composite curve is that these specimens are not *Tyrannosaurus rex*. For over 70 years, paleontologists have considered whether such small-bodied tyrannosaurs from the Maastrichtian of western North America represent juvenile *Tyrannosaurus rex* or are adults of a different taxon (*e.g*., Gilmore, 1946; Rozhdestvensky, 1965; Bakker, Williams & Currie, 1988; Carr & Williamson, 2004; Larson, 2008, 2013; Witmer & Ridgely, 2010; Tsuihiji et al., 2011; Brusatte et al., 2016; Schmerge & Rothschild, 2016; Carr, 2020; Woodward et al., 2020; Longrich & Saitta, 2024) most recently referred to as *Nanotyrannus* (Bakker, Williams & Currie, 1988). Indeed, based primarily on morphological arguments, several studies assign BMRP 2002.4.1 and BMRP 2006.4.4 to *Nanotyrannus* (Larson, 2008, 2013; Tsuihiji et al., 2011; Schmerge & Rothschild, 2016; Longrich & Saitta, 2024), while others disagree (Carr, 2020; Voris et al., 2025). However, despite being larger than the inferred adult type *Nanotyrannus* CMNH 7541 (Bakker, Williams & Currie, 1988), no EFS was observed in the BMRP specimens, indicating that they had not reached skeletal maturity prior to death (Woodward et al., 2020).

Our careful re-examination of BMRP 2006.4.4 and BMRP 2002.4.1 upholds the finding of Woodward et al. (2020); while BMRP 2006.4.4 and BMRP 2002.4.1 were growing more slowly compared to the other specimens, there is no evidence they had stopped growing. Unfortunately, the inferred adult type specimen of *Nanotyrannus* (CMNH 7541; Bakker, Williams & Currie, 1988) does not include long bones, making it impossible to directly compare its growth rate to specimens BMRP 2006.4.4 and BMRP 2002.4.1 using the methods presented here.

An even smaller tyrannosaur in our sample, DDM 35, was also recently referred to as *Nanotyrannus* based on skeletal morphology (Longrich & Saitta, 2024). Our histological assessment shows that like BMRP 2006.4.4 and BMRP 2002.4.1, it was also skeletally immature prior to death. But unlike the BMRP specimens there is no evidence that including DDM 35 with the *Tyrannosaurus* species complex dataset harms our growth curve or the confidence band, although its relatively short growth sequence may ameliorate some of the impact.

Another possibility for the slow growth and advanced age of BMRP 2006.4.4 and BMRP 2002.4.1 may be due to atypical growth histories. For instance, the femur and tibia of BMRP 2006.4.4 have much different growth records (15 CGM in the femur, 23 CGM in the tibia; (Table S1, Fig. S23) with the femur having more multiplets than any other specimen—a total of 6 out of 15 CGM. Even if one considers each of these CGM groupings to represent numerous hiatuses in a single year, their frequency over such a short period still suggests a pathological or otherwise abnormal growth pattern which might be due to injury, illness or environmental issues.

As an example, Dalrymple (1996) demonstrated that alligators living in the Everglades exhibited “slow growth and mature at smaller size and at greater age likely due to resource limitation.” But this is not simply a crocodilian feature; there are classic cases where typically large bodied mammals evolve smaller body sizes generally due to environmental resource constraints (Lomolino, 2005; Van der Geer, Lyras & Vos, 2021), and this reduction con occur quite rapidly over several generations (Rozzi & Lomolino, 2017). For instance, *Mammoth primigenius* weighed up to 6 tons, while its descendants on Wrangel Island in the arctic were 2.6 tons and those in Sicily were an estimated 187 kg (Rozzi & Lomolino, 2017). Within extant African elephants, the forest elephant *Loxodonta cyclotis* (2.5 tons) (Morgan & Lee, 2003; Gobush et al., 2021) and the savanna elephant *Loxodonta africana* (6.9 tons) (Nowak, 1999) are recognized as separate species, in this case having evolved their disparate sizes from a common ancestor in adjacent ecosystems within a large continent rather than through insular, island isolation. If only found in the fossil record, it would be difficult to determine whether *L. cyclotis* and *L. africana* were indeed separate species. Similarly, although considered a single species, the extinct American lion (*Panthera atrox*) exhibits much larger body sizes in specimens from Alaska (Harington, 1969) than in southern California (Wheeler & Jefferson, 2009).

We offer these examples not because they are good growth models for *Tyrannosaurus*. Rather, they demonstrate that evolutionary pressures can shift growth rates and body size in response to geographic or environmental factors and do so rapidly. In other words, the capacity for extreme growth plasticity in extinct and extant archosaurs (*e.g*., Hutton, 1986; Jacobsen & Kushlan, 1989; Sander & Klein, 2005; Woodward et al., 2015, 2020; Barta, Griffin & Norell, 2022) including birds (Canoville, Chinsamy & Angst, 2022; Ong et al., 2022), and probable ontogenetic niche partitioning in tyrannosaurs (Holtz, 2008; Brusatte et al., 2010; Kane et al., 2016; Woodward et al., 2020) could result in decreased growth rate and prolonged retention of small body size if the individuals were living in a resource poor environment. Indeed, the BMRP specimens were collected within 5 km of each other in southeastern Montana and may have experienced similar geographically localized paleoenvironmental stressors. To test this hypothesis would require confirmation that the BMRP specimens were excavated from the same stratigraphic horizon and evidence of that stratigraphic interval exhibiting a paleoenvironment differing from that of the Hell Creek Formation more generally.

As a source of systematically slower growth we must also consider pathology, which has been noted in BMRP 2002.4.1 (Vittore & Henderson, 2013) and proposed in BMRP 2006.4.4 (Tremaine, Ballard & Horner, 2014). Comparison with quality growth curves from the long bones of additional tyrannosaur specimens are required to test this hypothesis.

While various proposals in the literature recognize *Nanotyrannus* as a valid genus, still others split *Tyrannosaurus rex* into multiple taxa (*e.g*., Paul, Persons & Raalte, 2022; Paul, 2025) on the basis of morphology. Our *Tyrannosaurus rex* species complex dataset includes specimens from the lower, middle and upper thirds of the Hell Creek Formation. Excluding BMRP 2006.4.4 and BMRP 2002.4.1 and using our methods, we do not find evidence of further divergences from the growth rate data that would suggest multiple *Tyrannosaurus* species based on differences in growth patterns alone.

Lastly, Jevnikar & Zanno (2021) reported that the growth trajectory of BMRP 2006.4.4 could not be linked to those of uncontroversial *T. rex* specimens modeled in their study. Otherwise, the authors found a bimodal distribution in their sample and hypothesized that it may be due at least in part to cryptic sexual size dimorphism (SSD) (Jevnikar & Zanno, 2021). Here, we address the possibility that SSD may explain the growth rate differences observed between the BMRP specimens and the rest of our dataset. While SSD is observed in many extant species where male and female individuals have different adult body size, SSD seems unlikely for the BMRP specimens because it would imply they are one sex and the other 10 specimens in our study are the opposite sex. Where it exists, the larger sex/smaller sex SSD ratio for body mass across birds, mammals, and reptiles is typically in the range 10% to 20% (Fairbairn, 1997; Fairbairn, Blanckenhorn & Székely, 2007; Tombak, Hex & Rubenstein, 2024). After removing the BMRP individuals to make the Trex2 dataset, there is +12.6% to – 8.5% variation (95% confidence interval) in the residuals for limb circumference across the remaining 10 specimens (Fig. S18). This roughly translates to 1.8× variation in body mass. Thus, larger sex/smaller sex SSD ratios from 0 to 1.5 or somewhat higher would easily be accommodated in our current results. In other words, our current dataset cannot resolve cryptic SSD if the ratio falls within the typical 10-20% range seen in most vertebrates displaying SSD.

**Figure 18:**
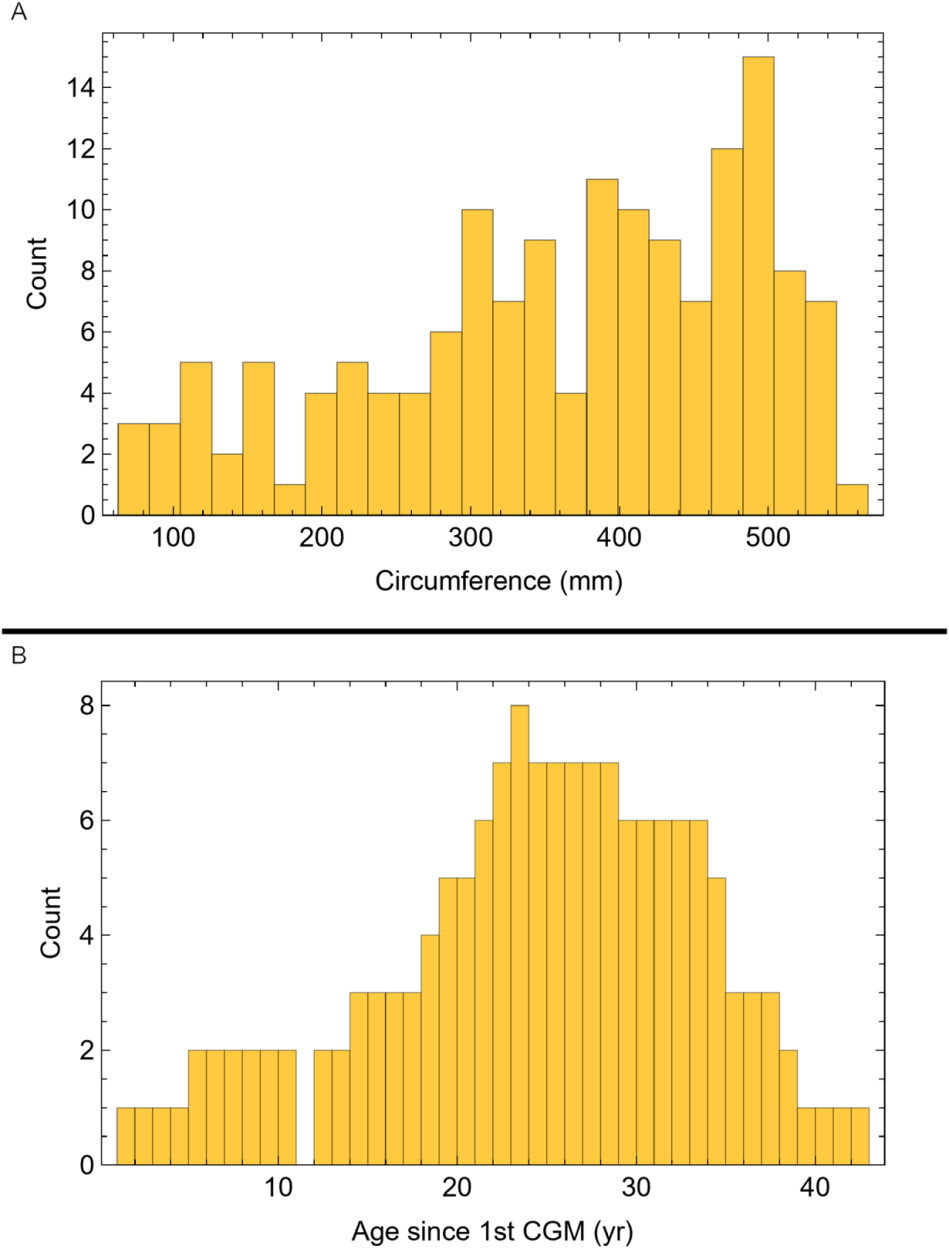
Histograms of the CGM circumference and estimated age in the Trex 2 dataset. (A) A count of specimens which present a CGM within binned circumference sizes. There are no very small CGM (<71.83 mm circumference), and only one example at the very largest end of the circumference range which is 546.3 mm. (B) The same data as in A, but here based on age since the first CGM.

SSD will also go undetected in specimens which have not reached their asymptotic limit (*i.e*., adult size). Unfortunately, six of the 10 specimens in Trex2 dataset end well short of their asymptote (Fig. 18). With so few mature specimens we lack the resolution to identify possible SSD at the specimen level, or even to be certain that it is present.

Ultimately, we cannot favor any of these hypotheses to explain the growth rate differences observed in the BMRP specimens compared to the rest of the dataset. Indeed, we cannot even be certain that the growth of the two specimens is comparable due to the much shorter growth series of BMRP 2002.4.1. To further explore the possibility that the BMRP specimens are a different taxon within the *Tyrannosaurus rex* species complex, that they exhibit adaptation to local environmental conditions, or display sexual size dimorphism (all of which are not mutually exclusive hypotheses) requires evidence beyond growth data and thus lies outside the scope of the present study.

### Predicting onset of sexual maturity

One possible consequence of finding that individuals in the *Tyrannosaurus rex* species complex are older than previously estimated is a follow-on effect for other life history parameters such as the age of sexual maturity. Lee & Werning (2008) proposed that age at reproductive maturity (RM in their terminology) in non-avialan dinosaur taxa occurred at the inflection point of the absolute body mass curve, which for the sigmoid curves they examined occurs at one third to one half adult body mass (p. 582):

“The growth inflection marks the transition from growth acceleration to deceleration and is of physiological interest because it generally coincides with the onset of RM in extant animals (14,15).”

Intuitively, the inflection marks the point in ontogeny at which the organism begins investing a decreasing amount of its energy into growth, thereby freeing up energy for reproduction. Like many comparative studies, Lee & Werning (2008) presented a way to apply a well-established principle from extant animal physiology to dinosaurs, which has since been widely applied (*e.g*., Sander, 2000; Hedrick, Tumarkin-Deratzian & Dodson, 2014; Waskow & Sander, 2014; Woodward et al., 2015; Padian & de Ricqlès, 2020; Turner Tomaszewicz et al., 2022; Chapelle, Griffin & Pol, 2025, and references therein). However, careful review of their reference 14 (Brody, 1964) and 15 (Reiss, 1989) show little if any support for of a well-established link between sexual maturity and the growth inflection point.

Brody (1964) concerns growth curves for domestic livestock such as pigs, cattle and sheep, with occasional mention of other mammalian species such as laboratory rats, humans or chimpanzees. It mentions the inflection point as a marker of “puberty”, but more as a statement than a conclusion backed by evidence across many species. The proposed relationship between puberty and inflection point seems to originate from his earlier observations. Brody (1927) previously proposed that one can normalize growth curves for different species and thus give an “age equivalence”. For example, he proposed that when normalized, 1 month of growth for a laboratory rat is “equivalent” to 11.91 months growth in a Jersey cow. A consequence of this “equivalence” is that puberty should occur at the same “equivalent age”, which Brody associated with the inflection point, both for the mammals he studied and even for angiosperms (with respect to age of flowering). Additionally, Brody did not use sigmoidal curves. He instead modeled growth using two curves; an exponential early in life, and a complimentary exponential (sometimes called the Brody model) later in life. His “inflection point” is the intersection of these two curves rather than an inflection point defined by the peak growth rate in a single model. As shown above in Table 6, and Fig. 17 the entire concept of inflection point changes utterly if growth is measured on a bone length *vs*. mass basis, and whether it is absolute numbers or percentages. If growth is measured by absolute mass increase, the inflection point is where the mass increases at a slower rate. But as a percentage of growth, or if a linear metric of body size is measured rather than mass, the result changes. Thus, the idea that there is a unique (and therefore biologically meaningful) inflection point is refuted.

Armed with more data and vastly better computational resources and statistical tools, we now know that more than one growth curve model should be used to study animal growth, because some fit data for a species better than others (Leberg et al., 1989; Katsanevakis, 2006; Teleken, Galvão & Robazza, 2017; Adamczak et al., 2023). Thus, there is no universal growth curve, and there is no age equivalence as a fixed number; growth fit by different models would make the age equivalence a complicated function of time rather than a fixed scalar parameter. Recent works have shown that Brody’s link between inflection point and sexual maturity fails even within the scope of domestic livestock that were his primary subject (Pittroff et al., 2008; Myhrvold, 2013; Teleken, Galvão & Robazza, 2017).

The other inflection point reference from Lee & Werning (2008) is Reiss (1989), which describes a theoretical argument that “age at maturity” should occur when the mass of the animal reaches ${W^{0.3}}$, where $W$ is the asymptotic (full grown) body mass. This is not supported by empirical data for a wide set of extant species and instead is justified on purely theoretical grounds. Note that this finding is functionally different than mass at the inflection point, which depends on the model but generally is a fraction of asymptotic mass $W$ (*i.e*., $W/2$ for a logistic model (Goshu & Koya, 2013; Myhrvold, 2013)) rather than $W$ to a fractional power. The ${W^{0.3}}\;$ threshold is also extremely low, meaning for instance that for a hypothetical 7,000 kg *Tyrannosaurus*, the body mass at sexual maturity would be only 14.2 kg.

Dinosaur age at maturity was directly addressed by Erickson et al. (2007), who studied the histology of dinosaurs preserved brooding eggs on nests, inferring that brooding behavior is an unambiguous marker of reproduction. Unfortunately, the study could not provide a lower bound on the age at sexual maturity: of the 7 specimens studied, 5 had an EFS and thus were fully grown, and the remaining two were past their inflection point (defined as the absolute mass growth per year).

We find no other articles prior to Lee & Werning (2008) proposing that sexual maturity is associated with the growth inflection point based on empirical studies of extant animals, but the relationship between growth rate and sexual maturity in mammals and birds was later studied in detail by Ricklefs (2010). The mammal taxa studied failed to follow any consistent pattern between growth rate, inflection and sexual maturity. On the other hand, birds generally have extremely rapid growth to adult size, and this is reflected in Ricklef’s data which show nearly all the bird species studied reproduced at 100% of their asymptotic mass. This is not the inflection point—it is the asymptote—so birds as a group also do not follow the inflection point reproductive maturity model. Using *Alligator* as another archosaur example, sexual maturity is known to coincide with a body length threshold of 1.8 m (Lance, 1989; Brisbin & King, 1990) but the age at which this body length is reached can vary across both individuals and populations. For instance, the average length of annual hiatus is lower for alligators in Louisiana compared to North Carolina, so male alligators in Louisiana are sexually mature in 10 years while those in North Carolina attain sexual maturity by 18 years (Lance, 1989). Lastly, Griebeler, Klein & Sander (2013) found very little direct evidence for inflection point as the marker of sexual maturity in earlier studies of extant animals. They also make the important observation that specific sigmoidal growth models (*i.e.*, logistic, Gompertz *etc*.) have their inflection point at different characteristic ages, (and different fractions of asymptotic mass), so studies which use one or a limited number of models have, in effect, pre-chosen the inflection point rather than deriving it from the data. Such data cannot be used to study the inflection point.

While the inflection point can only be determined by fitting a growth model, it is related to “rapprochement”, a qualitative assessment of the shift from wide cortical zonal thicknesses to consecutively thinner zonal spacing (Francillon-Vieillot, Arntzen & Geraudie, 1990). Rapprochement was first proposed to coincide with sexual maturity in *Necturus* salamanders (Senning, 1940), but (Francillon-Vieillot, Arntzen & Geraudie, 1990) later noted that the relationship varied across the different salamander taxa studied: in some taxa the two phenomena coincided, but in others sexual maturity and rapprochement occurred asynchronously. Despite this variability, histological investigations on extant lissamphibians and non-avialan sauropsids depend heavily on Senning (1940) and Francillon-Vieillot, Arntzen & Geraudie (1990) to conclude a relationship between rapprochement and sexual maturity within their samples.

The relationship between either inflection point or rapprochement as markers of sexual maturity has a basis that is uncertain at best. Well-studied extant tetrapods are highly variable and do not exhibit a clear pattern. Applying an unproven relationship that fails for many well-known species (including avialan dinosaurs) to infer reproductive maturity timing in non-avialan dinosaurs seems even more speculative, including application to the *Tyrannosaurus rex* species complex growth series studied here.

### Dataset limitations

Although this study presents the largest growth series for the *Tyrannosaurus rex* species complex both in terms of specimen count and CGM count, there are still some limitations inherent in the data, primarily due to sampling bias in the fossil record. Figure 18 plots histograms of CGM circumference and estimated age. The largest bin count is not the largest circumference; there are 14 specimens that have CGM within the size bin that includes 500 mm, but as one moves to larger bins the count sharply decreases. There is only one specimen in the largest bin (546.3 mm). As discussed above, *Tyrannosaurus* larger than those included in our dataset are already known but incorporating them would only shift bins slightly to the right. Because the largest specimens are likely to be rare for biological reasons, their absence represents a longstanding bias in the fossil record (Benson, 2018; Persons, Currie & Erickson, 2020; Mallon & Hone, 2024). At the small end of the size spectrum there is no specimen with a CGM circumference less than 71.83 mm, likely due to taphonomic biases. Additionally, while a specimen that achieves a maximum CGM circumference in the 500 mm bin will contribute between 10–20 CGM, a very small (and thus young) specimen can only contribute a few. This is reflected in the age histogram (Fig. 18). There are at least six different specimens with CGMs representing between 22 and 32 years, but many fewer as one moves to either younger or older ages. The same biases are at work here—the very young (small) specimens are likely pruned by taphonomic bias and the very old (large) specimens by the realities of survival to old age and thus relative rareness. Both size and age biases have long plagued dinosaur growth studies (Myhrvold, 2013).

Thus, our growth series achieves its best accuracy in modeling middle sized, middle aged *Tyrannosaurus*. One can hope that future discoveries will improve on this by allowing inclusion of more specimens at the extremes of size and age. However, it is also possible that while we can improve the absolute specimen counts in the small (young) and large (old) ranges, we likely will find even more data in the center of the histograms so the relative paucity at the extremes will still be with us.

## Conclusions

*Tyrannosaurus rex* and its species complex represents a critical taxon for understanding Late Cretaceous ecosystems, theropod diversity, and the evolutionary dynamics preceding the end-Cretaceous extinction. Its prominence in both the scientific literature and popular culture underscores its enduring relevance as a subject of multidisciplinary research. With femora and tibiae from seventeen individuals, our osteohistology-based life history investigation of the *Tyrannosaurus rex* species complex is the largest and most ontogenetically complete to date.

We are the first to apply a statistical method to dinosaur skeletochronology that estimates the relative age of the first (innermost) preserved CGM in each individual in a sample to concurrently fit sigmoidal growth curves to data from multiple specimens. This produces a composite growth series and a generalized taxonomic growth curve which should better represent a population. Our method is also unique in that it estimates simultaneous confidence bands for curve fits, providing confidence intervals around key growth parameters as well as for the overall growth trajectory. This process is advantageous as it avoids potentially subjective retrocalculation techniques for approximating minimum ages and refrains from extrapolating a growth curve far beyond the range of the actual growth data.

Of the four model variants produced from the dataset, variant A incorporated the most data points, had the highest statistical support, and the smallest confidence band area per length. This variant included each CGM as representing an annual growth hiatus and included XPL-only annuli, resulting in the lowest peak growth rate among the four variants and the lowest maximum percent annual growth rate. The resulting sigmoid curve differs from earlier models in having a more gradual annual growth rate slope, including a protracted subadult stage, and achievement of asymptotic size approximately 15 years later than predicted by Erickson et al. (2004).

Ultimately, the growth data analyzed in this study do not directly bear on the *Nanotyrannus* or multi-taxic proposals, which remain controversial. Instead, our objective statistical methods allow us to determine that the ontogenetically immature BMRP 2006.4.4 and BMRP 2002.4.1 do not fit with other *Tyrannosaurus* specimens. However, our findings do not mean that BMRP 2006.4.4 and BMRP 2002.4.1 are identical, commensurate, or even compatible. In particular, the BMRP 2002.4.1 series is too short to conclusively say that the two specimens are mutually compatible. Therefore, determining the BMRP specimens have incompatible growth curves can only indicate that they diverge from typical *Tyrannosaurus rex* species complex growth, and cannot explain why they differ or how they should be classified.

Lastly, XPL-only annuli have implications for previous dinosaur growth modeling. In our study, the presence of XPL-only annuli frequently occurred in what are widely spaced zones when viewed in PPL. Including XPL-only annuli alongside typical annuli and LAG resulted in decreased annual growth and higher minimum ages at death of the specimens possessing them (*e.g*., the Trex2 A and NoM variants). If XPL-only annuli are indeed annual and present in dinosaur taxa for which growth curves have been previously published, those growth models would be similarly affected and reanalysis of life histories necessary.

### Addendum

We are pleased to learn that the independent study of Zanno & Napoli (2025) complements our results with growth analysis and a new specimen. Using different statistical methods and comparison data they reach the same conclusion presented here that the growth trajectories of tyrannosaur specimens BMRP 2002.4.1 and BMRP 2006.4.4 do not fit a *Tyrannosaurus rex* growth curve model. Zanno & Napoli (2025) use this result, along with anatomical evidence, to argue that the BMRP specimens are not *Tyrannosaurus* and instead should be referred to *Nanotyrannus* sp. (for BMRP 2006.4.4) and a new taxon *Nanotyrannus lethaeus* (for BMRP 2002.4.1). While we acknowledge this as a possible explanation, we do propose alternative testable hypotheses that should be considered.

Their new specimen, NCSM 40000, is referred to *Nanotyrannus lancencis*, based on both anatomical evidence and the discovery of an EFS in the femur and tibia indicating that it, unlike the BMRP specimens, had reached skeletal maturity. Zanno & Napoli (2025) use femoral CGM measurements excluding multiplets and do not report XPL lines, thus their growth series would be considered a NoXM variant in our study. Due to several methodological differences, application of our modeling methods on this specimen, including the study of CGM variants, awaits further research.

Zanno & Napoli (2025) also compare growth trajectories of NCSM 40000 and the BMRP specimens to those of two specimens referred to *Tyrannosaurus rex*, one subadult (TCMI 2003.66.1) and one fully grown (FMNH PR 2081). Our study takes a different approach by examining a larger sample of the *Tyrannosaurus rex* species complex that includes several ontogenetic stages, enabling a more comprehensive representation of growth throughout the development of the species.

## Supplemental Information

10.7717/peerj.20469/supp-1 Supplemental Information 1 Six *Tyrannosaurus* specimens did not exhibit growth marks visible only in cross polarized light (XPL). Note that the NoX variant is therefore identical to NoXM, and NoM is identical to A, so only NoXM and A variants are plotted. Cortical growth mark (CGM) count on the x-axis, CGM circumference on the y-axis.

10.7717/peerj.20469/supp-2 Supplemental Information 2 Seven *Tyrannosaurus* growth series displayed some cortical growth marks (CGM) only visible with cross polarized light (XPL). Counts for each class of CGM are in table S1. Note that Tibia CGM V33.1.15 has no multiplet CGM so its NoX variant is identical to NoXM, and A is identical to NoM. Cortical growth mark (CGM) count on the x-axes, CGM circumference on the y-axes.

10.7717/peerj.20469/supp-3 Supplemental Information 3 Individual *Tyrannosaurus* growth series for dataset Trex1, A and NoM variants. Cortical growth mark (CGM) count on the x-axis, CGM circumference on the y-axis.

10.7717/peerj.20469/supp-4 Supplemental Information 4 Individual *Tyrannosaurus* growth series for dataset Trex1, NoX and NoXM variants. Cortical growth mark (CGM) count on the x-axis, CGM circumference on the y-axis.

10.7717/peerj.20469/supp-5 Supplemental Information 5 Individual *Tyrannosaurus* growth series for dataset Trex2 (BMRP specimens removed), A and NoM variants. Cortical growth mark (CGM) count on the x-axis, CGM circumference on the y-axis.

10.7717/peerj.20469/supp-6 Supplemental Information 6 Individual *Tyrannosaurus* growth series for dataset Trex2 (BMRP specimens removed), NoX and NoXM variants. Cortical growth mark (CGM) count on the x-axis, CGM circumference on the y-axis.

10.7717/peerj.20469/supp-7 Supplemental Information 7 Individual *Gorgosaurus libratus* growth series, A and NoM variants. Data for FMNH PR 2211 obtained from Cullen et al. (2021). Cortical growth mark (CGM) count on the x-axis, CGM circumference on the y-axis.

10.7717/peerj.20469/supp-8 Supplemental Information 8 Individual *Gorgosaurus libratus* growth series, A and NoM variants. Data for FMNH PR 2211 obtained from Cullen et al. (2021). Cortical growth mark (CGM) count on the x-axis, CGM circumference on the y-axis.

10.7717/peerj.20469/supp-9 Supplemental Information 9 The first step of least squares clustering is projecting overlapping growth series onto one another. CGM growth series are said to overlap if the range of circumferences for the series overlap. In that case the (*cgmci, sizei*) points for each overlapping series are “projected” onto all the other overlapping series. Dashed arrows show the projections. All are calculated assuming piecewise linear interpolation between the (*cgmci, sizei*) points of the series being projected upon. The least squares clustering algorithm then minimizes the sum of the square of the projection lengths (*i.e*. length of the arrows) for all overlaps, varying the starting age of each growth series. Cortical growth mark (CGM) count on the x-axis, CGM circumference on the y-axis.

10.7717/peerj.20469/supp-10 Supplemental Information 10 *Tyrannosaurus* dataset Trex1 growth series clustered by least squares, for A and NoM variants. For each specimen, the starting cortical growth mark (CGM) age is estimated using least squares minimization, and the numerical values are found in the inset table in the column labeled “1st”. All ages are in years relative to the starting age of the smallest CGM circumference in Tibia MOR 1189.

10.7717/peerj.20469/supp-11 Supplemental Information 11 *Tyrannosaurus* dataset Trex1 growth series clustered by least squares, for NoX and NoXM variants. For each specimen, the starting cortical growth mark (CGM) age is estimated using least squares minimization, and the numerical values are found in the inset table in the column labeled “1st”. All ages are in years relative to the starting age of the smallest CGM circumference in Tibia MOR 1189.

10.7717/peerj.20469/supp-12 Supplemental Information 12 *Tyrannosaurus* dataset Trex2 growth series clustered by least squares, for A and NoM variants. . For each specimen, the starting cortical growth mark (CGM) age is estimated using least squares minimization, and the numerical values are found in the inset table in the column labeled “1st”. All ages are in years relative to the starting age of the smallest CGM circumference in Tibia MOR 1189.

10.7717/peerj.20469/supp-13 Supplemental Information 13 *Tyrannosaurus* dataset Trex2 growth series clustered by least squares, for NoX and NoXM variants. For each specimen, the starting cortical growth mark (CGM) age is estimated using least squares minimization, and the numerical values are found in the inset table in the column labeled “1st”. All ages are in years relative to the starting age of the smallest CGM circumference in Tibia MOR 1189.

10.7717/peerj.20469/supp-14 Supplemental Information 14 *Tyrannosaurus* growth modeling for dataset Trex1, A and NoM variants. The 95% simultaneous confidence bands (CB) and best fit equations are shown, in this case the arctan 3 function for variant A and the laplace 3 function for variant NoM. All ages are in years relative to the starting age of the smallest cortical growth mark (CGM) circumference in Tibia MOR 1189.

10.7717/peerj.20469/supp-15 Supplemental Information 15 *Tyrannosaurus* growth modeling for dataset Trex1, NoX and NoXM variants. The 95% simultaneous confidence bands (CB) and best fit equations are shown, in this case the laplace 3 function for variant NoX and the arctan 3 function for variant NoXM. All ages are in years relative to the starting age of the smallest cortical growth mark (CGM) circumference in Tibia MOR 1189.

10.7717/peerj.20469/supp-16 Supplemental Information 16 *Tyrannosaurus* growth modeling for dataset Trex2, A and NoM variants. The 95% simultaneous confidence bands (CB) and best fit equations are shown, in this case the extreme value 3 function for both variants. All ages are in years relative to the starting age of the smallest cortical growth mark (CGM) circumference in Tibia MOR 1189.

10.7717/peerj.20469/supp-17 Supplemental Information 17 *Tyrannosaurus* growth modeling for dataset Trex2, NoX and NoXM variants. The 95% simultaneous confidence bands (CB) and best fit equations are shown, in this case the extreme value 3 function for both variants. All ages are in years relative to the starting age of the smallest cortical growth mark (CGM) circumference in Tibia MOR 1189.

10.7717/peerj.20469/supp-18 Supplemental Information 18 Residuals to best fitting growth models for variants of Trex2 dataset. The residuals from the data series are plotted, along with their 0.025, 0.5 (median) and 0.975 quantiles.

10.7717/peerj.20469/supp-19 Supplemental Information 19 Comparison of arithmetic and log-transformed growth models for Trex2, A and NoM variants. All ages are in years relative to the starting age of the smallest cortical growth mark (CGM) circumference in Tibia MOR 1189.

10.7717/peerj.20469/supp-20 Supplemental Information 20 Comparison of arithmetic and log-transformed growth models for Trex2, NoX and NoXM variants. All ages are in years relative to the starting age of the smallest cortical growth mark (CGM) circumference in Tibia MOR 1189.

10.7717/peerj.20469/supp-21 Supplemental Information 21 Synthetic linear growth series for testing compatibility. . Each panel shows a different variant (A, NoM, NoX, NoXM). The black growth series is Tibia BMRP 2006.4.4. The colored lines show linear growth series of the same length, but with constant linear growth rate ranging from 2 mm/year (bottom most line) to 30 mm/year (topmost line). Cortial growth mark (CGM) count on the x-axes, CGM circumference on the y-axes.

10.7717/peerj.20469/supp-22 Supplemental Information 22 Confidence band (CB) area and CB area/length for trials with synthetic growth series at different linear growth rates. The plots show the total CB area (top panel) and CB area divided by length of CB (bottom panel) for different variants of the Trex2 dataset, plus one synthetic data series with linear growth at the specified rate. Each area (or area/length) curve reaches a minimum value at the black points. Minimum values indicate the linear growth rate that is the most compatible (of those simulated) with the given variant of the Trex2 data series.

10.7717/peerj.20469/supp-23 Supplemental Information 23 BMRP 2002.4.1 and BMRP 2006.4.4 growth series for each variant. Cortical growth mark (CGM) count on the x-axes, CGM circumference (mm) on the y-axes.

10.7717/peerj.20469/supp-24 Supplemental Information 24 BMRP 2002.4.1 and BMRP 2006.4.4 growth series annotated. The A variant of the growth series contains all of the observed cortical growth marks (CGM). CGM marked M are multiplets which would not be present in the NoM, or NoMX variants. Those marked X are XPL CGM and would be present in the NoX and NoXM variants.

10.7717/peerj.20469/supp-25 Supplemental Information 25 Specimen raw data. Individual specimen tabs provide raw data tables for each growth series.

10.7717/peerj.20469/supp-26 Supplemental Information 26 Count of cortical growth marks (CGM) visible in cross polarized light (XPL) and those comprising a multiplet (M). CGM which are only visible in cross polarized light are counted in the column XPL count. CGM which are omitted from variants NoM and NoXM on the basis that they are members of multiplets are counted in column “M count”.

10.7717/peerj.20469/supp-27 Supplemental Information 27 Arithmetic scale models fit to growth data. This table is primarily composed of sigmoidal models, but simple linear and quadratic models are also included. For the sigmoidal models the parameter is always the maximum asymptotic size, is always a location parameter that determines where on the time axis the sigmoid is located. The parameter is related to the slope (growth rate) of the model in its intermediate growth phase.

10.7717/peerj.20469/supp-28 Supplemental Information 28 Models fit to log-transformed growth data. The models fit to conventional (arithmetic scale) data are modified for fitting to log-transformed data, in which the circumference values are log-transformed. Here we use log to mean natural logarithm. Parameter interpretation is as described for arithmetic scale models. The Laplace 3 model (Table S2) is not included because it has algorithmic issues in fitting.

10.7717/peerj.20469/supp-29 Supplemental Information 29 Trex2 model parameters and their 95% confidence intervals (CI). The model, parameter values and 95% CI for the parameter values are shown in the table, with the CI expressed both in a high/low, and as a percentage variation on the median value. The parameters are for the sigmoidal model shown. The parameters for each of the specimen names are the estimated ages to be added to the 1 st CGM in the growth series for the specimen (in years).

10.7717/peerj.20469/supp-30 Supplemental Information 30 Trex2 model parameters and their 95% confidence intervals (CI). The model, parameter values and 95% CI for the parameter values are shown in the table, with the CI expressed both in a high/low, and as a percentage variation on the median value. The parameters are for the sigmoidal model shown. The parameters for each of the specimen names are the estimated age to be added to the 1 st CGM in the growth series for the specimen (in years).

## Institutional abbreviations

BDM: Badlands Dinosaur Museum
BMRP: Burpee Museum of Natural History
CCM: Carter County Museum
CMNH: Cleveland Museum of Natural History
DDM: Dinosaur Discovery Museum
FMNH: Field Museum of Natural History
MOR: Museum of the Rockies
RSM: Royal Saskatchewan Museum
TMP: Royal Tyrrell Museum
USNM: Smithsonian National Museum of Natural History

## Additional Information and Declarations

### Data Availability

The following information was supplied regarding data availability:

The raw data, data tables and additional graphs are available in the Supplemental Files.

The raw transverse thin section images and growth mark tracings are available at Figshare, Woodward Ballard, Holly; Myhrvold, Nathan P.; Horner, John R. (2025). Tyrannosaur thin section images and associated cortical growth mark tracings for Woodward et al. (2025) Prolonged growth and extended subadult development in the Tyrannosaurus rex species complex revealed by expanded histological sampling and statistical modeling. figshare. Figure. https://doi.org/10.6084/m9.figshare.29944892.v1
