# Supplementary figures and images for: Prolonged growth and extended subadult development in the Tyrannosaurus rex species complex revealed by expanded histological sampling and statistical modeling
Source: PeerJ. 2026 Jan 14;14:e20469. doi: 10.7717/peerj.20469 (PMC12811967; doi:10.7717/peerj.20469)

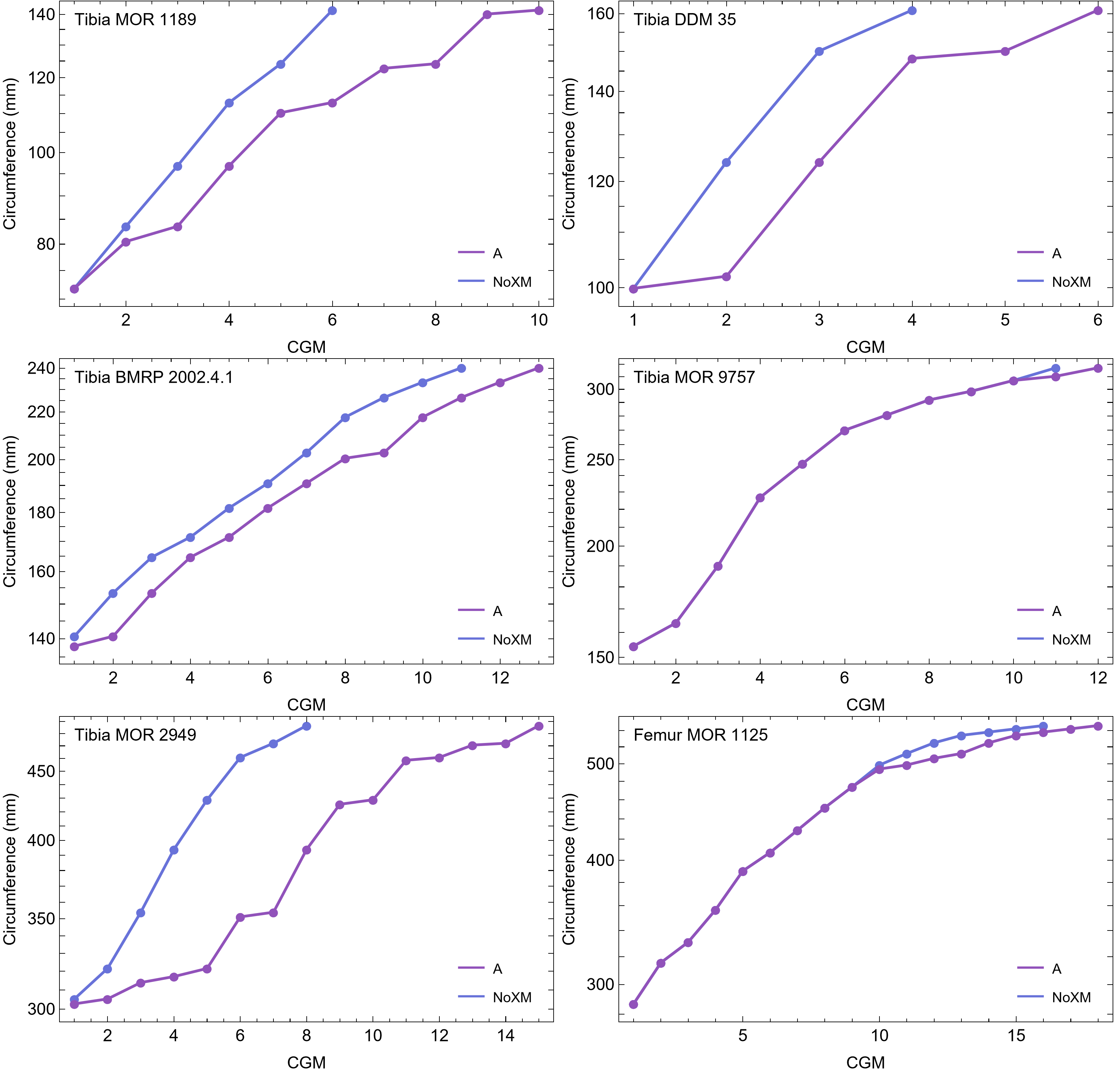

Supplement: Supplemental Information 1 — Note that the NoX variant is therefore identical to NoXM, and NoM is identical to A, so only NoXM and A variants are plotted. Cortical growth mark (CGM) count on the x-axis, CGM circumference on the y-axis. [file peerj-14-20469-s001.png]

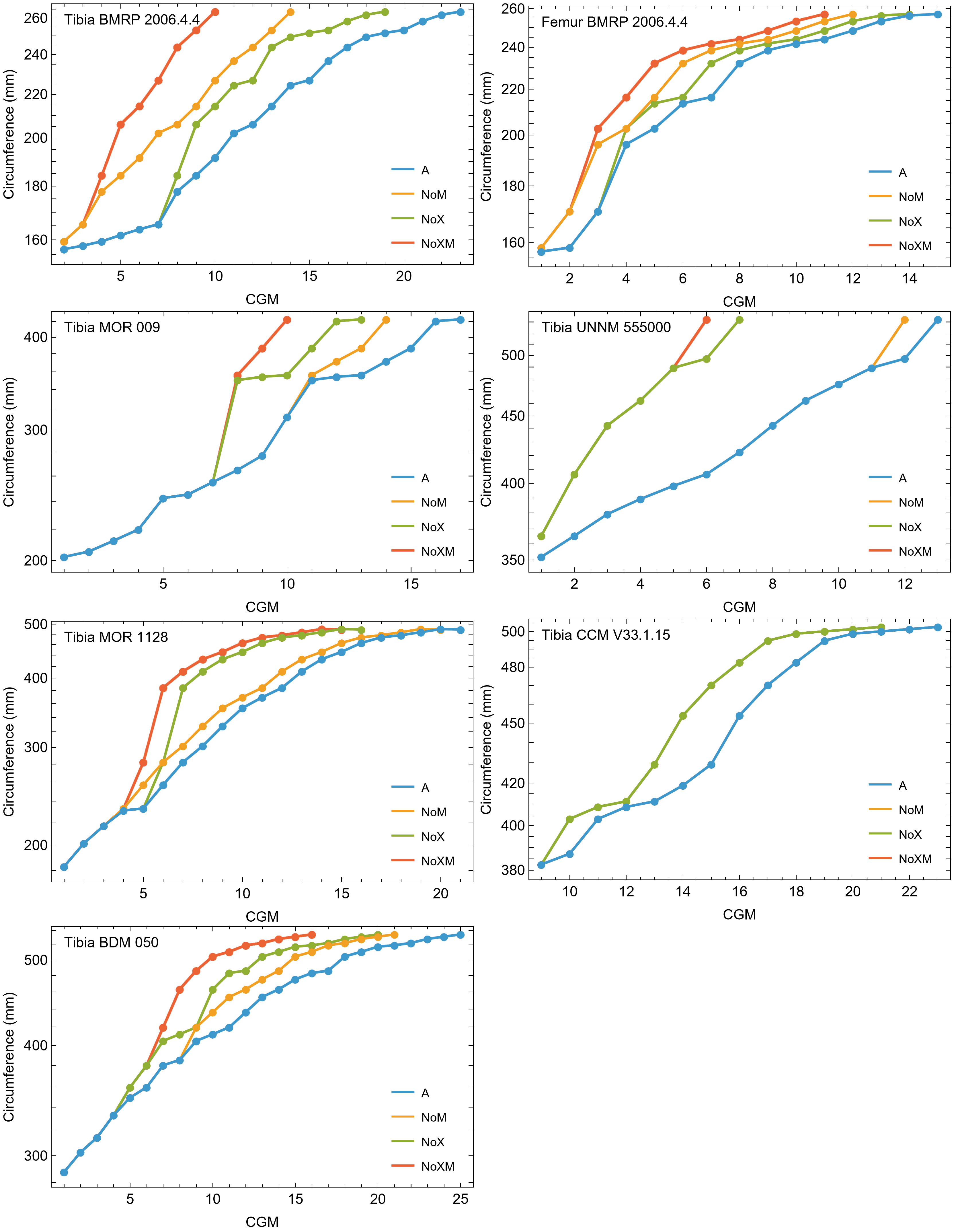

Supplement: Supplemental Information 2 — Counts for each class of CGM are in table S1. Note that Tibia CGM V33.1.15 has no multiplet CGM so its NoX variant is identical to NoXM, and A is identical to NoM. Cortical growth mark (CGM) count on the x-axes, CGM circumference on the y-axes. [file peerj-14-20469-s002.png]

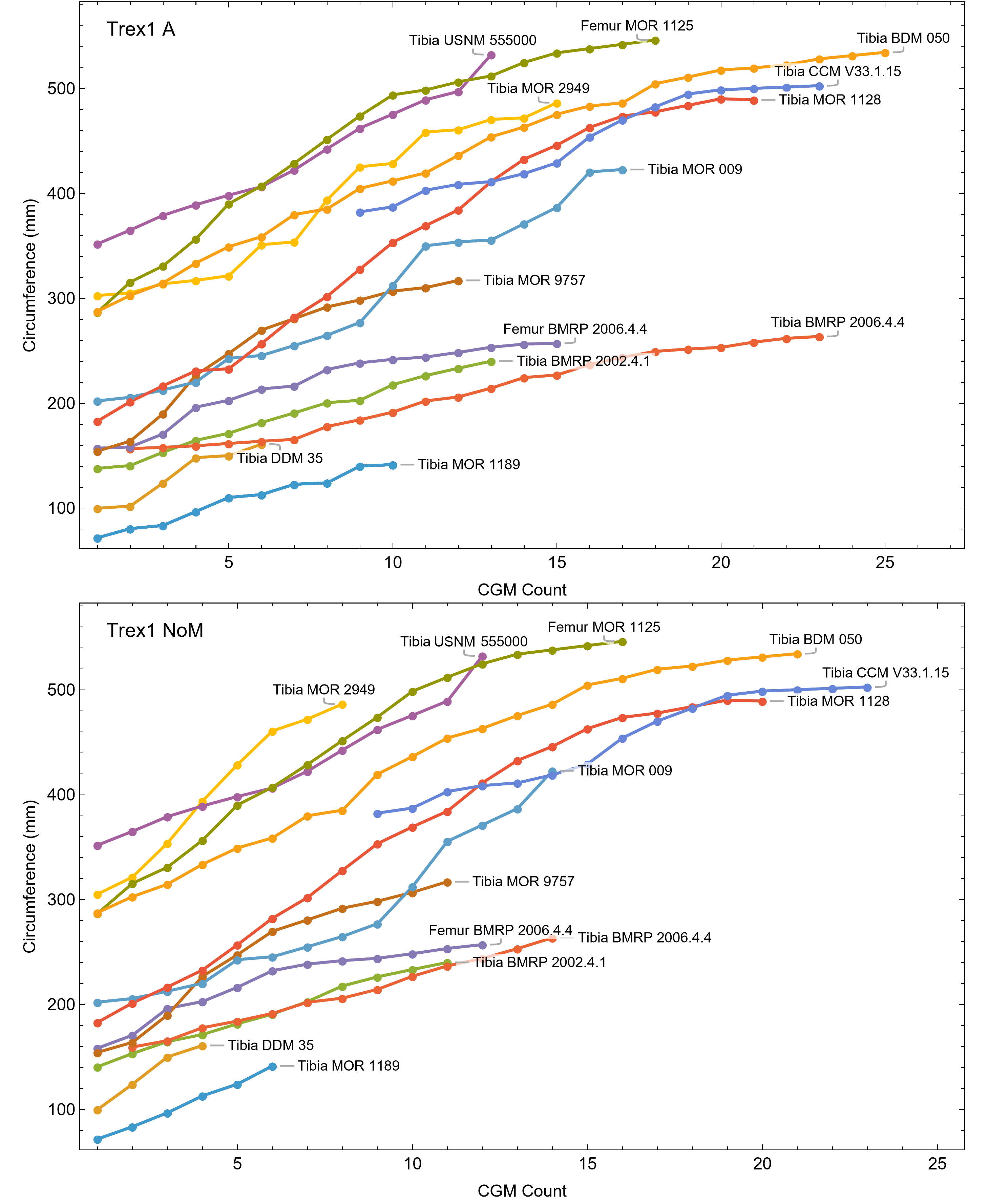

Supplement: Supplemental Information 3 — Cortical growth mark (CGM) count on the x-axis, CGM circumference on the y-axis. [file peerj-14-20469-s003.png]

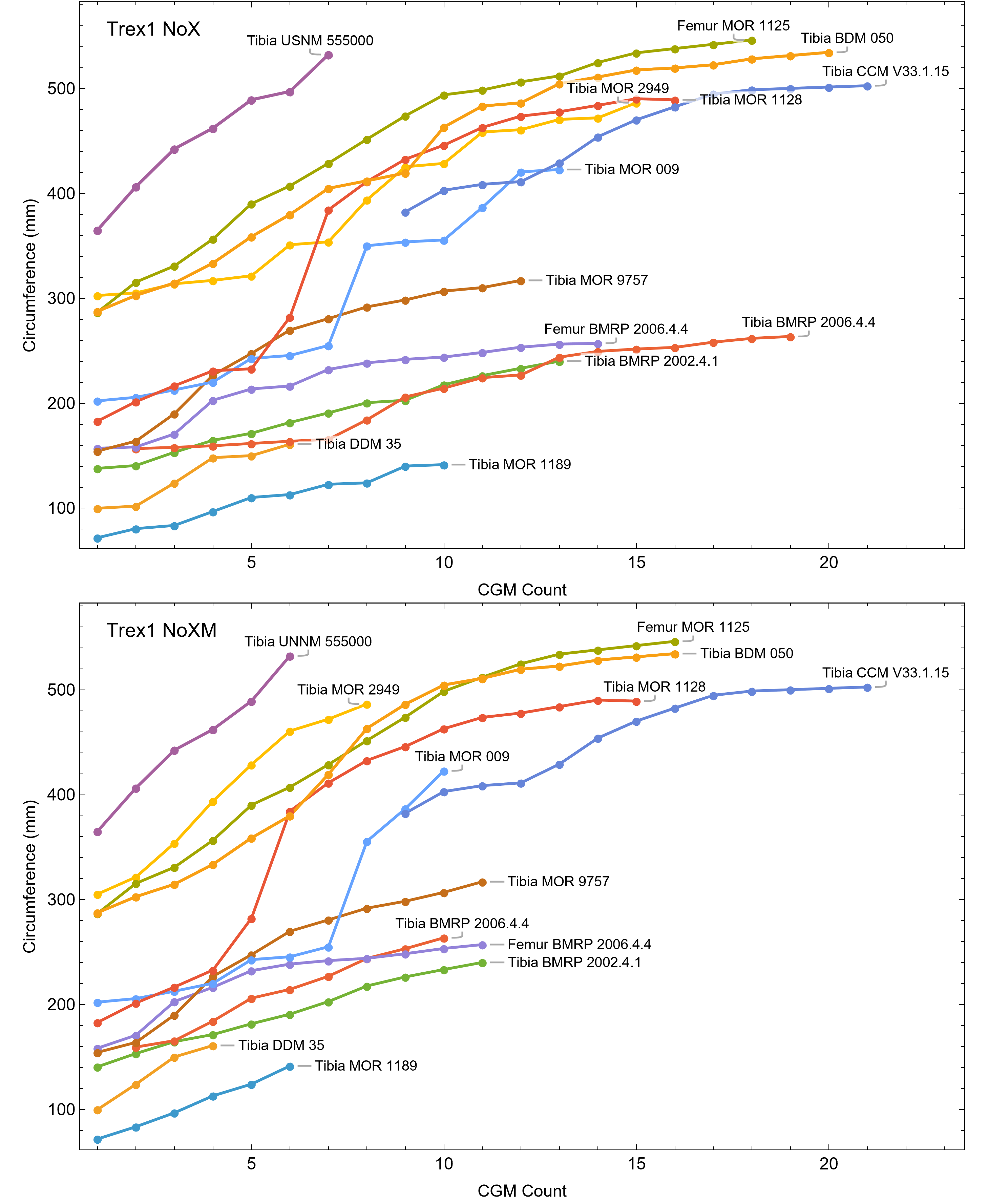

Supplement: Supplemental Information 4 — Cortical growth mark (CGM) count on the x-axis, CGM circumference on the y-axis. [file peerj-14-20469-s004.png]

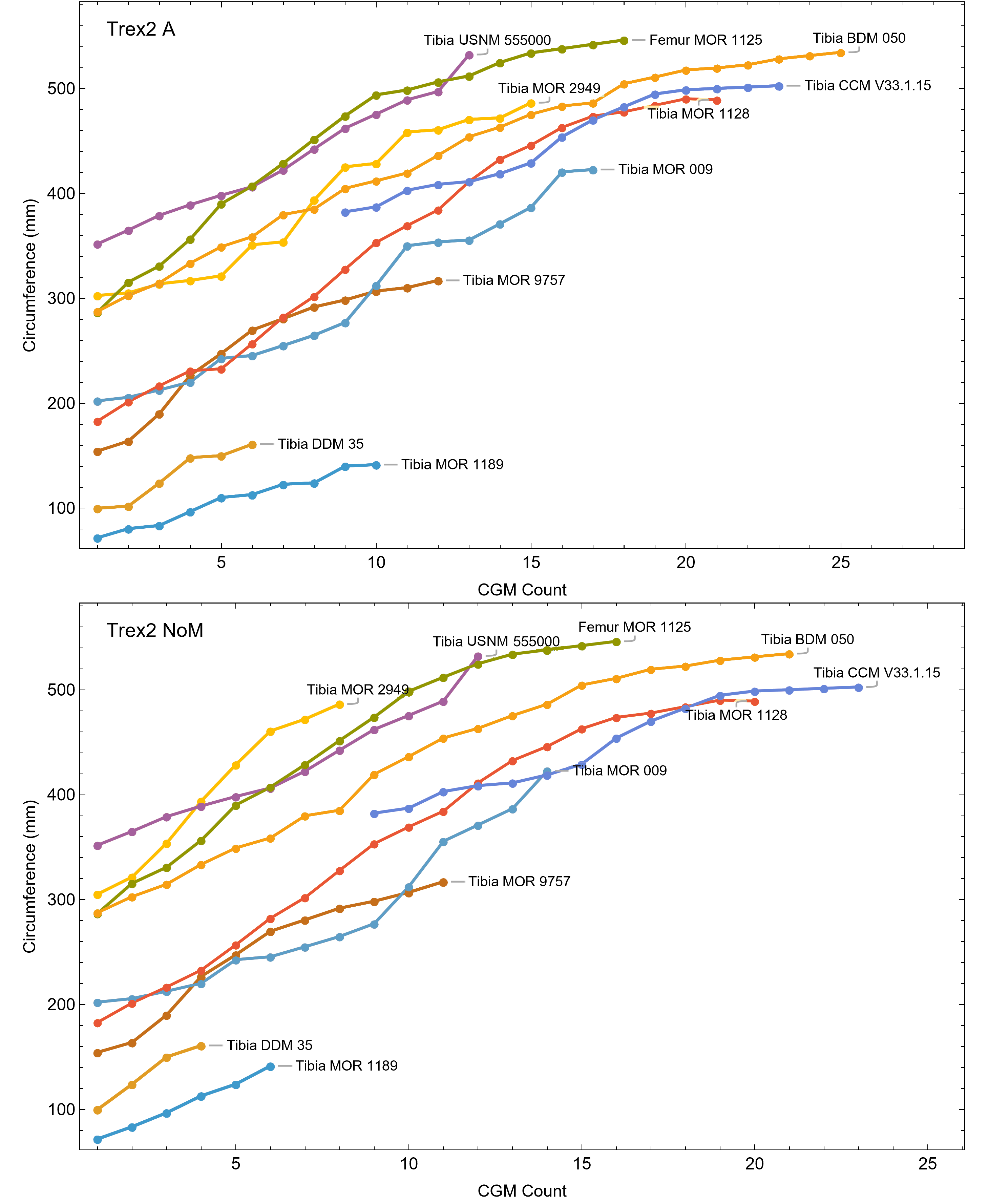

Supplement: Supplemental Information 5 — Cortical growth mark (CGM) count on the x-axis, CGM circumference on the y-axis. [file peerj-14-20469-s005.png]

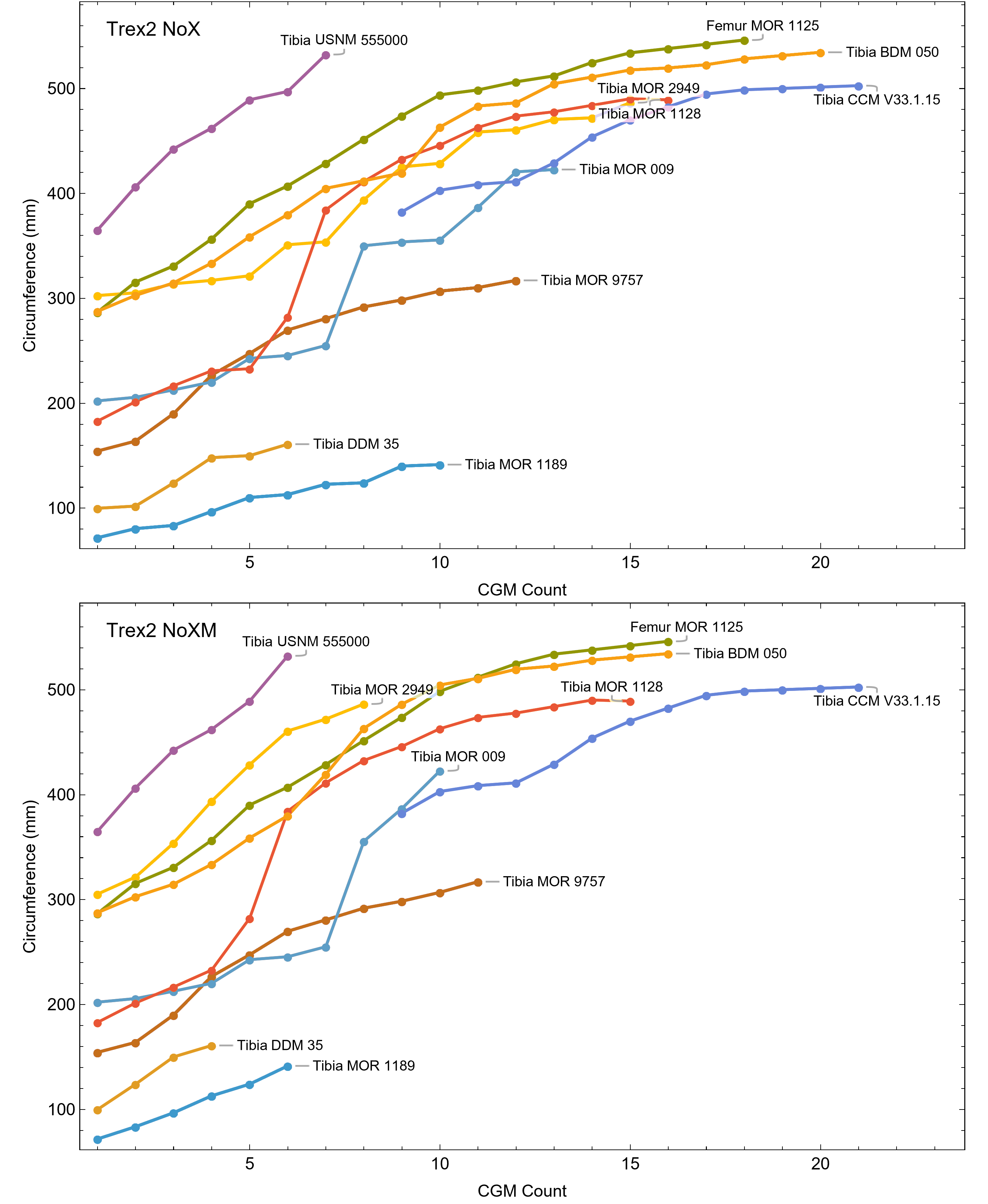

Supplement: Supplemental Information 6 — Cortical growth mark (CGM) count on the x-axis, CGM circumference on the y-axis. [file peerj-14-20469-s006.png]

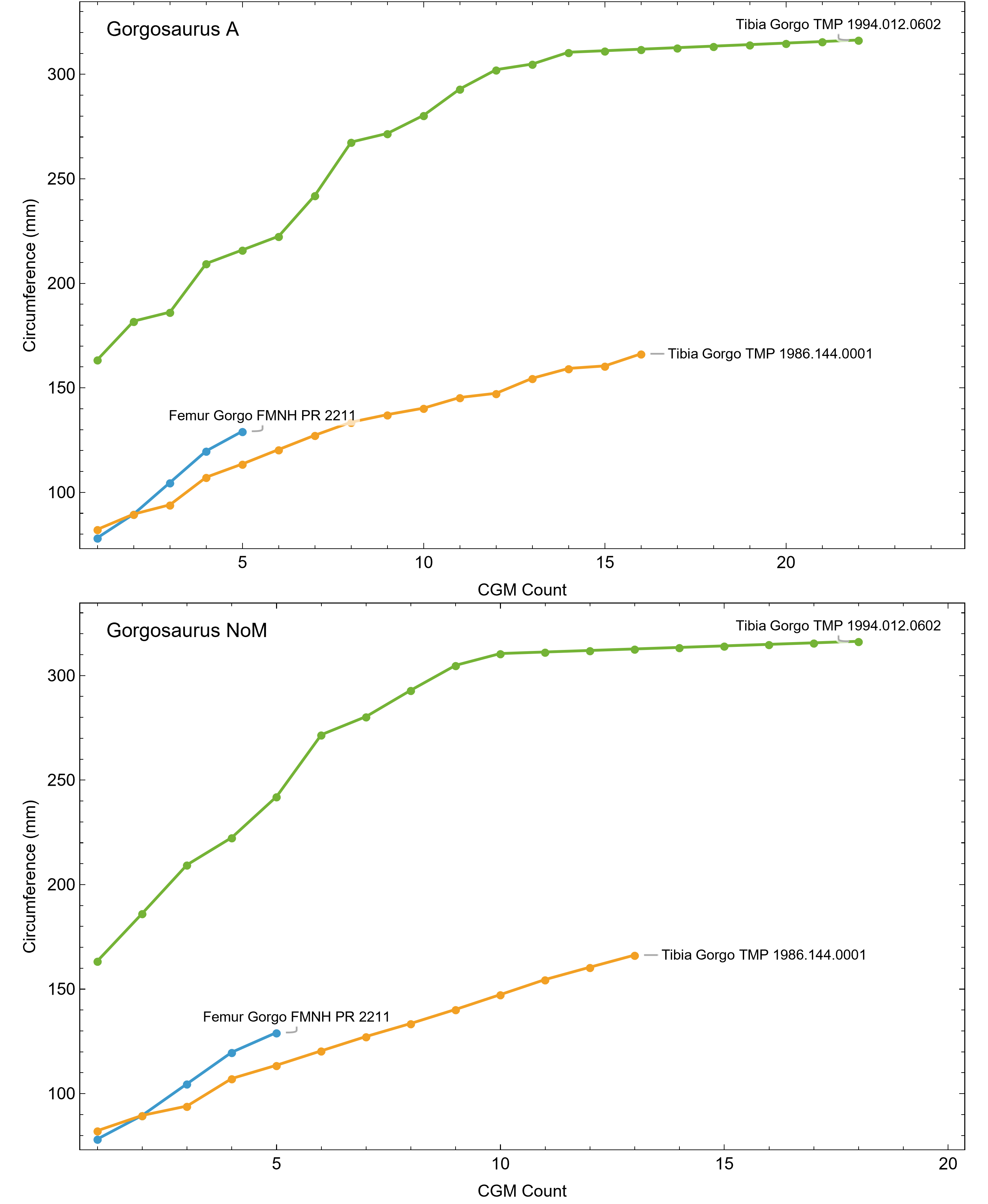

Supplement: Supplemental Information 7 — Data for FMNH PR 2211 obtained from Cullen et al. (2021). Cortical growth mark (CGM) count on the x-axis, CGM circumference on the y-axis. [file peerj-14-20469-s007.png]

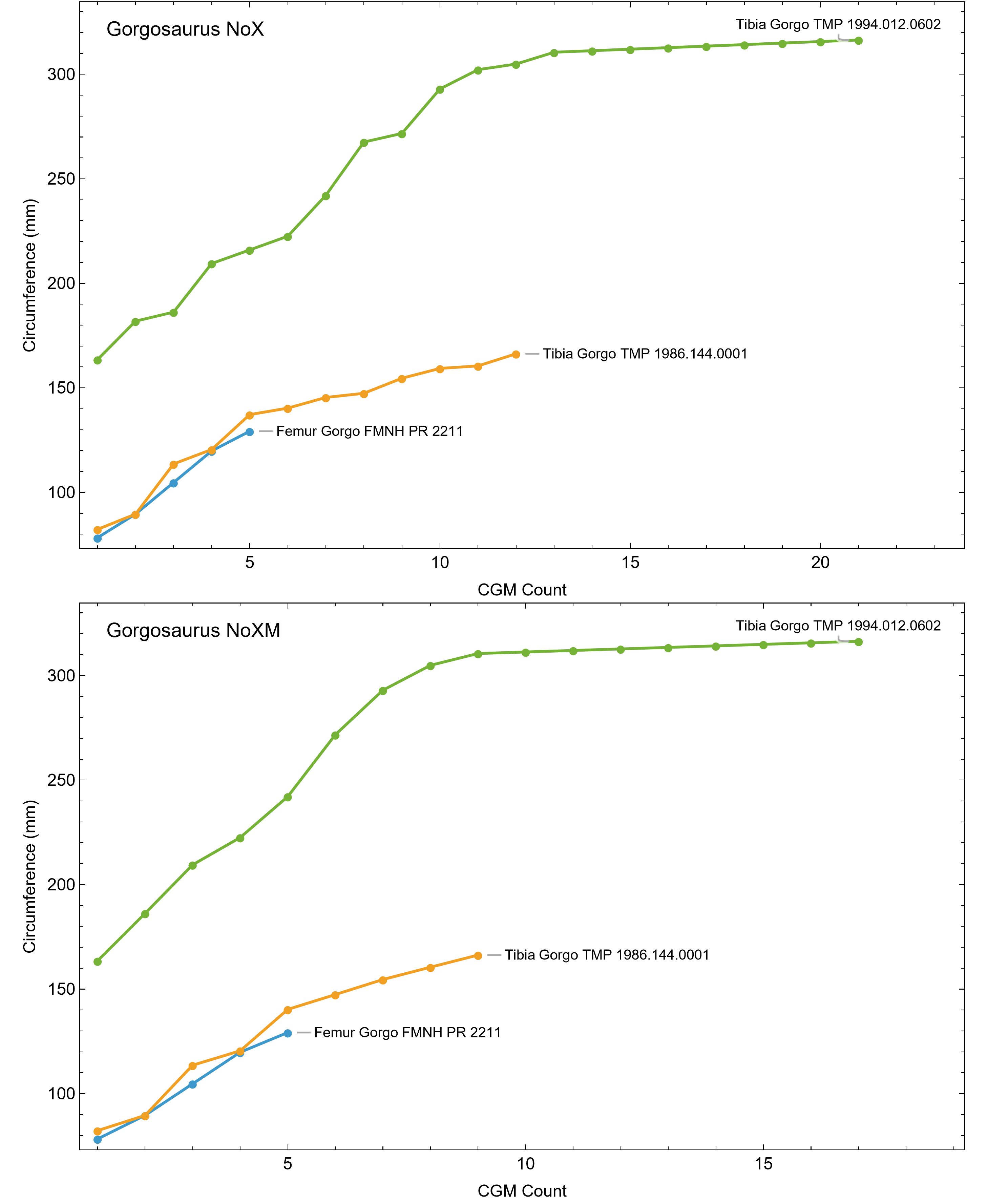

Supplement: Supplemental Information 8 — Data for FMNH PR 2211 obtained from Cullen et al. (2021). Cortical growth mark (CGM) count on the x-axis, CGM circumference on the y-axis. [file peerj-14-20469-s008.png]

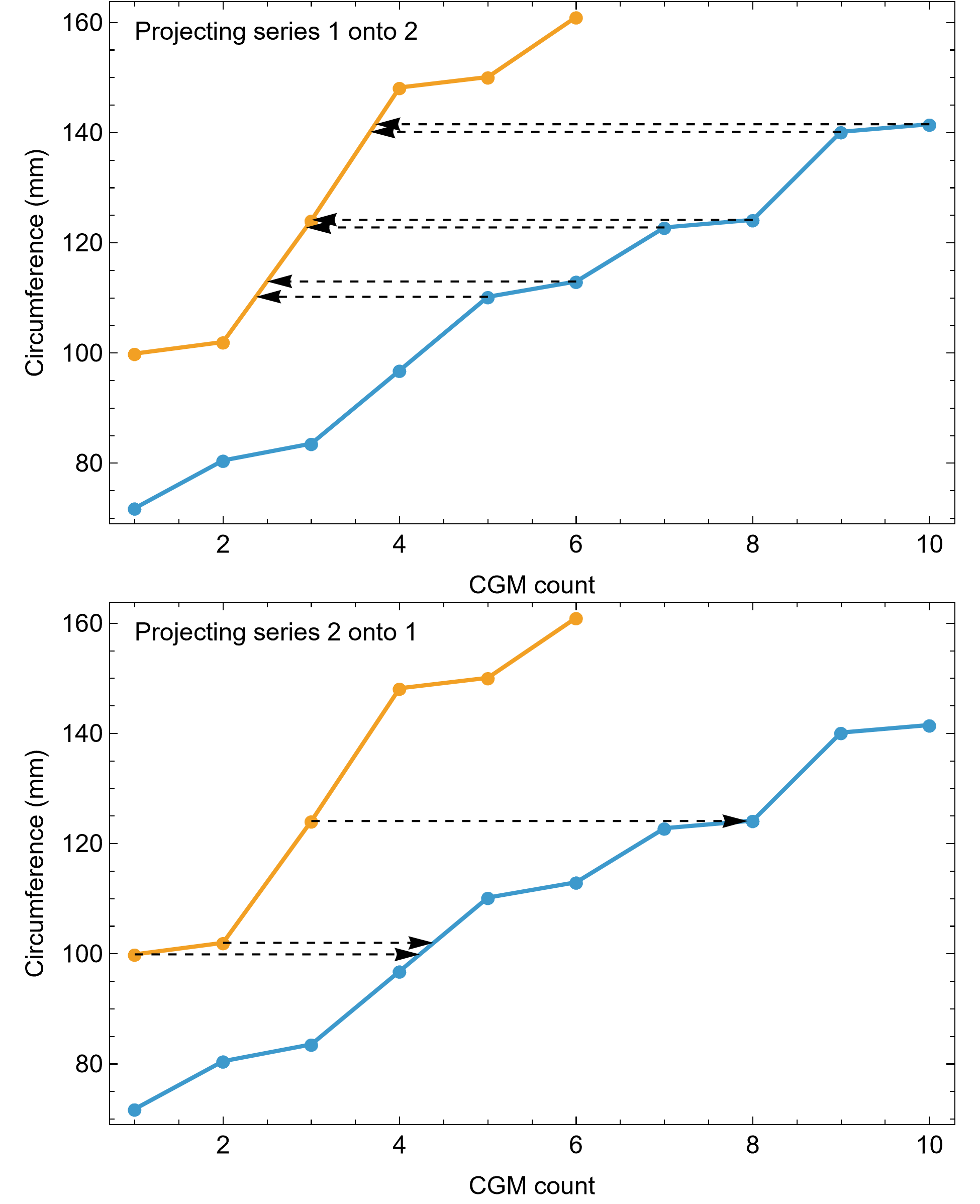

Supplement: Supplemental Information 9 — CGM growth series are said to overlap if the range of circumferences for the series overlap. In that case the (cgmci, sizei) points for each overlapping series are “projected” onto all the other overlapping series. Dashed arrows show the projections. All are calculated assuming piecewise linear interpolation between the (cgmci, sizei) points of the series being projected upon. The least squares clustering algorithm then minimizes the sum of the square of the projection lengths (i.e. length of the arrows) for all overlaps, varying the starting age of each growth series. Cortical growth mark (CGM) count on the x-axis, CGM circumference on the y-axis. [file peerj-14-20469-s009.png]

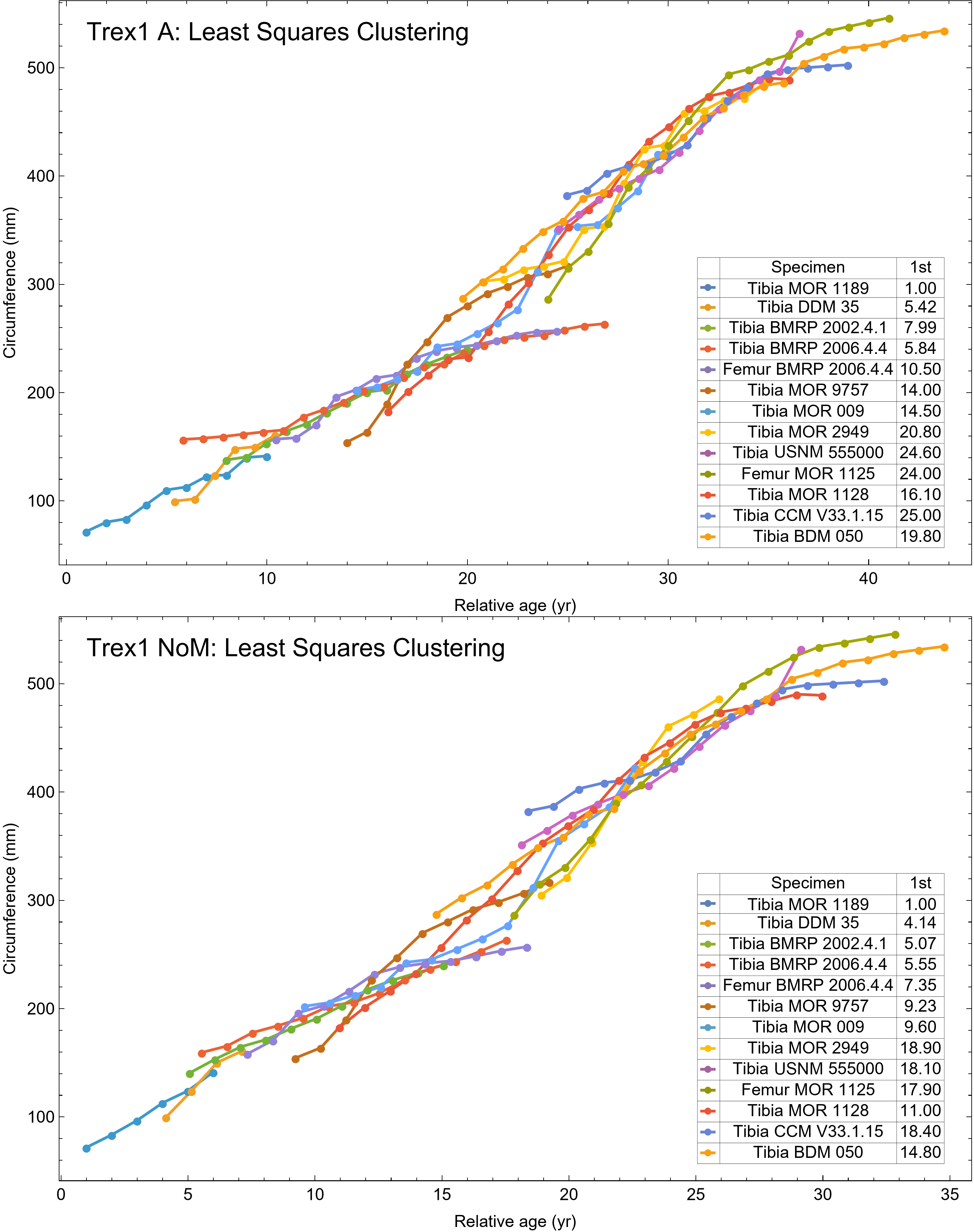

Supplement: Supplemental Information 10 — For each specimen, the starting cortical growth mark (CGM) age is estimated using least squares minimization, and the numerical values are found in the inset table in the column labeled “1st”. All ages are in years relative to the starting age of the smallest CGM circumference in Tibia MOR 1189. [file peerj-14-20469-s010.png]

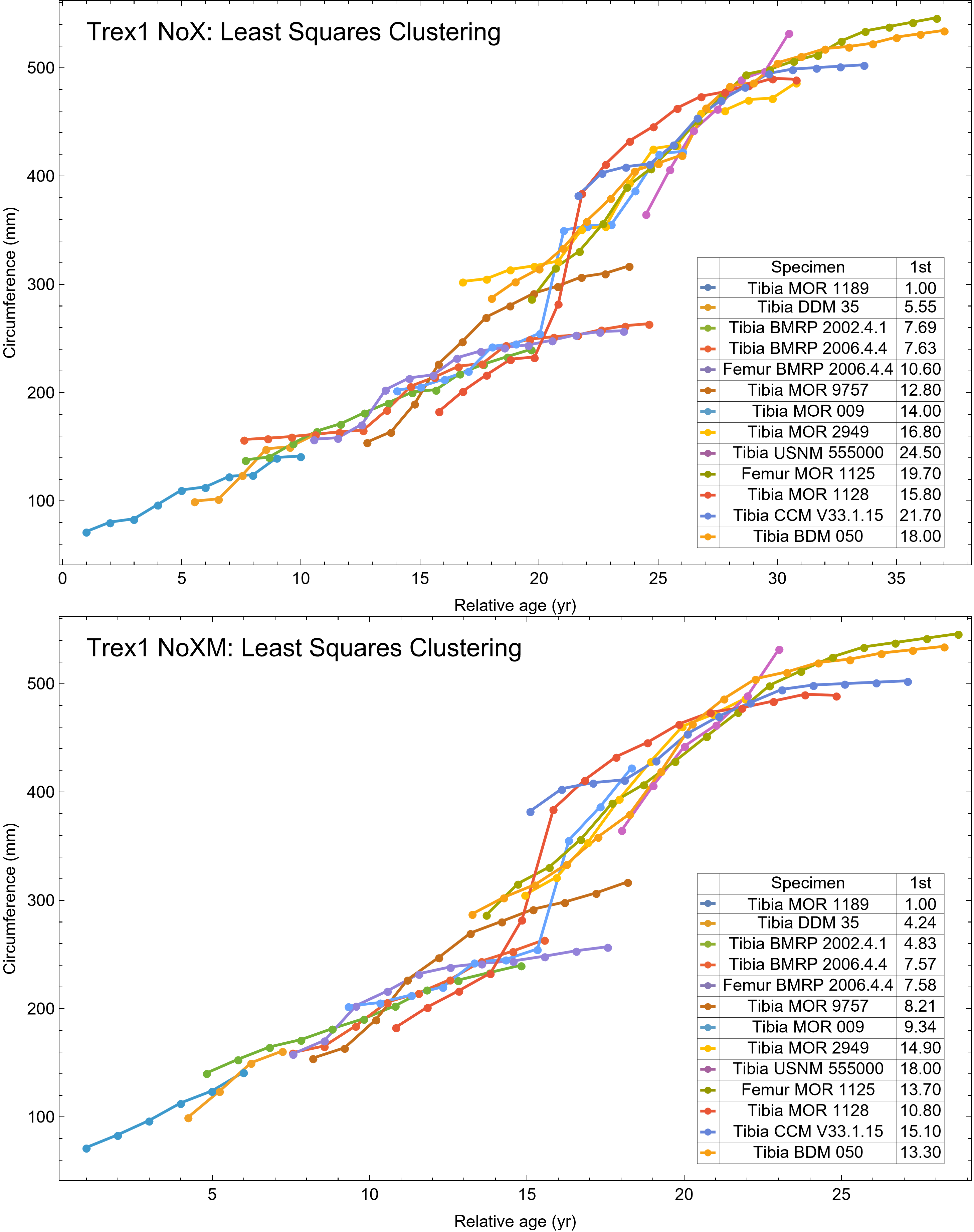

Supplement: Supplemental Information 11 — For each specimen, the starting cortical growth mark (CGM) age is estimated using least squares minimization, and the numerical values are found in the inset table in the column labeled “1st”. All ages are in years relative to the starting age of the smallest CGM circumference in Tibia MOR 1189. [file peerj-14-20469-s011.png]

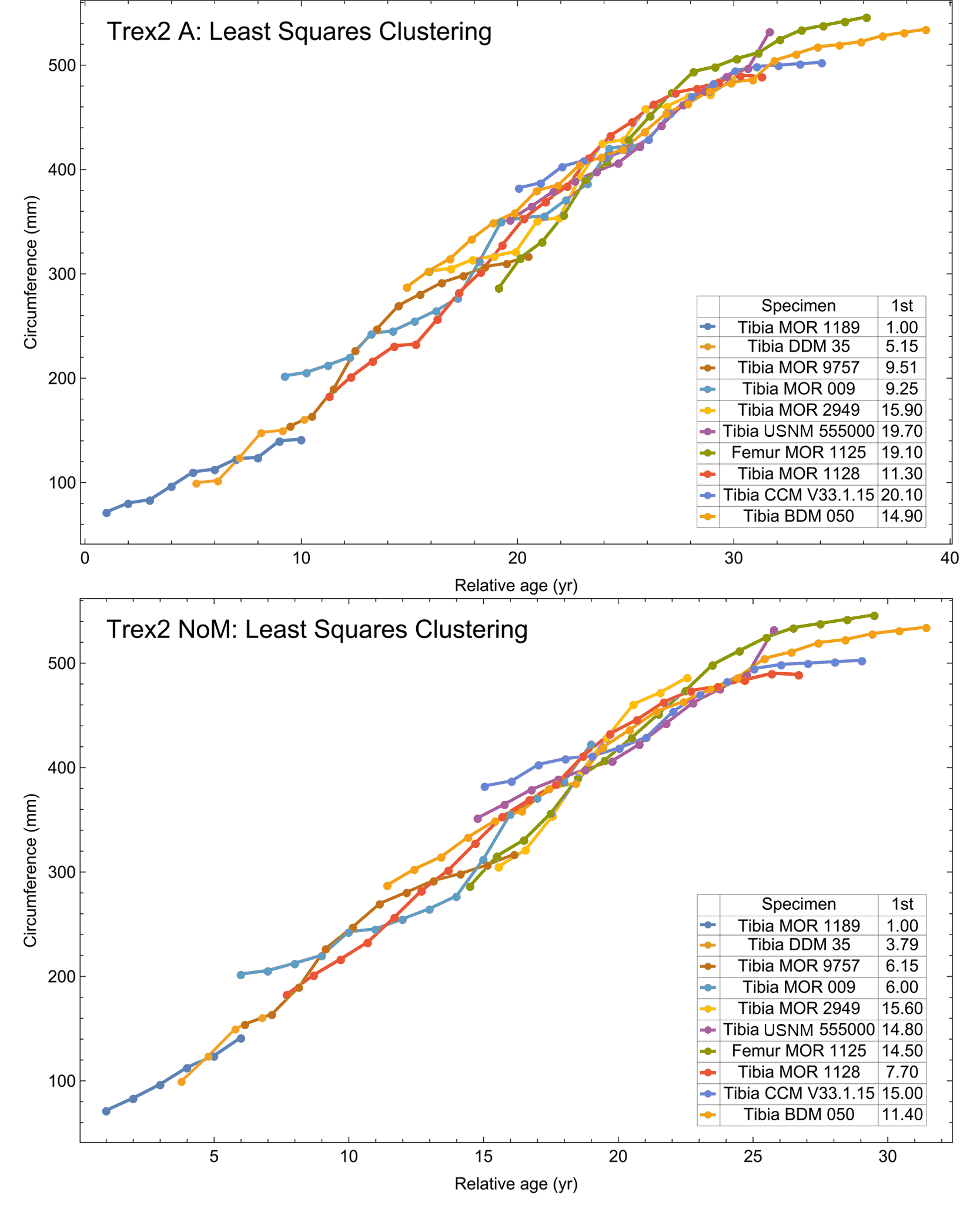

Supplement: Supplemental Information 12 — . For each specimen, the starting cortical growth mark (CGM) age is estimated using least squares minimization, and the numerical values are found in the inset table in the column labeled “1st”. All ages are in years relative to the starting age of the smallest CGM circumference in Tibia MOR 1189. [file peerj-14-20469-s012.png]

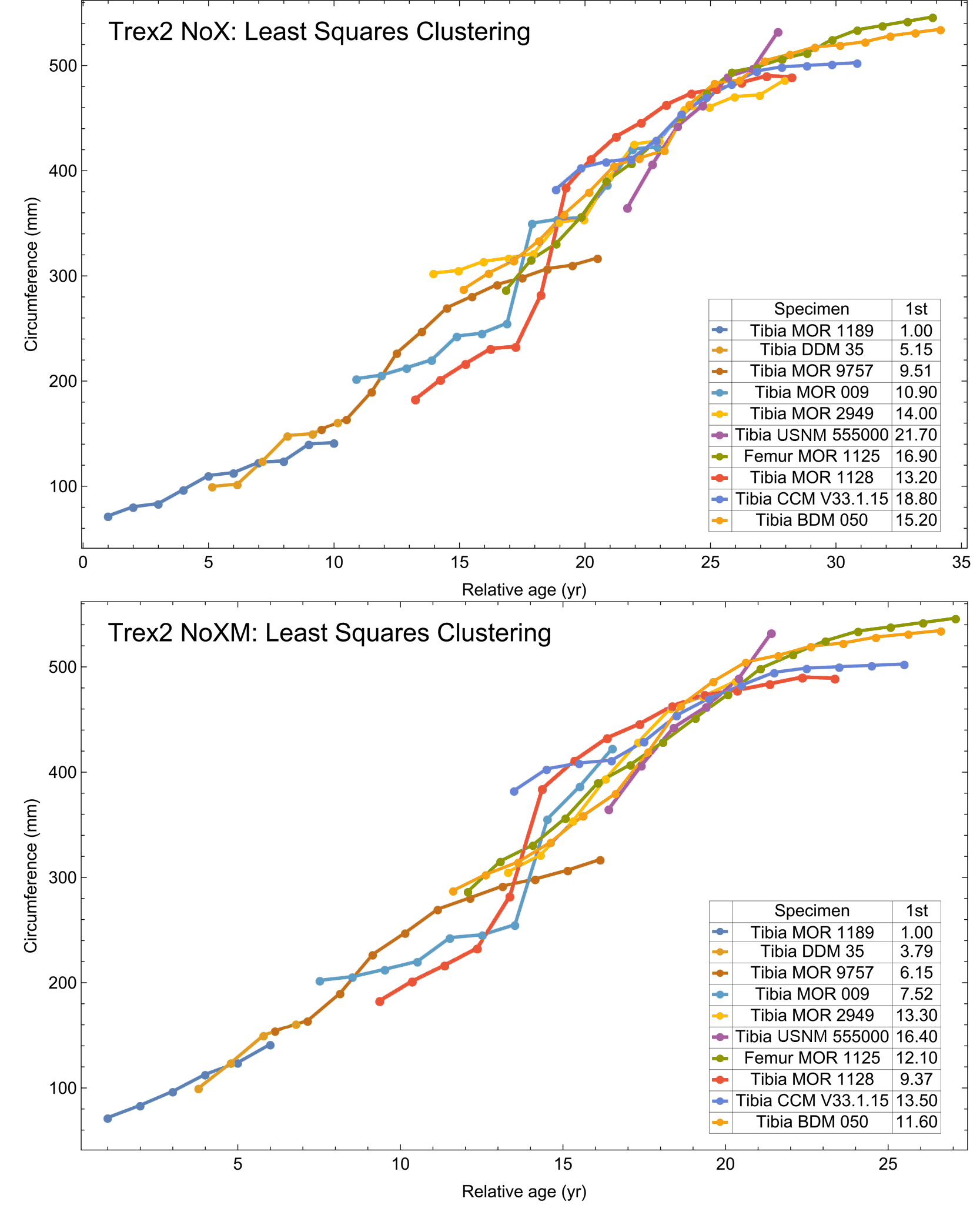

Supplement: Supplemental Information 13 — For each specimen, the starting cortical growth mark (CGM) age is estimated using least squares minimization, and the numerical values are found in the inset table in the column labeled “1st”. All ages are in years relative to the starting age of the smallest CGM circumference in Tibia MOR 1189. [file peerj-14-20469-s013.png]

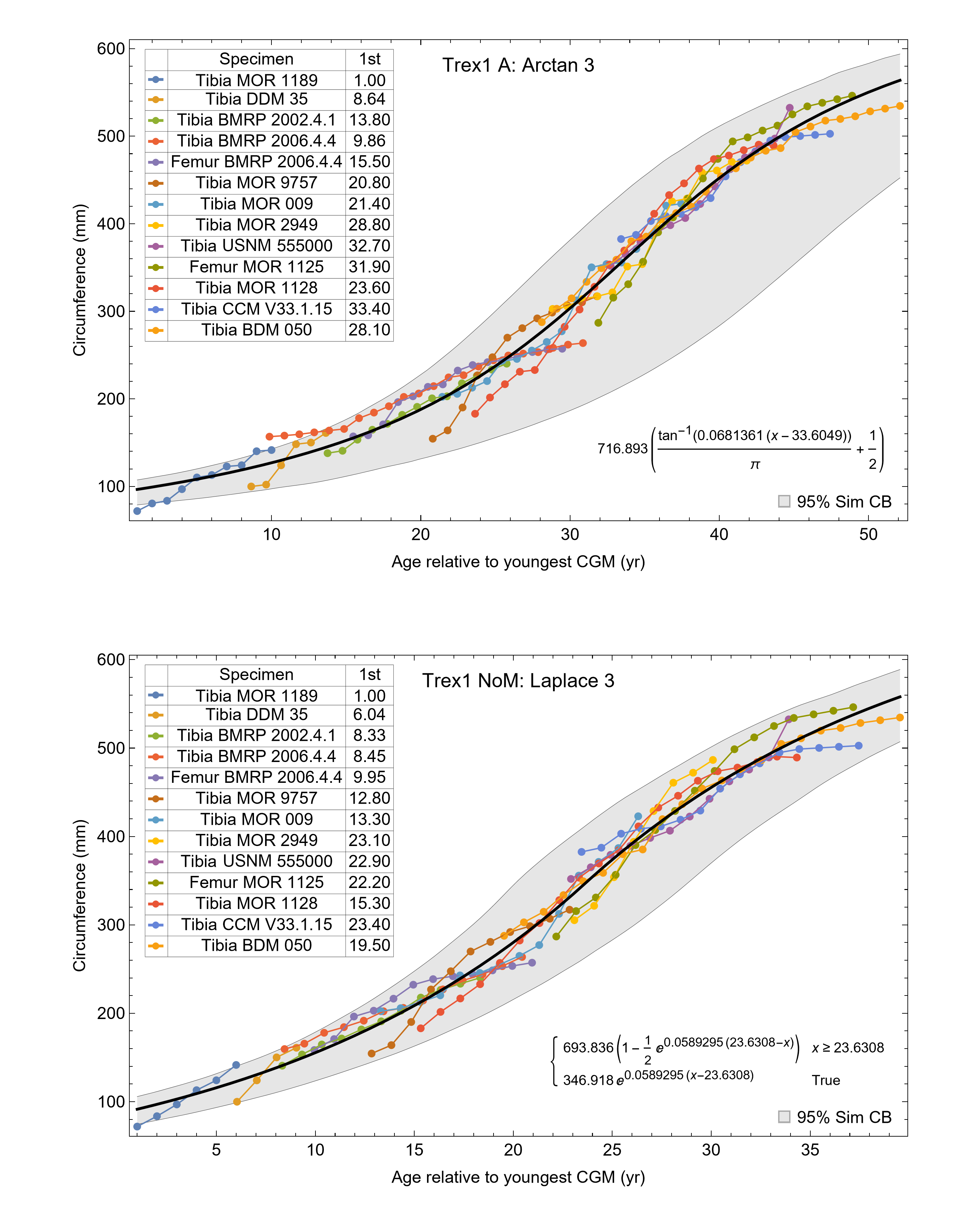

Supplement: Supplemental Information 14 — The 95% simultaneous confidence bands (CB) and best fit equations are shown, in this case the arctan 3 function for variant A and the laplace 3 function for variant NoM. All ages are in years relative to the starting age of the smallest cortical growth mark (CGM) circumference in Tibia MOR 1189. [file peerj-14-20469-s014.png]

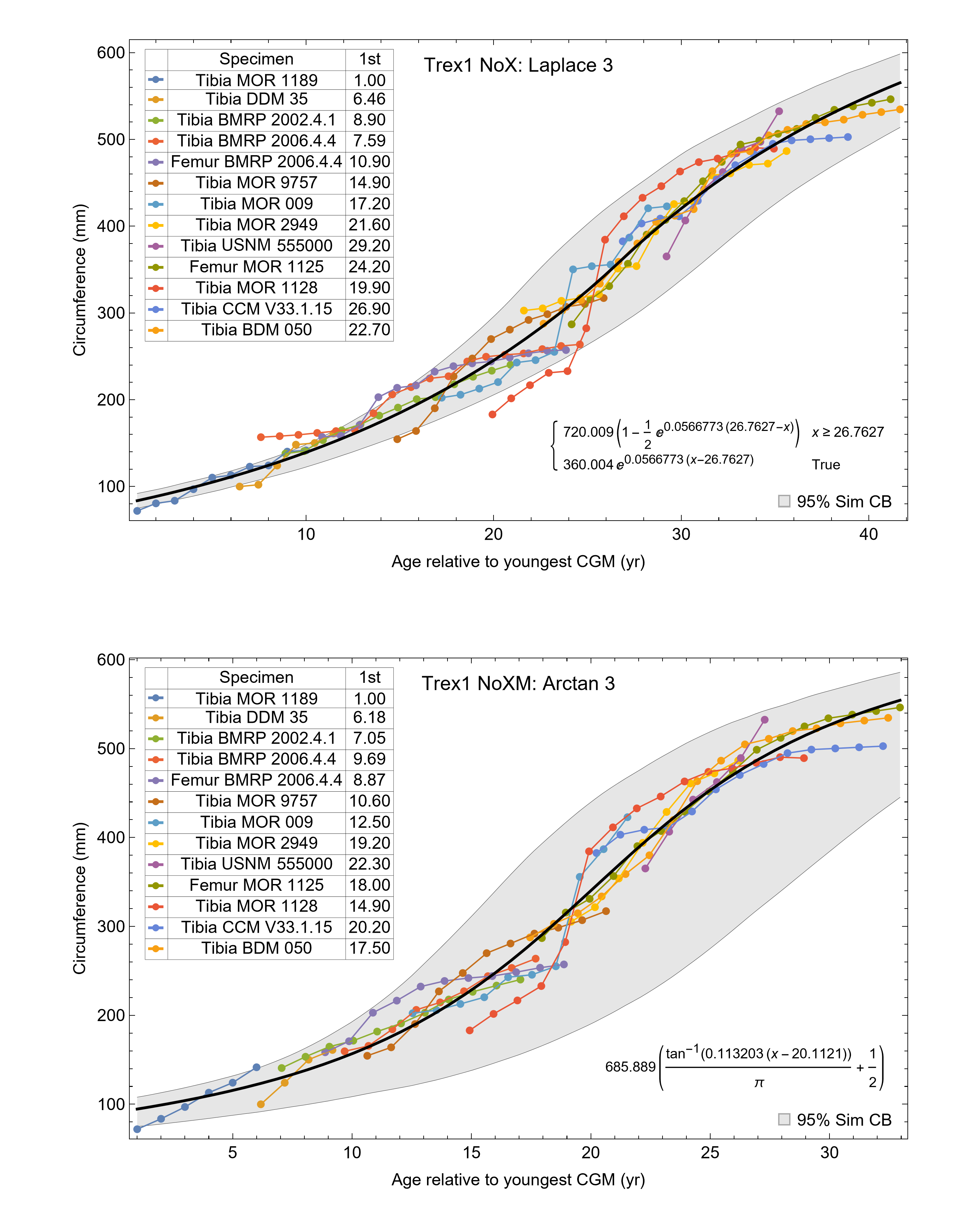

Supplement: Supplemental Information 15 — The 95% simultaneous confidence bands (CB) and best fit equations are shown, in this case the laplace 3 function for variant NoX and the arctan 3 function for variant NoXM. All ages are in years relative to the starting age of the smallest cortical growth mark (CGM) circumference in Tibia MOR 1189. [file peerj-14-20469-s015.png]

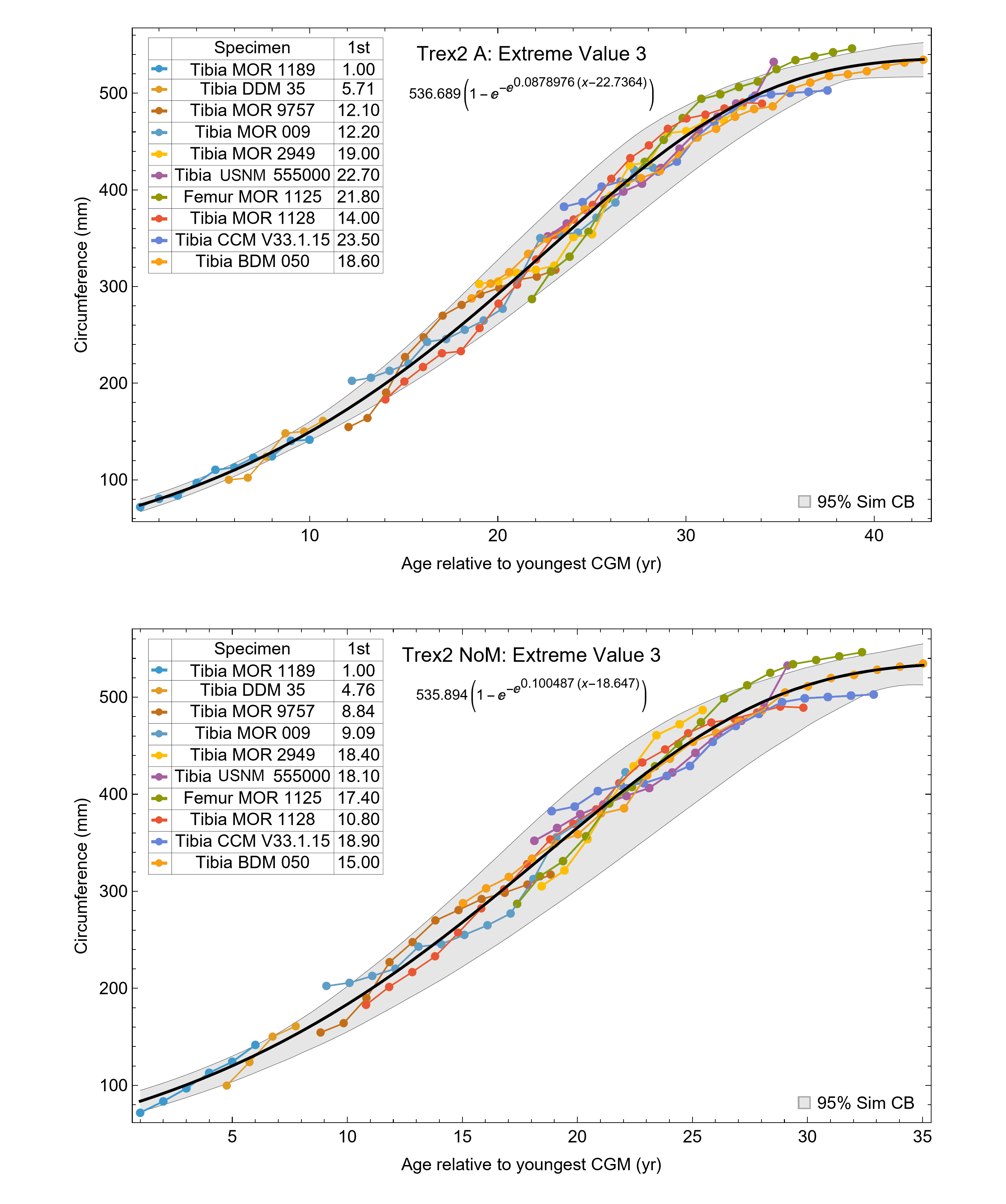

Supplement: Supplemental Information 16 — The 95% simultaneous confidence bands (CB) and best fit equations are shown, in this case the extreme value 3 function for both variants. All ages are in years relative to the starting age of the smallest cortical growth mark (CGM) circumference in Tibia MOR 1189. [file peerj-14-20469-s016.png]

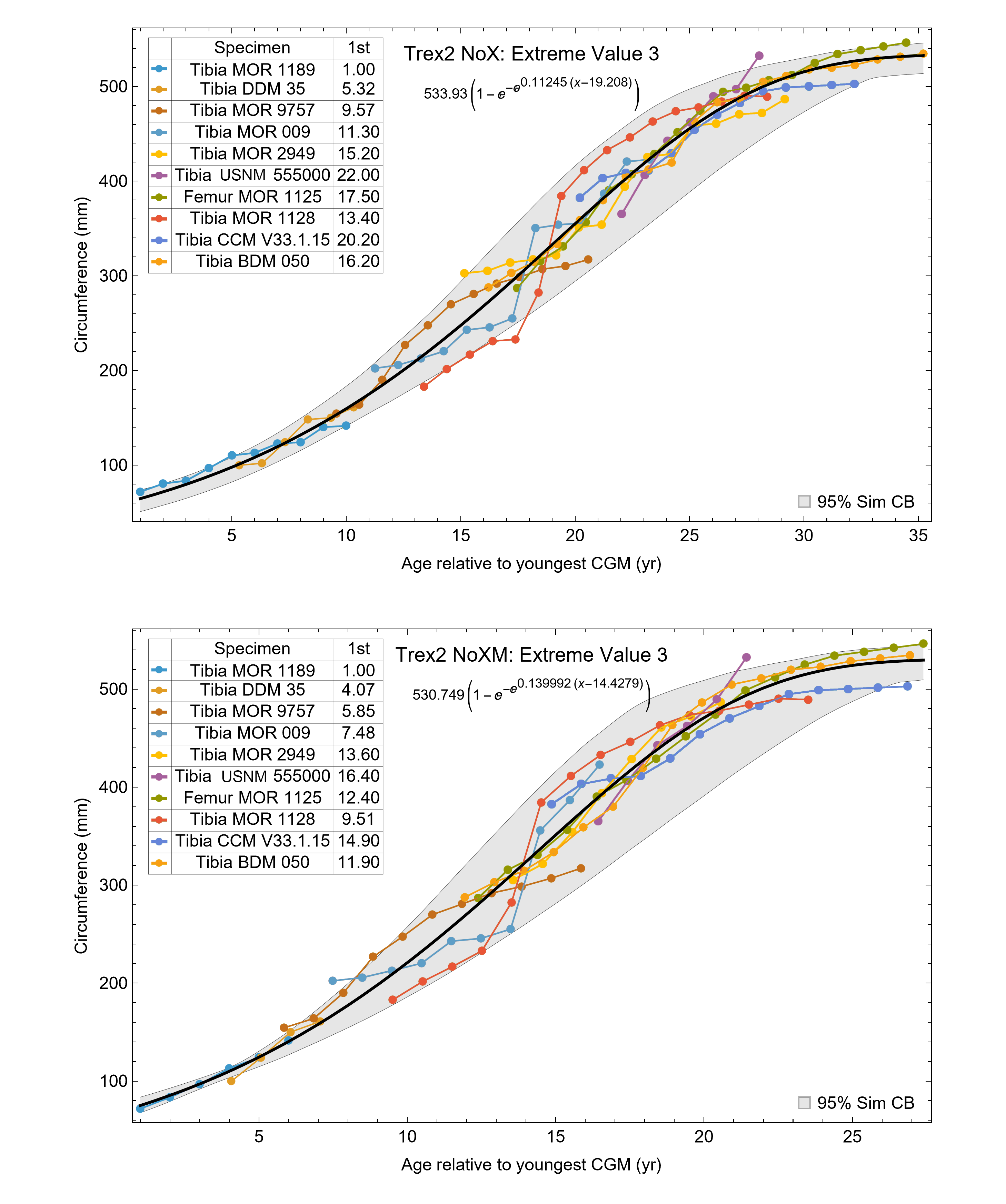

Supplement: Supplemental Information 17 — The 95% simultaneous confidence bands (CB) and best fit equations are shown, in this case the extreme value 3 function for both variants. All ages are in years relative to the starting age of the smallest cortical growth mark (CGM) circumference in Tibia MOR 1189. [file peerj-14-20469-s017.png]

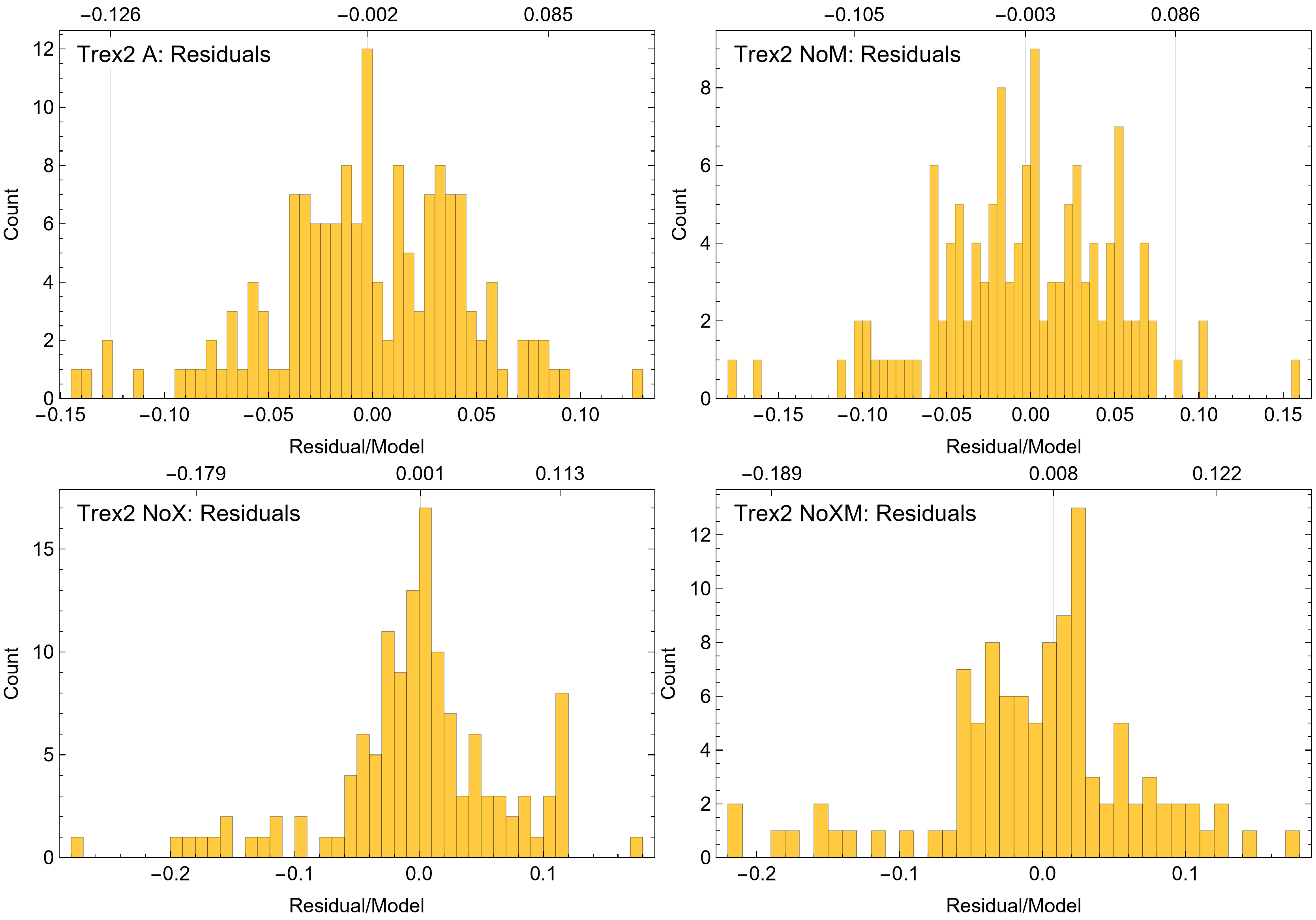

Supplement: Supplemental Information 18 — The residuals from the data series are plotted, along with their 0.025, 0.5 (median) and 0.975 quantiles. [file peerj-14-20469-s018.png]

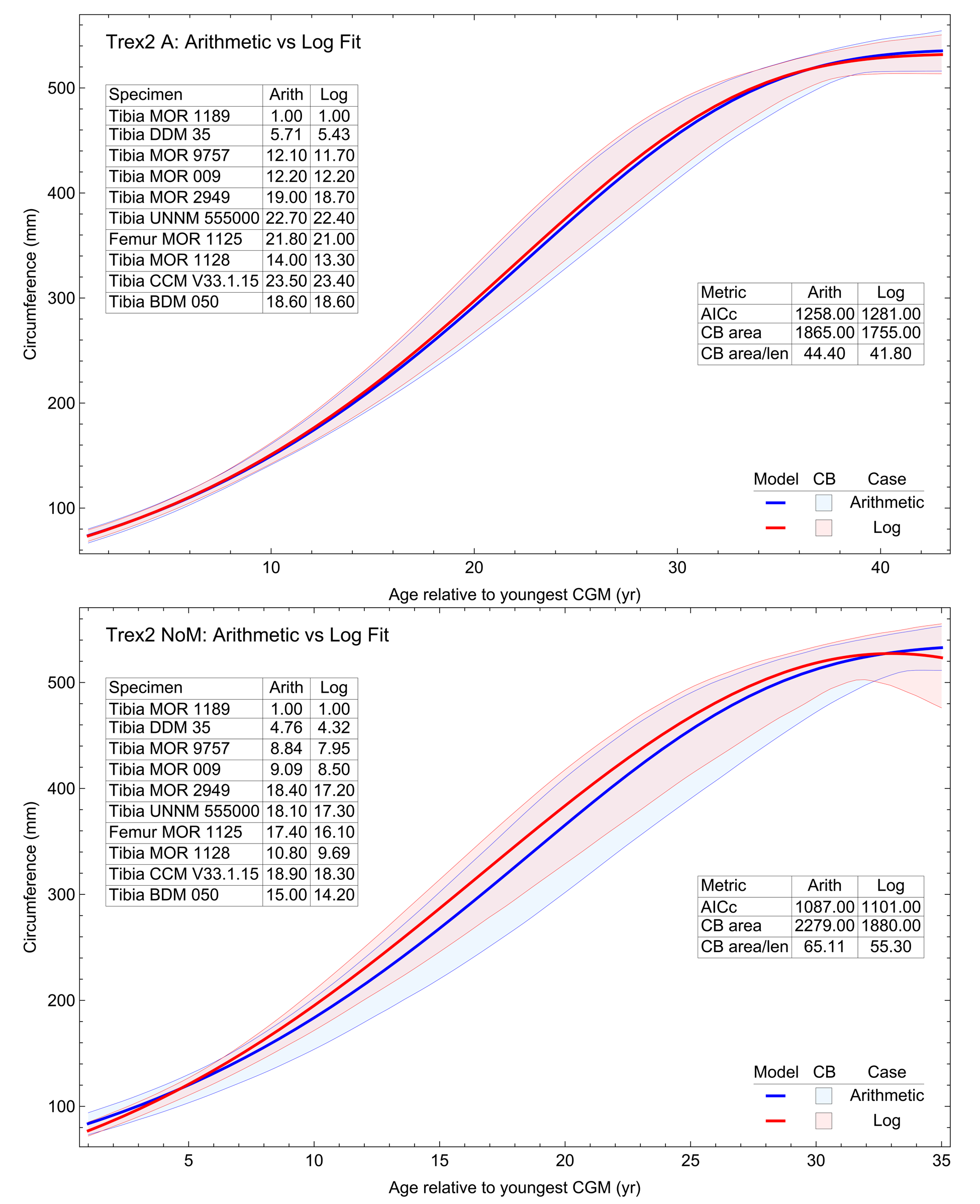

Supplement: Supplemental Information 19 — All ages are in years relative to the starting age of the smallest cortical growth mark (CGM) circumference in Tibia MOR 1189. [file peerj-14-20469-s019.png]

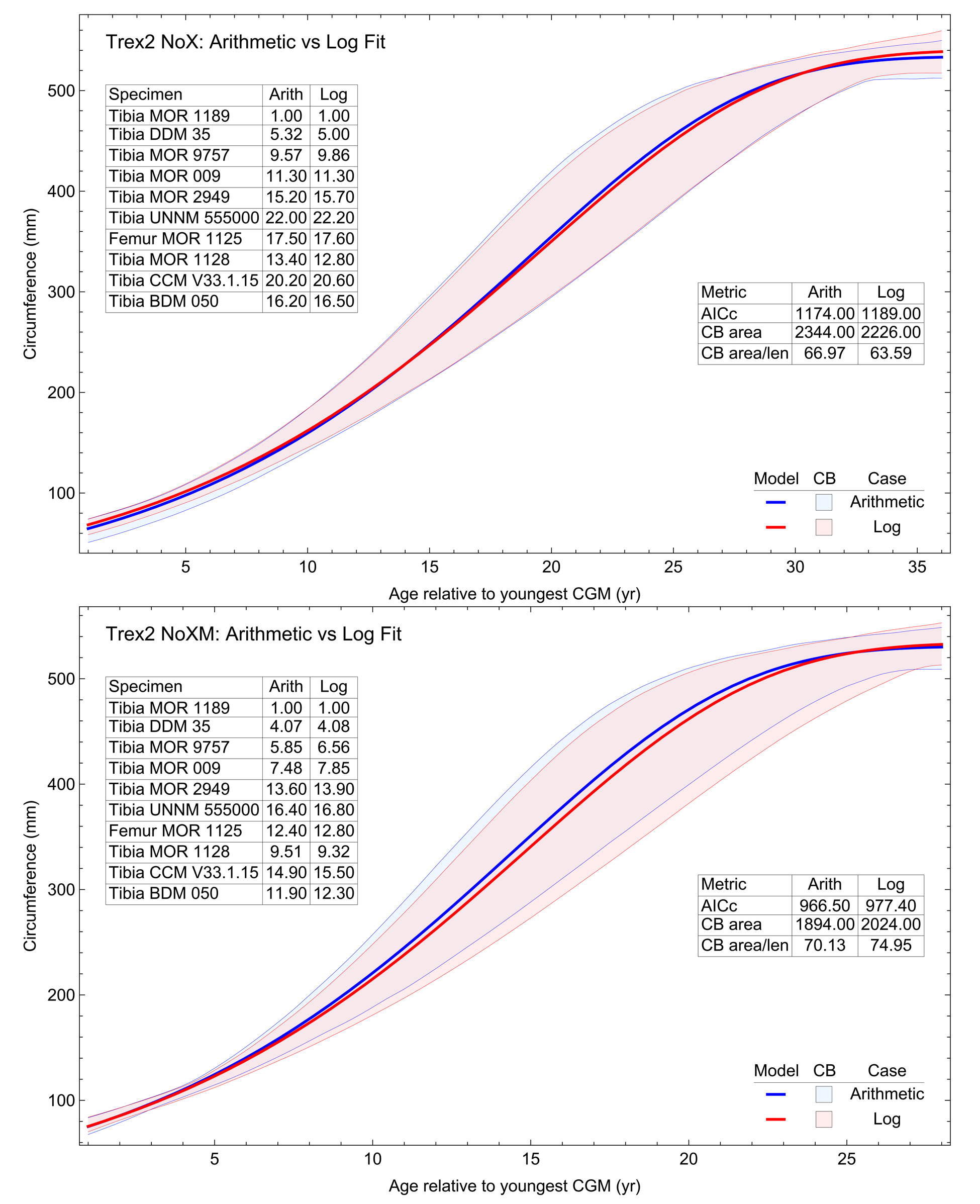

Supplement: Supplemental Information 20 — All ages are in years relative to the starting age of the smallest cortical growth mark (CGM) circumference in Tibia MOR 1189. [file peerj-14-20469-s020.png]

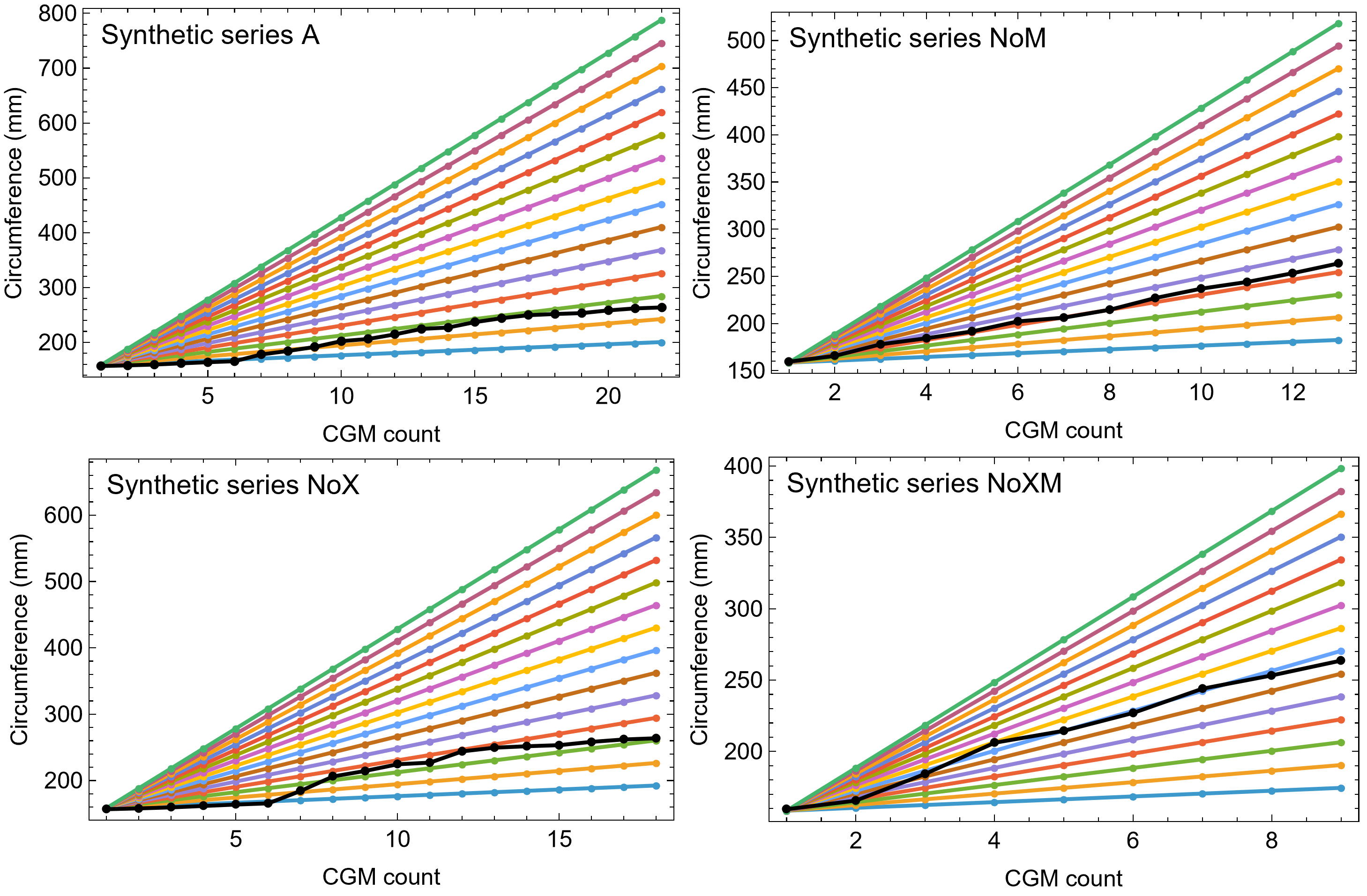

Supplement: Supplemental Information 21 — . Each panel shows a different variant (A, NoM, NoX, NoXM). The black growth series is Tibia BMRP 2006.4.4. The colored lines show linear growth series of the same length, but with constant linear growth rate ranging from 2 mm/year (bottom most line) to 30 mm/year (topmost line). Cortial growth mark (CGM) count on the x-axes, CGM circumference on the y-axes. [file peerj-14-20469-s021.png]

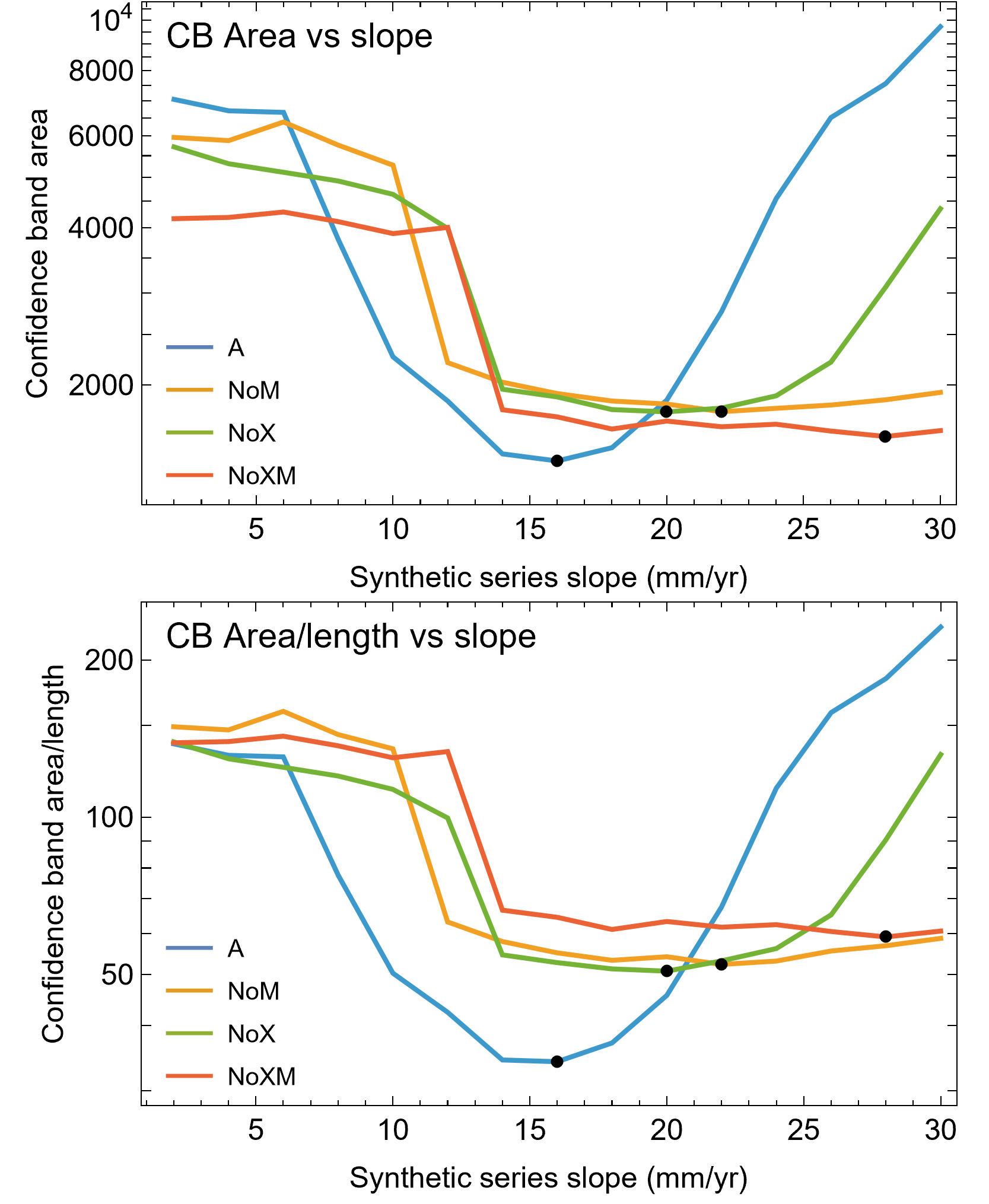

Supplement: Supplemental Information 22 — The plots show the total CB area (top panel) and CB area divided by length of CB (bottom panel) for different variants of the Trex2 dataset, plus one synthetic data series with linear growth at the specified rate. Each area (or area/length) curve reaches a minimum value at the black points. Minimum values indicate the linear growth rate that is the most compatible (of those simulated) with the given variant of the Trex2 data series. [file peerj-14-20469-s022.png]

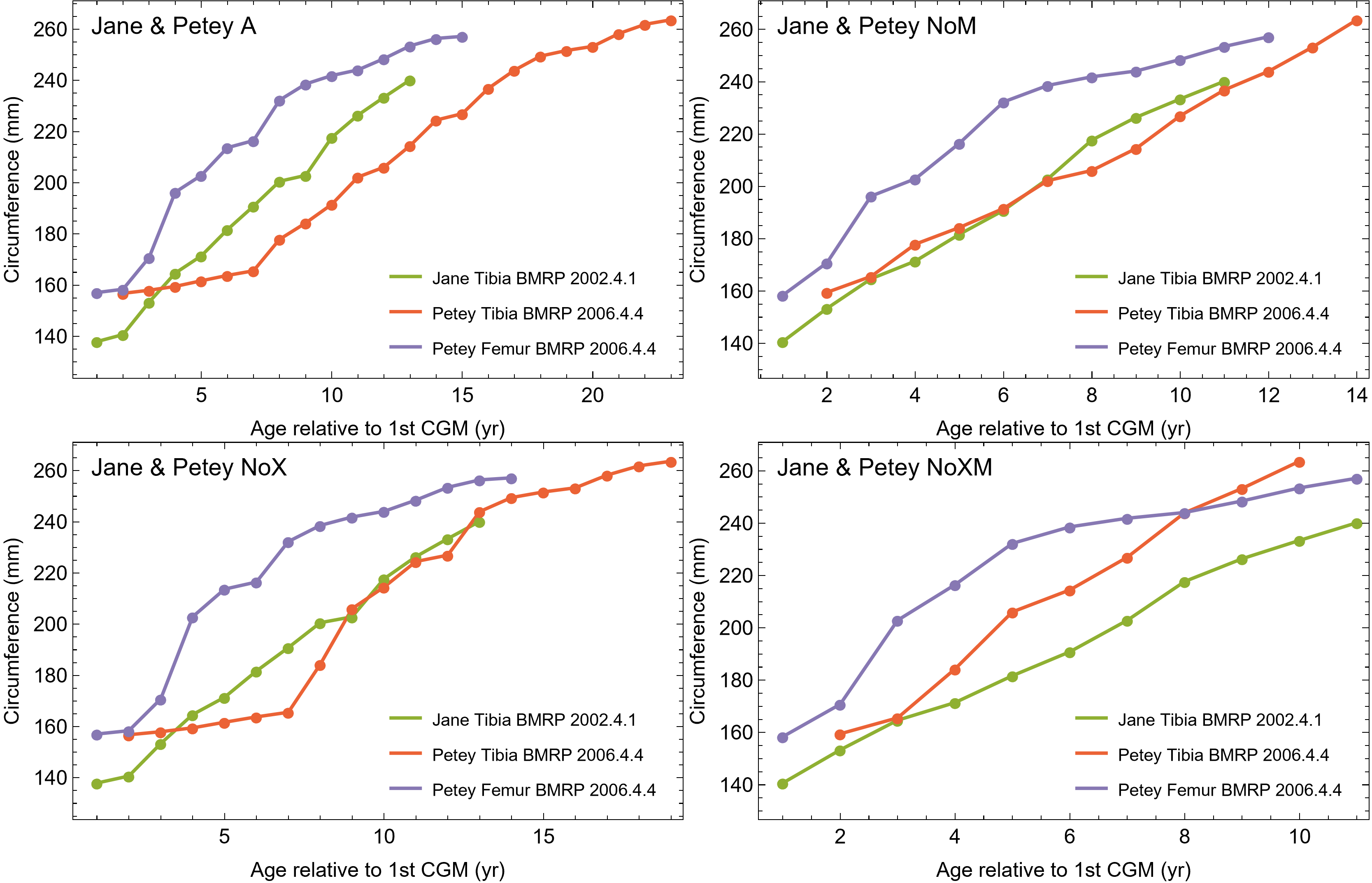

Supplement: Supplemental Information 23 — Cortical growth mark (CGM) count on the x-axes, CGM circumference (mm) on the y-axes. [file peerj-14-20469-s023.png]

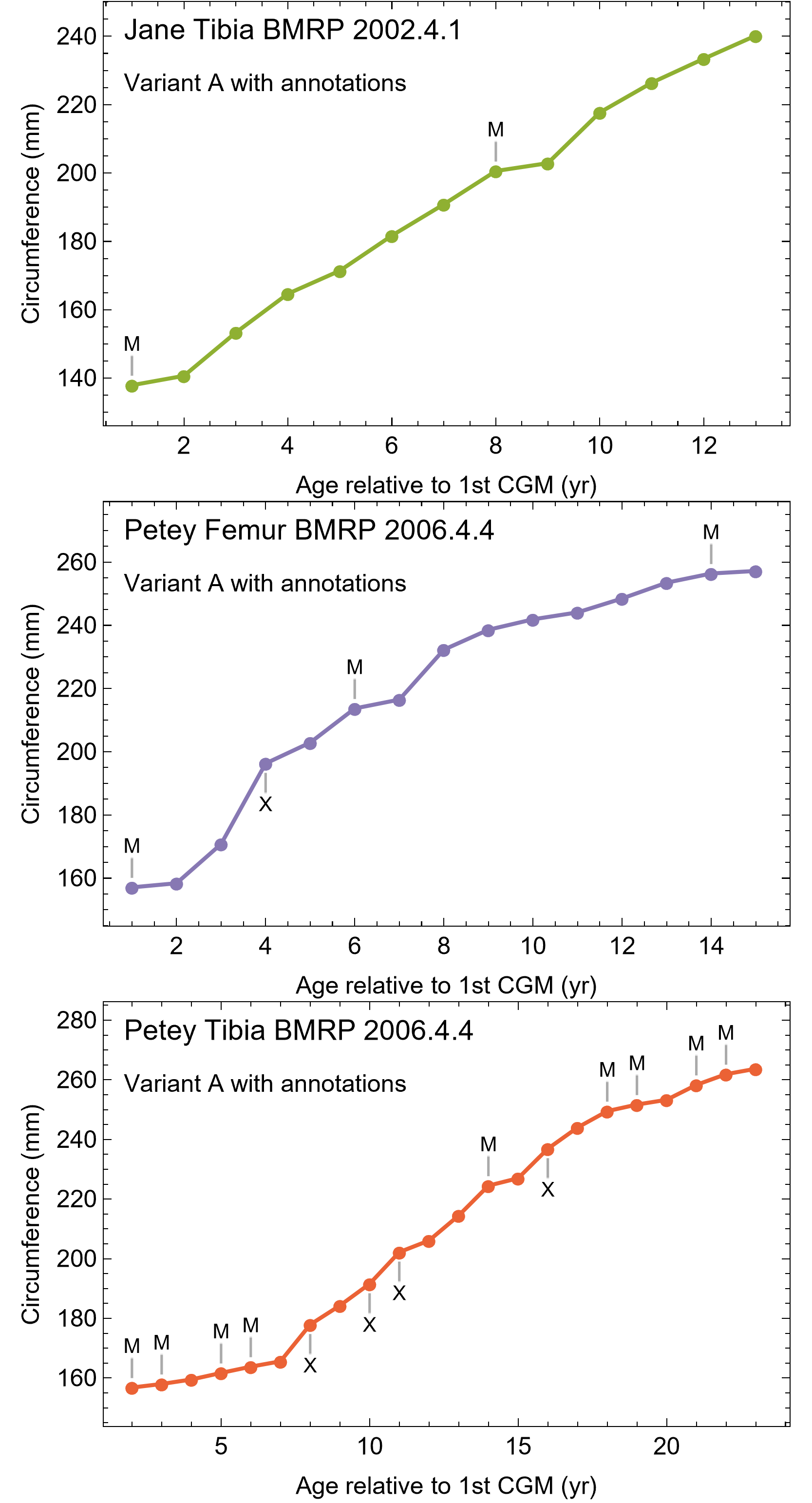

Supplement: Supplemental Information 24 — The A variant of the growth series contains all of the observed cortical growth marks (CGM). CGM marked M are multiplets which would not be present in the NoM, or NoMX variants. Those marked X are XPL CGM and would be present in the NoX and NoXM variants. [file peerj-14-20469-s024.png]
