# Supplementary material for: Prolonged growth and extended subadult development in the Tyrannosaurus rex species complex revealed by expanded histological sampling and statistical modeling
Source: PeerJ. 2026 Jan 14;14:e20469. doi: 10.7717/peerj.20469 (PMC12811967; doi:10.7717/peerj.20469)
Supplement: Supplemental Information 26 — CGM which are only visible in cross polarized light are counted in the column XPL count. CGM which are omitted from variants NoM and NoXM on the basis that they are members of multiplets are counted in column “M count”. [file peerj-14-20469-s026.docx]

**Table S2:**

**Count of cortical growth marks (CGM) visible in cross polarized light (XPL) and those comprising a multiplet (M).**

CGM which are only visible in cross polarized light are counted in the column XPL count. CGM which are omitted from variants NoM and NoXM on the basis that they are members of multiplets are counted in column “M count”.

| **Specimen** | **M count** | **XPL count** |
| --- | --- | --- |
| Tibia MOR 1189 | 4 | 0 |
| Tibia DDM 35 | 2 | 0 |
| Tibia BMRP 2002.4.1 | 2 | 0 |
| Tibia BMRP 2006.4.4 | 9 | 4 |
| Femur BMRP 2006.4.4 | 3 | 1 |
| Tibia MOR 9757 | 1 | 0 |
| Tibia MOR 009 | 3 | 4 |
| Tibia MOR 2949 | 7 | 0 |
| Tibia UNNM 555000 | 1 | 6 |
| Femur MOR 1125 | 2 | 0 |
| Tibia MOR 1128 | 1 | 5 |
| Tibia CCM V33.1.15 | 0 | 2 |
| Tibia BDM 050 | 4 | 5 |
