# Supplementary material for: Prolonged growth and extended subadult development in the Tyrannosaurus rex species complex revealed by expanded histological sampling and statistical modeling
Source: PeerJ. 2026 Jan 14;14:e20469. doi: 10.7717/peerj.20469 (PMC12811967; doi:10.7717/peerj.20469)
Supplement: Supplemental Information 27 — This table is primarily composed of sigmoidal models, but simple linear and quadratic models are also included. For the sigmoidal models the parameter is always the maximum asymptotic size, is always a location parameter that determines where on the time axis the sigmoid is located. The parameter is related to the slope (growth rate) of the model in its intermediate growth phase. [file peerj-14-20469-s027.docx]

**Table S3:**

**Arithmetic scale models fit to growth data.**

This table is primarily composed of sigmoidal models, but simple linear and quadratic models are also included. For the sigmoidal models the parameter $a$ is always the maximum asymptotic size, $c$ is always a location parameter that determines where on the time axis the sigmoid is located. The parameter $b$ is related to the slope (growth rate) of the model in its intermediate growth phase.

| **Name** | **Equation** |
| --- | --- |
| Linear 2 | $b+ax$ |
| Quadratic 3 | $c+bx+ax^{2}$ |
| Logistic 3 | $\frac{a}{1+ⅇ^{-b^{2}(-c+x)}}$ |
| Gompertz 3 | $aⅇ^{-ⅇ^{-b^{2}(-c+x)}}$ |
| Extreme Value 3 | $a \left( 1-ⅇ^{-ⅇ^{b^{2}(-c+x)}} \right)$ |
| Erfc 3 | $\frac{a}{2}\mathrm{erfc}\left( \frac{b^{2}(c-x)}{\sqrt{2}} \right),$ |
| Arctan 3 | $a\left( \frac{\tan^{-1}(b^{2}(x-c))}{\pi}+\frac{1}{2} \right)$ |
| Sech 3 | $\frac{2a}{\pi}\left( \tan^{-1}ⅇ^{\frac{\pi(x-c)}{2b^{2}}} \right)$ |
| Moyal 3 | $a \mathrm{erfc}\left( \frac{ⅇ^{-\frac{x-c}{2b^{2}}}}{\sqrt{2}} \right)$ |
| Tanh 3 | $\frac{1}{2}a(\tanh(b^{2}(x-c))+1)$ |
| Laplace 3 | $\begin{matrix} \left\{ \begin{aligned} a \left( 1-\frac{1}{2}ⅇ^{\frac{c-x}{b^{2}}} \right) \\ \frac{a}{2}ⅇ^{\frac{c-x}{b^{2}}} \end{aligned} \right. & \begin{matrix} & x\geq c \\ & \mathrm{True} \end{matrix} \end{matrix}$ |
