# Supplementary material for: Prolonged growth and extended subadult development in the Tyrannosaurus rex species complex revealed by expanded histological sampling and statistical modeling
Source: PeerJ. 2026 Jan 14;14:e20469. doi: 10.7717/peerj.20469 (PMC12811967; doi:10.7717/peerj.20469)
Supplement: Supplemental Information 28 — The models fit to conventional (arithmetic scale) data are modified for fitting to log-transformed data, in which the circumference values are log-transformed. Here we use log to mean natural logarithm. Parameter interpretation is as described for arithmetic scale models. The Laplace 3 model (Table S2) is not included because it has algorithmic issues in fitting. [file peerj-14-20469-s028.docx]

**Table S4:**

**Models fit to log-transformed growth data.**

The models fit to conventional (arithmetic scale) data are modified for fitting to log-transformed data, in which the circumference values are log-transformed. Here we use log to mean natural logarithm. Parameter interpretation is as described for arithmetic scale models. The Laplace 3 model (Table S2) is not included because it has algorithmic issues in fitting.

| **Name** | **Equation** |
| --- | --- |
| Linear 2 | $b+ax$ |
| Quadratic 3 | $c+bx+ax^{2}$ |
| Log Linear 2 | $log(b+ax)$ |
| Log Quadratic 3 | $log(c+bx+ax^{2})$ |
| Log Logistic 3 | $log(a)-log(1+ⅇ^{-b^{2}(-c+x)})$ |
| Log Gompertz 3 | $\log\left( a \right)-ⅇ^{-b^{2}(-c+x)}$ |
| Log Extreme Value 3 | $\log\left( 1-ⅇ^{-ⅇ^{b^{2}(-c+x)}} \right)$ |
| Log Erfc 3 | $\log\left( a \right)-log(2)+\log\left( \mathrm{erfc}\left( \frac{b^{2}\left( c-x \right)}{\sqrt{2}} \right) \right)$ |
| Log Arctan 3 | $\log\left( a \right)+\log\left( \frac{\tan^{-1}(b^{2}(x-c))}{\pi}+\frac{1}{2} \right)$ |
| Log Sech 3 | $\log\left( a \right)+\log\left( 2 \right)-\log\left( \pi\right)+\log\left( \tan^{-1}ⅇ^{\frac{\pi(x-c)}{2b^{2}}} \right)$ |
| Log Moyal 3 | $\log\left( a \right)+\log\mathrm{erfc}\left( \frac{ⅇ^{-\frac{x-c}{2b^{2}}}}{\sqrt{2}} \right)$ |
| Log Tanh 3 | $\log\left( a \right)-\log\left( 2 \right)+\log\left( \tanh(b^{2}(x-c))+1 \right)$ |
