# Supplementary material for: Prolonged growth and extended subadult development in the Tyrannosaurus rex species complex revealed by expanded histological sampling and statistical modeling
Source: PeerJ. 2026 Jan 14;14:e20469. doi: 10.7717/peerj.20469 (PMC12811967; doi:10.7717/peerj.20469)
Supplement: Supplemental Information 29 — The model, parameter values and 95% CI for the parameter values are shown in the table, with the CI expressed both in a high/low, and as a percentage variation on the median value. The parameters are for the sigmoidal model shown. The parameters for each of the specimen names are the estimated ages to be added to the 1 st CGM in the growth series for the specimen (in years). [file peerj-14-20469-s029.docx]

**Table S5:**

**Trex2 model parameters and their 95% confidence intervals (CI).**

The model, parameter values and 95% CI for the parameter values are shown in the table, with the CI expressed both in a high/low, and as a percentage variation on the median value. The parameters $a, b, c$ are for the sigmoidal model shown. The parameters for each of the specimen names are the estimated ages to be added to the 1^st^ CGM in the growth series for the specimen (in years).

| **Variant** | **Model** | **Parameter** | **Median** | **95% CI** | | **95% CI as %** | |
| --- | --- | --- | --- | --- | --- | --- | --- |
| A | Extreme Value 3  $a \left( 1-ⅇ^{-ⅇ^{b^{2}(x-c)}} \right)$ | $a$ | 536.689 | 522.958 | 553.986 | -2.56% | 3.22% |
|  |  | $b$ | 0.296 | 0.281 | 0.310 | -5.36% | 4.64% |
|  |  | $c$ | 22.736 | 20.950 | 25.074 | -7.86% | 10.28% |
|  |  | Tibia MOR 1189 | 0.000 | 0.000 | 0.000 | na | na |
|  |  | Tibia DDM 35 | 4.709 | 3.411 | 5.820 | -27.55% | 23.61% |
|  |  | Tibia MOR 9757 | 11.070 | 9.532 | 12.719 | -13.90% | 14.89% |
|  |  | Tibia MOR 009 | 11.249 | 9.697 | 12.943 | -13.80% | 15.06% |
|  |  | Tibia MOR 2949 | 18.012 | 16.190 | 20.051 | -10.11% | 11.32% |
|  |  | Tibia UNNM 555000 | 21.668 | 19.845 | 23.864 | -8.41% | 10.13% |
|  |  | Femur MOR 1125 | 20.817 | 18.604 | 23.678 | -10.63% | 13.75% |
|  |  | Tibia MOR 1128 | 13.034 | 11.639 | 15.268 | -10.70% | 17.15% |
|  |  | Tibia CCM V33.1.15 | 14.519 | 12.567 | 16.801 | -13.44% | 15.72% |
|  |  | Tibia BDM 050 | 17.611 | 15.897 | 19.478 | -9.73% | 10.60% |
| NoM | Extreme Value 3  $a \left( 1-ⅇ^{-ⅇ^{b^{2}(x-c)}} \right)$ | $a$ | 535.894 | 516.521 | 556.052 | -3.62% | 3.76% |
|  |  | $b$ | 0.317 | 0.298 | 0.334 | -6.05% | 5.46% |
|  |  | $c$ | 18.647 | 16.711 | 21.310 | -10.38% | 14.28% |
|  |  | Tibia MOR 1189 | 0.000 | 0.000 | 0.000 | na | na |
|  |  | Tibia DDM 35 | 3.757 | 2.000 | 5.328 | -46.78% | 41.81% |
|  |  | Tibia MOR 9757 | 7.836 | 6.428 | 9.866 | -17.98% | 25.90% |
|  |  | Tibia MOR 009 | 8.093 | 6.420 | 10.283 | -20.67% | 27.06% |
|  |  | Tibia MOR 2949 | 17.444 | 15.281 | 20.409 | -12.40% | 17.00% |
|  |  | Tibia UNNM 555000 | 17.129 | 15.127 | 19.677 | -11.69% | 14.87% |
|  |  | Femur MOR 1125 | 16.380 | 14.355 | 19.445 | -12.36% | 18.71% |
|  |  | Tibia MOR 1128 | 9.821 | 8.365 | 12.116 | -14.82% | 23.37% |
|  |  | Tibia CCM V33.1.15 | 9.889 | 7.709 | 12.438 | -22.04% | 25.77% |
|  |  | Tibia BDM 050 | 14.027 | 12.281 | 16.343 | -12.45% | 16.51% |
